# Low-Temperature Properties of Silver

**DOI:** 10.6028/jres.100.012

**Published:** 1995

**Authors:** David R. Smith, F. R. Fickett

**Affiliations:** National Institute of Standards and Technology, Boulder, CO 80303

**Keywords:** conductivity, cryogenic, elastic, electrical, magnetic, mechanical, optical, properties, review, resistivity, silver, superconductor, thermal, thermodynamic, transport

## Abstract

Pure silver is used extensively in the preparation of high-temperature superconductor wires, tapes, films, and other configurations in which the silver not only shields the superconducting material from the surrounding materials, but also provides a degree of flexibility and strain relief, as well as stabilization and low-resistance electrical contact. Silver is relatively expensive, but at this stage of superconductor development, its unique combination of properties seems to offer the only reasonable means of achieving usable lengths of conductor. In this role, the low-temperature physical (electrical, thermal, magnetic, optical) and mechanical properties of the silver all become important. Here we present a collection of properties data extracted from the cryogenic literature and, to the extent possible, selected for reliability.

## 1. Introduction

Most applications of high-temperature superconductors involve the use of silver. In thin-film devices, it serves as an electrical contact with acceptably low contact resistance, on the order of 10^−8^ Ω·cm^2^. In large-scale devices the contact application is also important; the contact resistance can be higher, but the contact area is large. In conductor applications silver also provides (1) containment for the precursor powder mix during mechanical processing; (2) containment for the powder while allowing passage of oxygen during heat treatment to form the superconducting structure; (3) stabilization of the conductor in operation by providing an alternate current path and a thermal path to the coolant; (4) providing mechanical strength to the finished conductor; and (5) providing internal strain relief for the brittle superconductor when mixed as part of the powder. In low-temperature superconductors, most of these roles are carried out by copper. Silver, however, is required in high-temperature superconductors because of its unique ability to allow passage of diffusing oxygen. The low-temperature properties of silver also make it an attractive choice in applications that are not directly related to superconductivity. These properties are its high thermal and electrical conductivities, diamagnetism, high reflectance, and low emittance.

Over the past two decades, we have produced two versions [1, 2] of a wall chart presenting the cryogenic properties of copper. This chart has been popular with the low-temperature superconductor community. With the advent of high-temperature superconductors, we decided that a similar wall chart of cryogenic properties of silver would be valuable. This new chart, *Cryogenic Properties of Silver*, became available for distribution in January 1994 [3]. Limitations on the size of the chart did not allow us to present all the cryogenic-property data which were found in our review of the literature. Furthermore, the chart provided very little room for presentation of tabular data or commentary. Providing that additional information is the purpose of this paper.

Here we present figures and tables of the electrical, magnetic, mechanical, optical, thermal, and thermodynamic properties of pure silver, culled from more than 200 documents covering a period of over 60 years. While we concentrated on these properties, we have also included information in areas such as metal physics and effects of irradiation by high-energy particles on resistivity.

In general, we treated only pure silver. However, one paper on coin silver was included because this alloy is commonly available and might be of use at cryogenic temperatures. Also, mention is made (see Sec. 3.1, Ref. [137]) of a new material, a dispersion-hardened silver alloy which can provide additional strength and resistance to thermal shock, while maintaining acceptable thermal and electrical properties. The door to the properties of other alloys of silver was not cracked further open.

This work is not a complete survey of all the literature available on silver. While we made a strong effort to identify those papers of most use to workers interested in the cryogenic applications of silver, resources did not permit an exhaustive search of all the current literature. We believe that we located most, if not all, of the important sources of available data. Each bibliographic reference to a paper has been annotated by a capsule summary of the content specifically related to cryogenic applications of silver. Before the reader uses any data for engineering design or other critical applications, the reader is strongly urged to consult the original reference(s) for complete details of the conditions of measurement.

Some mention needs to be made regarding the techniques by which data were transferred from the graphs and plots in the original documents into the form presented here. Where tabular data were available there was no problem, and the plots were made directly. However, most of the documents provided data in graphical form, and often only as very small graphs and of quality less than that available now in the era of desktop publishing. In those cases, a combination of enlargement by photocopier, use of a graphics tablet, and commercial plotting software allowed extraction and smoothing of the data while maintaining the accuracy of the original plot. Because of the increased size of many of the plots, some curves show a minor lack of smoothness. All such variations lie within the accuracy of the data as plotted in the original publication.

## 2. Properties Data

In the following descriptions of tables and figures, a bullet (•) at the beginning of a paragraph denotes a description corresponding to a table or figure used in the wall chart, *Cryogenic Properties of Silver* [3]. Those tables or figures were selected for inclusion in the chart because of widest appeal or interest. For each graph or table in this paper there is always a corresponding bibliographic entry in Sec. 3, similarly marked with a dagger (†) and index number. The additional documents in the bibliography, under the topics listed here, contain further information on these properties.

### 2.1 General

- The fundamental physical and chemical properties of silver are summarized in Table 1. It is fairly easy to find more than one secondary source for many of the data in the table, and there are usually small (within one or two percent) disagreements among the various values for a given property. Where multiple values were found, a representative (most frequently occurring) value was chosen for listing in this table.
- Conversion factors between SI units and other systems, such as traditional (“British”) units are given in Tables 2a and 2b.

### 2.2 Electrical Resistivity

**Fig. 1:**
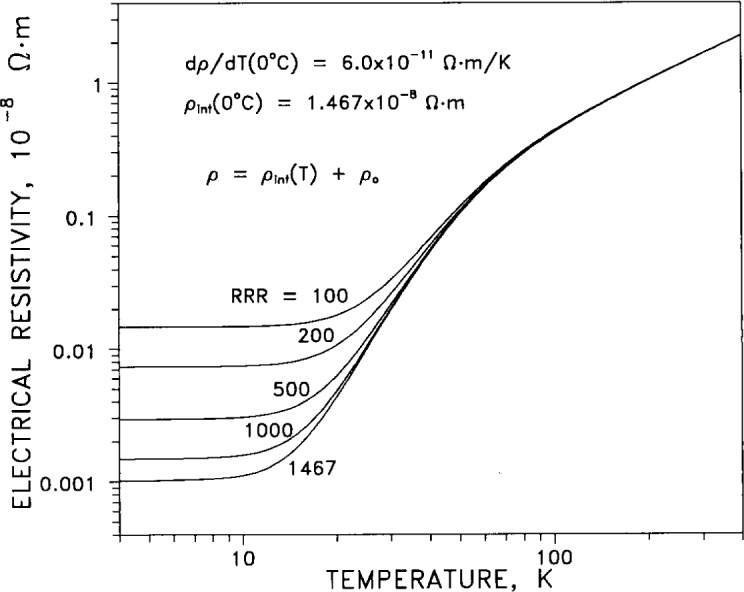
Electrical resistivity *ρ* as a function of temperature; *ρ*_int_ is the temperature-dependent intrinsic resistivity, and *ρ*_0_ is the residual (0 K) resistivity, due to impurities. Residual resistivity ratio *RRR* is defined as *ρ*(273 K)/*ρ*(4.2 K). [67] (A number in square brackets following each figure caption denotes a bibliographic reference number (Sec. 3) to the source paper.)

**Fig. 2:**
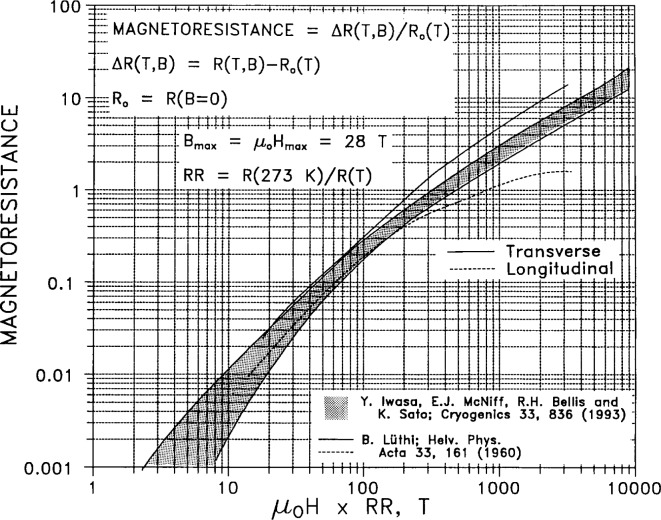
Kohler plot of magnetoresistance (as a function of product of magnetic field *H* and resistance ratio *RR).* [98, 100]

- Matthiessen’s rule assumes that the total electrical resistivity *ρ*_el_ is well approximated by a sum of two terms. The first is a temperature-dependent intrinsic term *ρ*_int_(*T*), which is zero at absolute zero. The second term is a residual term *ρ*_0_ due to the effect of impurities and crystal defects, and does not vanish at absolute zero. In Fig. 1 the total electrical resistivity of silver as a function of temperature is plotted for four different values of *RRR* (residual resistance ratio; a measure of impurity content), and a value for *ρ*_int_ at 0 °C is given. For metals in electrical applications, values for *RRR* are a more sensitive measure of purity than total content of chemical impurities. Above 80 K the resistivity of dilute alloys of silver is independent of impurity content, whereas below 80 K the resistivity is sensitive to impurity content. At room temperature the variation of resistivity with temperature is nearly linear.
- Table 3 lists the change in electrical resistivity per “atomic percent” (mole fraction) of alloying element for 20 different metallic elements in pure silver. The effect of alloying small amounts of impurities with silver is to increase its resistivity. The change, initially linear with very small increases in concentration, is nonlinear as larger amounts of alloying element are added. The transition point to nonlinear dependence on concentration is different for different impurities. Hence the values listed in the table should be used only as a guide, and then very cautiously. One can assume without large error that the effects of several impurities are additive in small quantities.
- Magnetoresistance, the dependence of electrical resistivity on applied magnetic field, is given in Fig. 2 as a Kohler plot. For many polycrystalline metals, data covering wide ranges of temperature and metallic purity reduce to a single curve for the transverse case (field normal to current), and data for many different metals will fall on the curve. This is known as Kohler’s rule. However, it is frequently not obeyed, and the silver data in particular seem not to obey the rule. Regardless, application of relatively modest magnetic fields can cause large changes in resistance at low temperatures. The longitudinal magnetoresistance, measured with the field parallel to the current, saturates for most metals at a relatively low value, as seen here. In single-crystal specimens, the magnetoresistance is a strong function of crystal orientation, and measurements of magnetoresistance are used to determine the detailed topology of the Fermi surface.

**Fig. 3:**
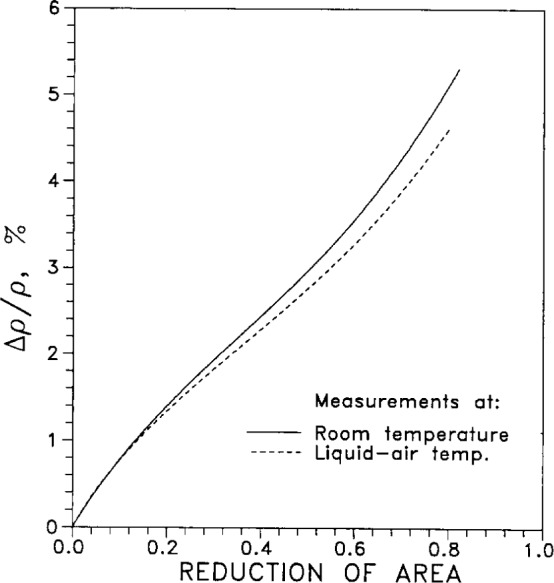
Relative change in electrical resistivity due to cold working at room temperature and liquid-air temperature, as a function of reduction of area. [81]

Figure 3 plots the relative change in resistivity due to cold working (increasing the number of defects in the specimen; the reduction in the cross-sectional area of specimen accompanying the cold working is taken into account in computing the resistivity). This figure shows that the dependence on cold working varies somewhat with temperature. That is, Matthiessen’s rule does not strictly apply. Cold working should affect only the number of geometrical defects such as vacancies and interstitials, which are independent of temperature (until annealing begins to occur, at temperatures well above room temperature); the ideal temperature-dependent intrinsic resistivity *ρ*_int_(*T*) due only to lattice vibrations should not be affected by cold working.

**Fig. 4:**
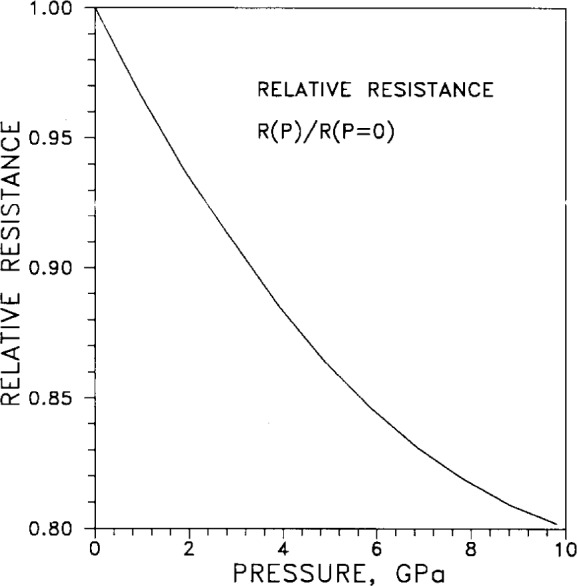
Relative resistance *R* (*P*)/*R* (*P*=0) as a function of pressure *P*. [111]

Electrical resistance depends weakly on applied pressure. The relative resistance *R*(*P*)*R*(*P*=0), normalized to unity at “zero pressure,” is plotted in Fig. 4. Both the relative resistance and the relative volume of silver, also normalized to unity at “zero pressure,” are tabulated in Table 4. One assumes that “zero pressure” is, in practice, atmospheric pressure, about 100 kPa or 10^−4^ GPa; the error in this assumption is minuscule.

**Fig. 5:**
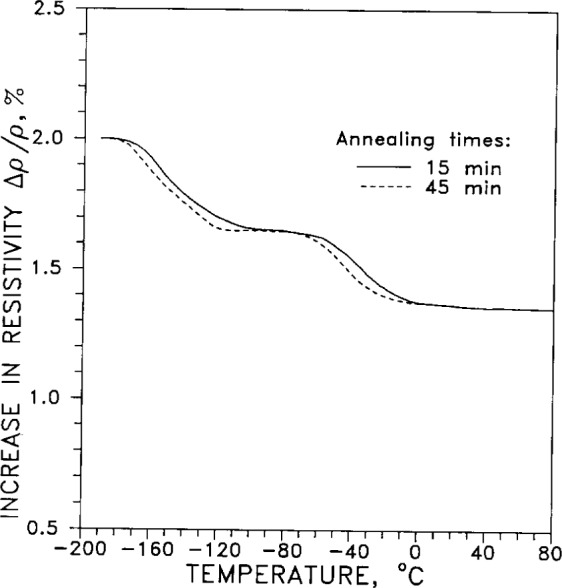
Isochronal recovery of resistivity, for wires deformed in tension at −183 °C (90 K), as a function of temperature. Recovery activation energies are: “low temperature,” 0.18 eV; “high temperature,” 0.69 eV. [66]

Figure 5 plots the isochronal recovery of resistivity of silver wires after deformation under tension. After the deformation, carried out at −183 °C (90 K), the temperature of the wire was raised by a constant amount and held at that temperature for a fixed period of time. The holding time was the same (“isochronal”) for all annealing temperatures. From the shape of the curve, the two values for energy of activation of recovery, 0.18 eV and 0.69 eV, were deduced.

**Fig. 6:**
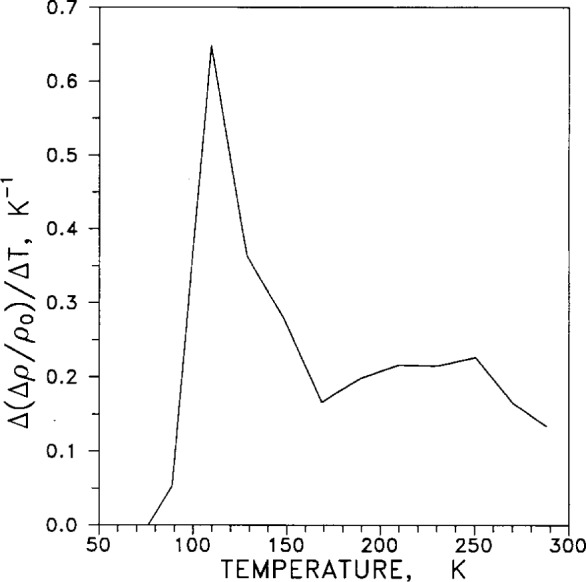
Differential fractional isochronal annealing after torsional deformation at 77 K, as a function of temperature. The specimen (“99.999 % pure”) was annealed for 360 s at each temperature, over 20 K intervals. The peaks at 110 K and at 250 K identify major annealing stages. The residual resistivity *ρ*_0_ was measured at 4.2 K. The original paper had, as the ordinate label, Δ(Δ*ρ*/Δ*ρ*_0_)/Δ*T.* However, *ρ*_0_ should not change in the given experiment. We believe the correct ordinate is Δ(Δ*ρ*/Δ*ρ*_0_)/Δ*T*, the change in fractional recovery of resistivity with temperature. [30]

Another study of isochronal annealing at 77 K gave “resistivity differential curves of fractional isochronal annealing” after torsional deformation, for silver and silver alloys. Figure 6 gives the plot for pure silver. The peaks in the curve at 110 K and 250 K are interpreted as major annealing stages. (We question whether the ordinate is correctly given as Δ(Δ*ρ*/Δ*ρ*_0_)/Δ*T* in the original paper, because *ρ*_o_ should not change in the given experiment. The ordinate should rather be Δ(Δ*ρ*/*ρ*_0_)/Δ*T*, the change in fractional recovery of resistivity with temperature.)

**Fig. 7:**
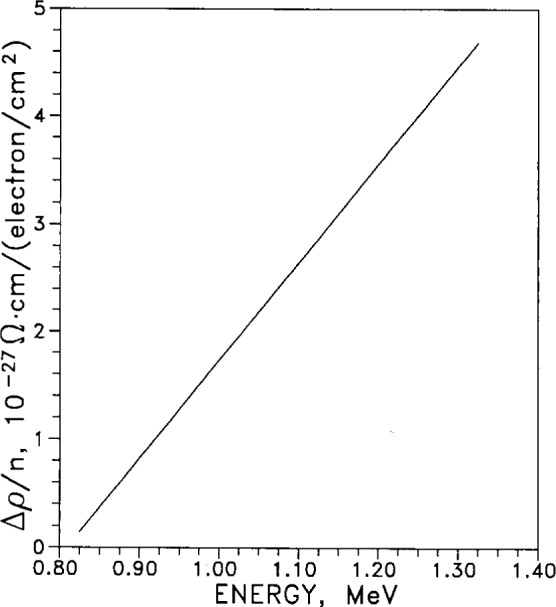
Ratio of resistivity change Δ*ρ* to electron flux *n* (electrons per unit area) as a function of average bombarding energy. [86]

The use of superconducting magnets in applications involving high-energy particle beams motivated the inclusion of Fig. 7. This shows the relative change in resistivity per unit electron flux, as a function of average energy of the bombarding electrons. The bombardment was performed at specimen temperatures of 20.4 K.

### 2.3 Thermal Properties

#### 2.3.1 Thermal Conductivity

**Fig. 8:**
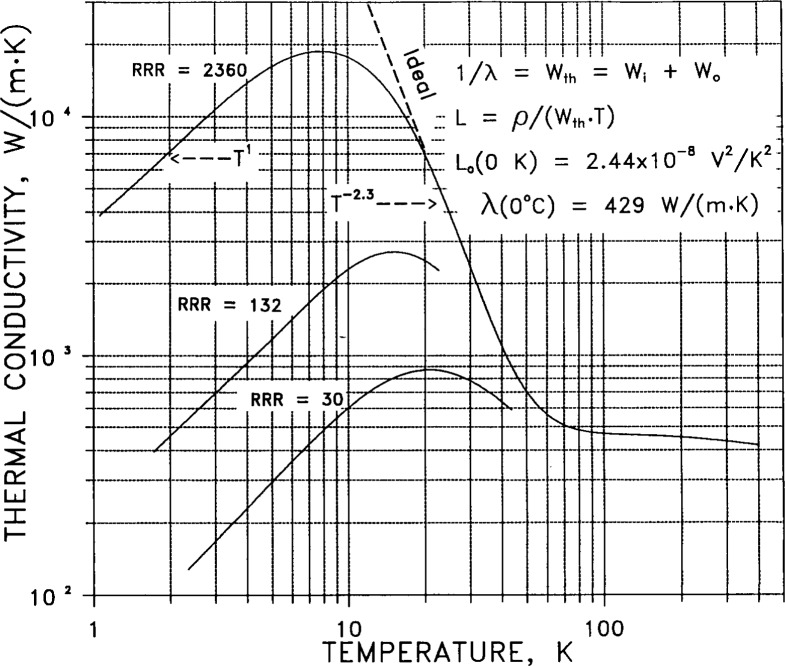
Thermal conductivity λ as a function of temperature for residual resistance ratios *(RRR)* of 30, 132, and 2360. [161]

Thermal conductivity *λ* is the measure of steady-state conductive flow of heat. Figure 8 gives the thermal conductivity of silver for various values of *RRR* (residual resistance ratio, a measure of impurity content). The expression 1/*λ*=*W*_th_ =*W*_i_ + *W*_0_ is the thermal analog of Matthiessen’s rule; here *W*_th_ is the total thermal resistance, *W*_i_ is the ideal temperature-dependent resistance, and *W*_0_ is the resistance due to impurities. The Lorenz number *L*=*ρ*_el_/(*W*_th_ • *T*), where *ρ*_el_ is electrical resistivity and *T* is absolute temperature, is theoretically a constant for all metals at absolute zero. Theory predicts that *λ* should vary linearly with temperature at very low temperatures (below the three conductivity peaks near 10 K). The slope immediately above the peak is observed empirically to vary approximately as *T*^−2.3^. This gives the slope at which the conductivity would continue to rise with decreasing temperature in the absence of any physical defects or chemical impurities (thermal resistance decreasing to zero at absolute zero). The conductivity is observed empirically to become independent of impurity content above about 70 K.

**Fig. 9:**
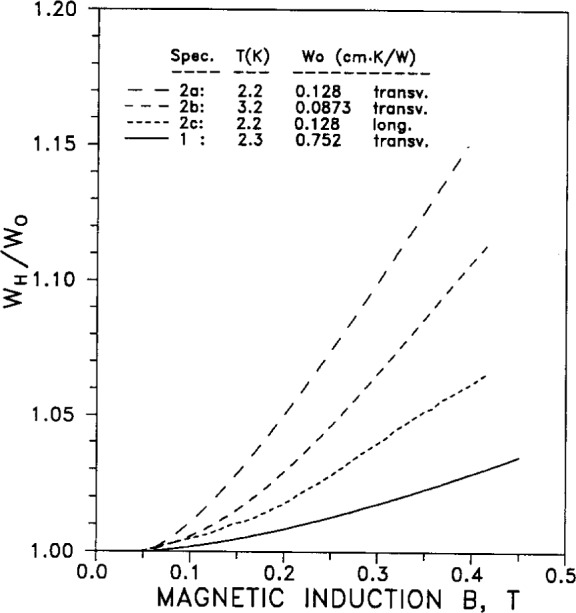
Normalized thermal resistance *W_H_/W*_O_ as a function of magnetic induction; *W_H_* is the thermal resistance in a magnetic field *H*, oriented longitudinally or transverse to the specimen axis. [172]

Thermal conductivity can vary with magnetic field, due to the effect of the field on the conduction electrons. This magneto-thermal resistance is shown in Fig. 9 in the form of thermal resistance *W*, normalized to the zero-field resistance *W*_0_=*W*(*B*=0), as a function of magnetic induction *B*. The four curves are for different values of *W*_0_ and specimen orientation (longitudinal or transverse to the thermal current); the temperatures of the measurement were also slightly different for each experiment.

#### 2.3.2 Thermal Diffusivity

**Fig. 10:**
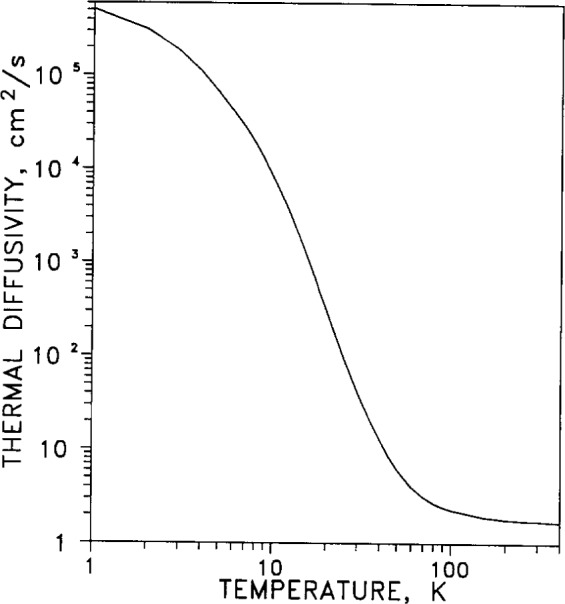
Thermal diffusivity as a function of temperature. The diffusivity *α = λ/ρC_p_* was calculated from the thermal conductivity *λ*, density *ρ*, and specific heat *C*_p_, as functions of temperature. The specimen was described as “well-annealed;” *RRR*=2360. [175]

Thermal diffusivity *a* is a measure of the transient flow of heat through a material. (The symbol a is chosen here for diffusivity to avoid confusion between diffusivity and thermal expansion, both of which are conventionally represented by the symbol *α*). The relation *a*=*λ*/(*C_p_*·*ρ*) defines the thermal diffusivity in terms of the thermal conductivity *λ*, the specific heat *C_p_* and the density *ρ*. Fig. 10 shows the diffusivity as a function of temperature.

#### 2.3.3 Thermal Expansion

**Fig. 11:**
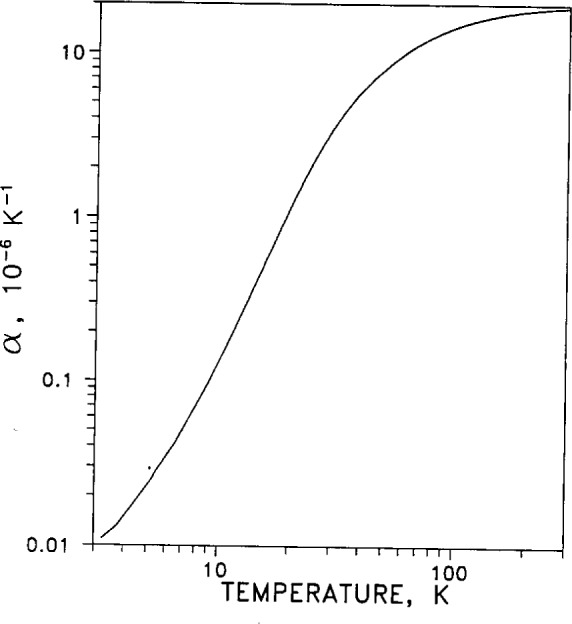
Linear thermal expansion coefficient *α* = Δ*L*/(*L*_293_ Δ*T*) as a function of temperature *T.* [181]

The linear thermal expansion coefficient measures the one-dimensional expansion of a material with changing temperature. It is defined by *α* =(d*L*/d*T*)/*L*_0_=dln(*L*)/d*T*, where *L*_0_ is the length of the specimen at a reference temperature, conventionally taken to be 293 K (20 °C). For silver it varies with temperature as shown in Fig. 11. Because α varies by several orders of magnitude between liquid-helium temperature and room temperature, the curve is given as a log-log plot. The volumetric expansion coefficient, γ=dln(*V*)/d*T*, measures the three-dimensional variation of volume with temperature, and is well approximated by γ =3*α*. The variation of density *ρ* with temperature is given by *ρ*(*T*)=*m*/*V*(*T*)=*m*/(*V*_0_(1+γ(*T*)), where *m* is the mass of the specimen. The areal, or two-dimensional, thermal expansion coefficient is *β* =dln(*A*)/d*T* and is well approximated by *β* =2*α*.

#### 2.3.4 Thermoelectric Power

**Fig. 12:**
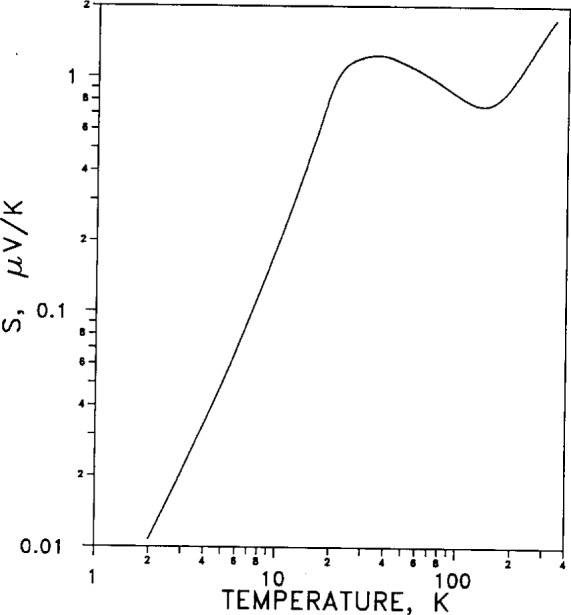
Absolute thermoelectric power *S* as a function of temperature. [202]

The absolute thermoelectric power *S*=d*E*/d*T* is a measure of the rate of change of absolute thermoelectric emf *E* with temperature. An electrical circuit involving two junctions of silver (of thermoelectric power *S*_a_) with another metal (of thermoelectric power *S*_b_) will generate an output emf per degree equal to *S*_a_−*S*_b_ when the junctions are at different temperatures. Because of its strong dependence on temperature, it is given on a log-log plot in Fig. 12.

### 2.4 Magnetic Properties

#### 2.4.1 Hall Coefficient

**Fig. 13:**
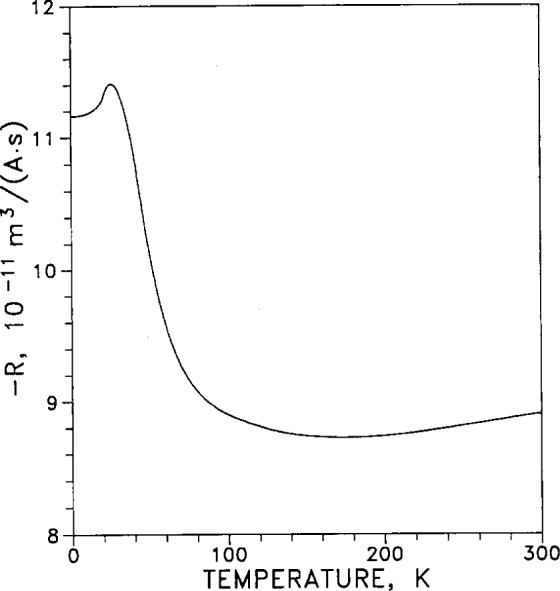
Hall coefficient *R*(*R* < 0) as a function of temperature in a magnetic field of 1.5155 T. The specimen was polycrystalline, annealed, and “99.9999 *%* pure.” [88]

The Hall coefficient −*R*_H_(*T*) is plotted in Fig. 13 as a function of temperature for silver in a magnetic induction *B*=1.5155 T. (Because electrons are the charge carriers in silver, the Hall coefficient for silver is negative; so −*R*_H_ is plotted vertically upward).

**Fig. 14:**
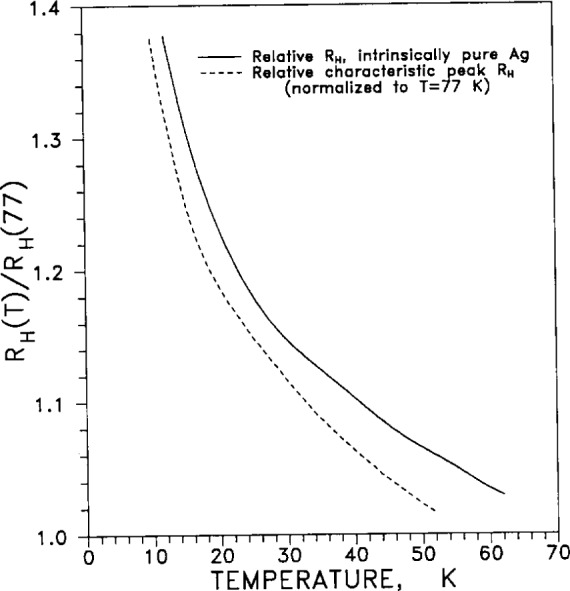
Low-field (*B* = 8.5 mT) relative Hall coefficient, *R_H_*(*T*)/*R_H_* (77 K), as a function of temperature. [89]

The low-field dependence with temperature of the relative Hall coefficient *R*_H_(*T*), normalized to its value at 77 K, is given in Fig. 14.

**Fig. 15:**
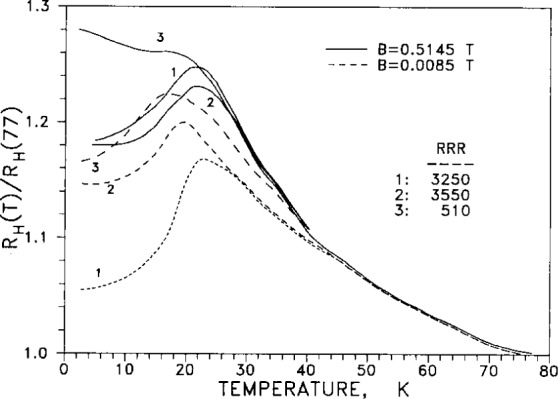
Relative Hall coefficient, *R_H_(T)/R_H_*(77 K), for magnetic inductions *B* = 0.5145 T and *B* = 0.0085 T, as a function of temperature, for three different *RRR* values. [89]

In Fig. 15, the temperature dependence of the same normalized (relative) Hall coefficient is graphed for two different values of applied magnetic induction (solid lines: *B*=0.5145 *T*; dashed lines: *B*=8.5 m*T*). For each value of magnetic field, the normalized coefficient is graphed for three different values of *RRR* (residual resistance ratio, a measure of impurity content), values of 510, 3250, and 3550).

**Fig. 16:**
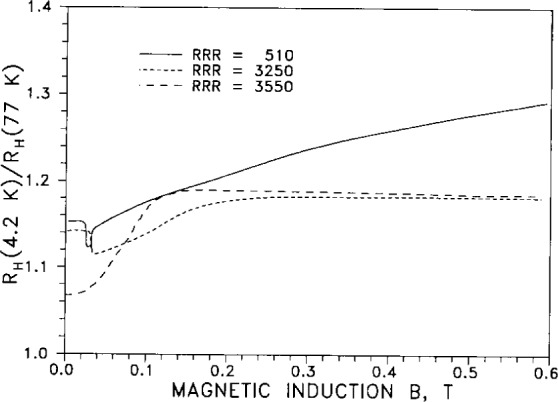
Hall coefficient at 4.2 K, *R*_H_(4.2)/*R*_H_(77 K), relative to the value at 77 K, as a function of magnetic induction, for three different *RRR* values. [89]

Figure 16 displays the relative Hall coefficients at 4.2 K, normalized to the value at 77 K, as a function of magnetic induction *B* and *RRR* value. These are the same specimens (and same *RRR* values) as in Fig. 15. The relative Hall coefficients for the specimens having *RRR*=3250 (specimen 1) and 3550 (specimen 2) show saturation at magnetic inductions above 0.2 *T*, while that for the low-purity (specimen 3: *RRR*=510) did not saturate. The authors could offer no explanation for the occurrence of minima in the *R*_H_ curves for specimens 2 and 3 and the apparent absence of a minimum in *R*_H_ for specimen 1 (intermediate purity); possibly this specimen has a minimum at *B*=0 T.

#### 2.4.2 Anisotropy Factor

**Fig. 17:**
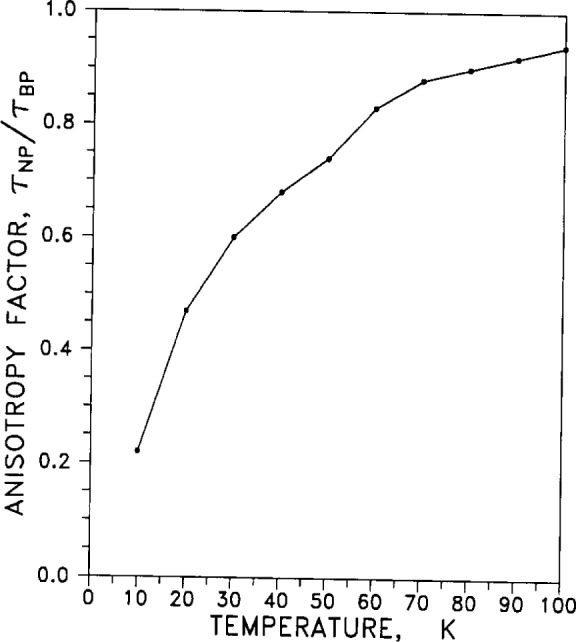
Anisotropy factor for electron-phonon scattering in “intrinsically pure” Ag, as a function of temperature. τ is the isotropic relaxation time; subscripts N and B refer to neck and belly of the Fermi surface, and *P* refers to electron-phonon scattering. [89]

The anisotropy factor τ_NP_/τ_BP_ is a measure of relaxation time τ on the “neck” (N) of the Fermi surface (FS) normalized by the relaxation time for the FS “belly” (B), for electron-phonon (P) scattering. Fig. 17 plots the anisotropy factor for pure silver.

### 2.5 Optical and High-Frequency Properties

#### 2.5.1 Spectral Emissivity

**Fig. 18:**
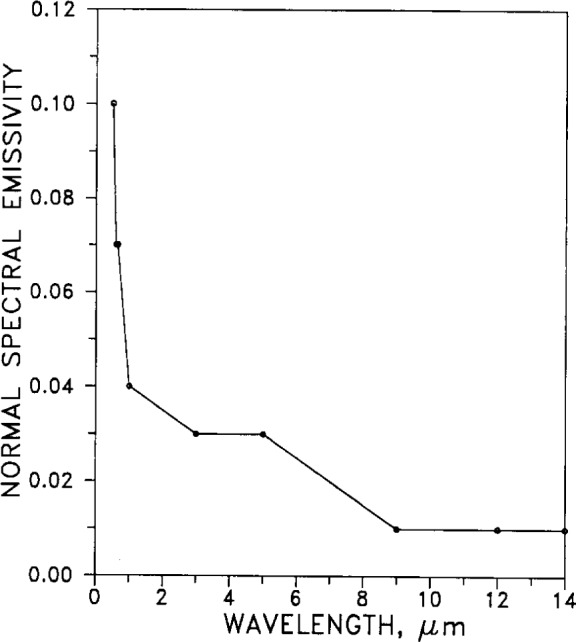
Normal spectral emissivity as a function of wavelength for a specimen at 295 K. [2]

Figure 18 shows how normal spectral emissivity of silver at 295 K depends on (visible and infrared) wavelength.

#### 2.5.2 Absorptance

**Fig. 19:**
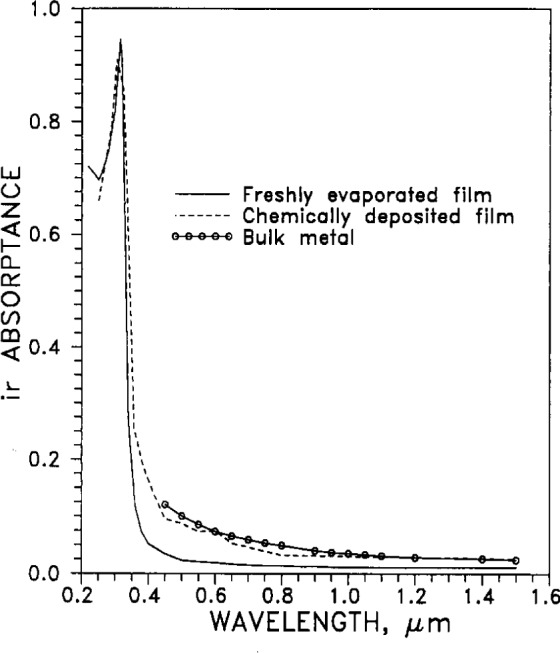
Infrared absorptance (1–reflectance) as a function of wavelength, for (a) freshly vacuum-evaporated film, (b) chemically deposited film, and (c) bulk metal. [2]

The absorptance of electromagnetic radiation from the near ultraviolet (220 nm) through the visible (400 nm to 700 nm), to the near infrared (5000 nm), is tabulated in Table 5 and plotted in Fig. 19. Values are given for freshly evaporated film, for bulk metal, and for a polished, chemically deposited surface. The latter two sets are probably more realistic for modeling the optical properties of silver-sheathed superconducting wire in practical applications.

#### 2.5.3 Angular Reflectivity

**Fig. 20:**
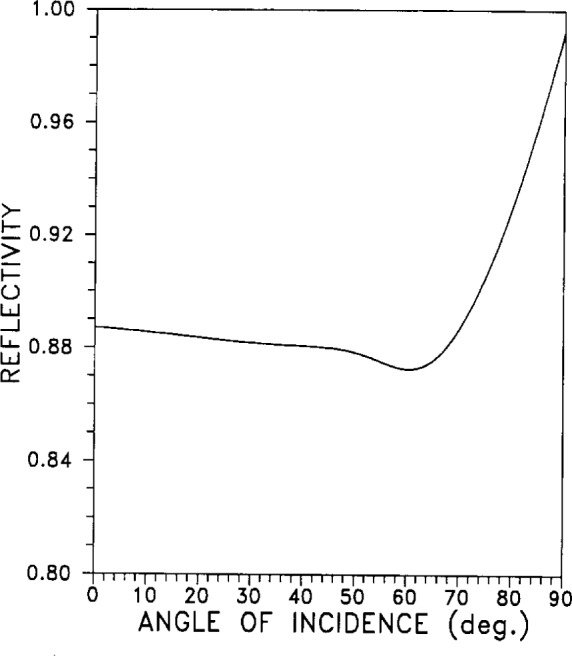
Angular reflectivity, at λ = 546 nm, as a function of angle of incidence. [143]

The angular reflectivity of silver, for visible light of wavelength *λ* =546 nm, is given in Fig. 20 as a function of angle of incidence.

#### 2.5.4 Index of Refraction

**Fig. 21:**
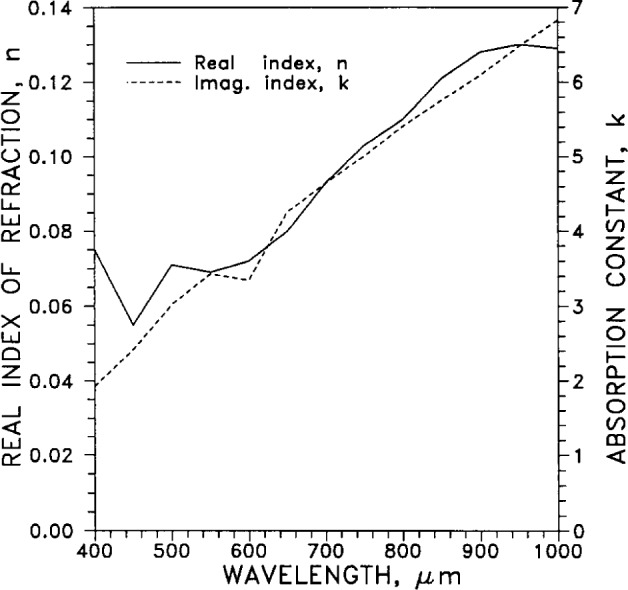
Index of refraction *n* and absorption constant *k* as functions of wavelength, *n* is the (real) index of refraction, and *k* is the absorption constant (imaginary index); the complex index *N = n−ik.* [2]

Table 6 lists the real and imaginary components, *n* and *k*, of the complex optical index of refraction *N*=*n*−*ik*, along with the reflectance *R*, for the visible and near infrared parts of the spectrum. The values are also plotted in Fig. 21. Optical reflectance plus absorptance for a metal sum to unity. It follows that surface films of oil, grease or dust, compromising the cleanliness of the silver and increasing its optical absorptance, easily reduce its reflectance.

### 2.6 Thermodynamic Properties

#### 2.6.1 Density

**Fig. 22:**
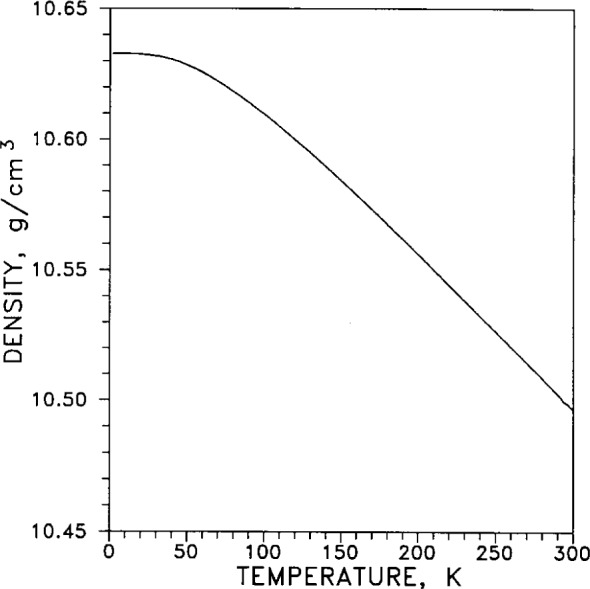
Density as a function of temperature. Density was calculated from the linear thermal expansion function and the density (10.492 g/cm^3^) at 300 K. [181]

From the temperature dependence of the linear thermal expansion coefficient α, plotted in Fig. 11, the volumetric expansion coefficient can be calculated, and then the dependence of density *ρ* with temperature. The results of this calculation is given in Fig. 22; a value of 10.492 g/cm^3^ (see Table 1) was assumed for the value of density at 20 °C.

#### 2.6.2 Specific Heat, Entropy, Enthalpy, and Gibbs Energy

**Fig. 23:**
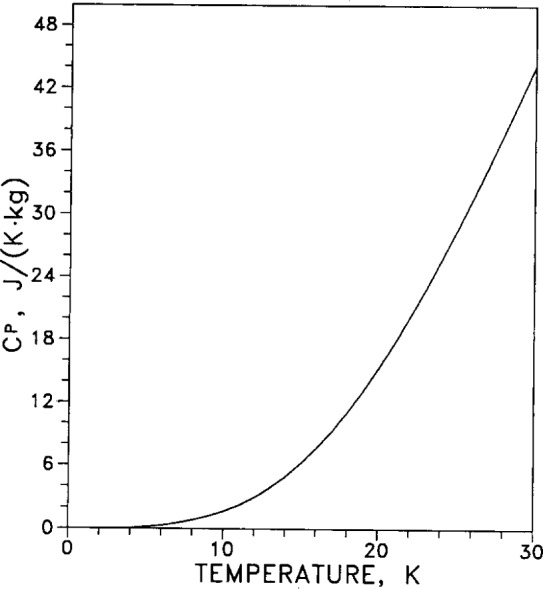
Low-temperature specific heat *C_p_* as a function (linear plot) of temperature for 0 K < *T* < 30 K. [191]

**Fig. 24:**
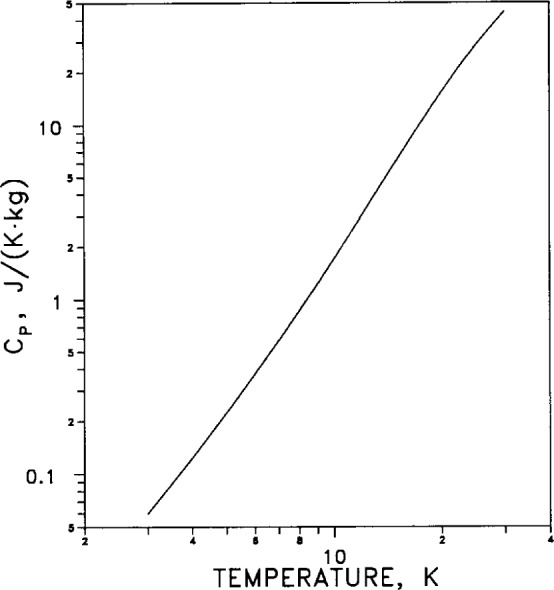
Low-temperature specific heat *C_p_* as a function (log-log plot) of temperature for 0 K < *T* < 30 K, emphasizing the low-temperature behavior. [191]

For the low-temperature range (temperatures from 3 K to 30 K) the temperature dependence of specific heat *C_p_* is tabulated in Table 7. The low-temperature specific heat is graphed in Fig. 23 on a linear scale to show the high-temperature behavior, and in Fig. 24 on a log-log scale to emphasize the low-temperature behavior.

Table 8 gives the temperature dependence over the range of temperatures from 0 K to 300 K for specific heat *C_p_*, entropy *S*, enthalpy *H*°*_T_*, Gibbs energy *G*°*_T_*, enthalpy function (*H*°*_T_* −*H*°_0_)/*T*, and Gibbs energy function −(*G*°*_T_*−*H*°_0_)/*T*. The enthalpy function *H*°*_T_* is the enthalpy at 101 kPa (1 atm) and temperature *T*, and similarly for the Gibbs energy. The reference function *H*°_0_ is the enthalpy at 101 kPa and 0 K.

**Fig. 25:**
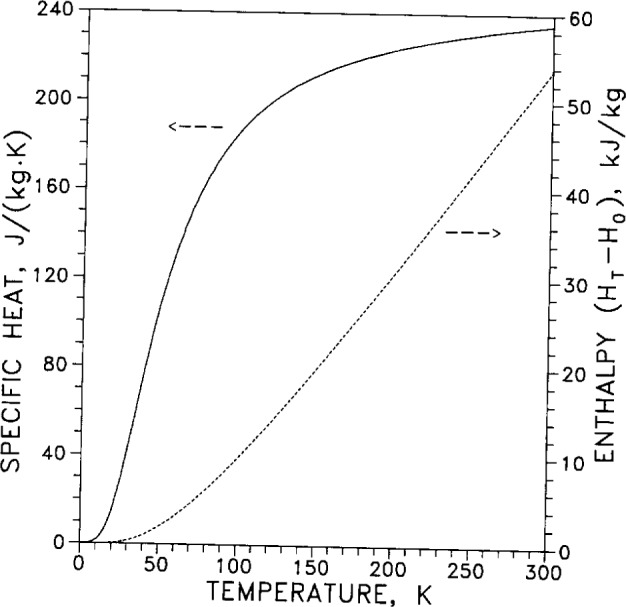
Specific heat *C_p_* (constant pressure) and enthalpy as functions of temperature. [183]

**Fig. 26:**
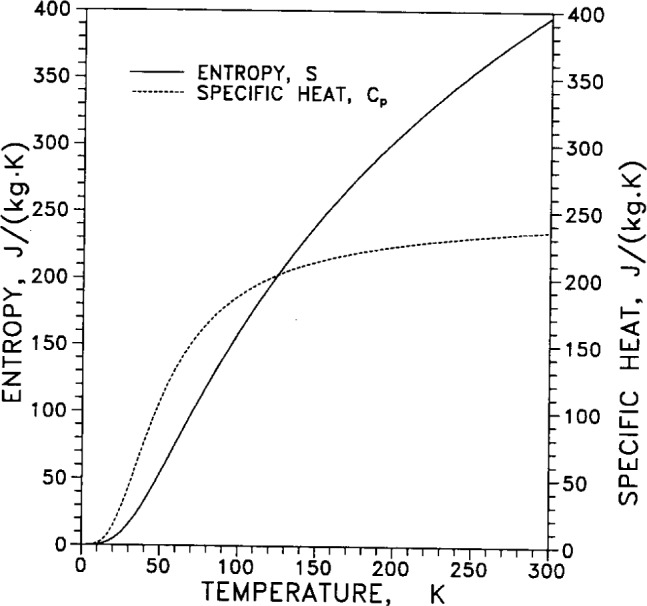
Entropy and specific heat as functions of temperature. [183]

- Specific heat capacity *C_p_* and enthalpy, *H*°*_T_*−*H*°_0_, are both plotted as functions of temperature in Fig. 25 for ease of comparison. The entropy *S* and specific heat *C_p_* are both plotted in Fig. 26 for ease of comparison. Entropy is the integral over temperature of the specific heat function divided by temperature. Consequently, as the specific heat approaches a constant value at high temperatures (law of Dulong and Petit) the entropy tends to increase linearly with temperature.

**Fig. 27:**
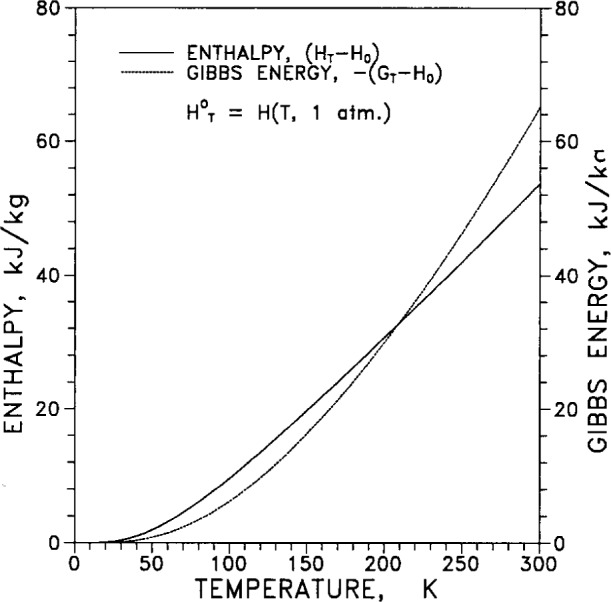
Enthalpy *H* and Gibbs energy G as functions of temperature. The reference function *H°_T_* is the enthalpy at 100 kPa (1 atm) and temperature *T.* [183]

Enthalpy (*H*°*_T_*−*H*°_0_) and Gibbs energy −(*G*°*_T_*−*H*°_0_) are plotted together in Fig. 27 as functions of temperature for ease of comparison.

**Fig. 28:**
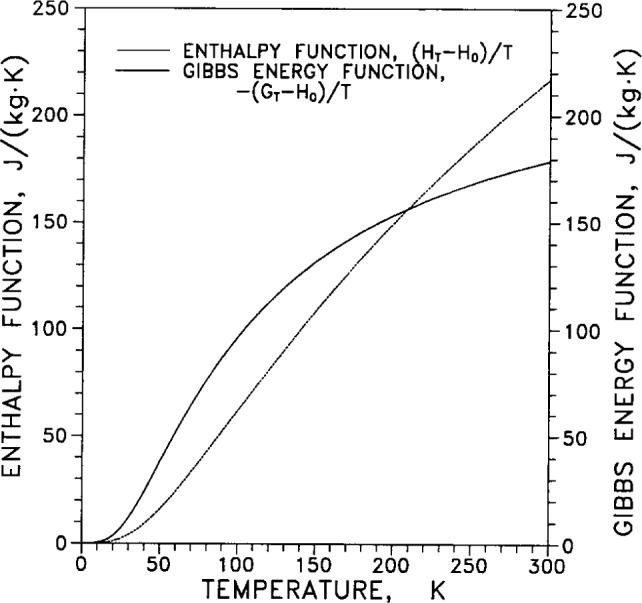
Enthalpy function (*H°_T_−H°*_0_)*/T* and Gibbs energy function *−(G°_T_−H°*_0_*)/T* as functions of temperature. The reference function *H°_T_* is the enthalpy at 100 kPa (1 atm) and temperature *T.* [183]

The enthalpy function (*H*°*_T_*−*H*°_0_)/*T* and Gibbs energy function −(*G*°*_T_*−*H*°_0_)/T are plotted in Fig. 28 as functions of temperature. These functions are sometimes useful to know because of the thermodynamic identity between Gibbs energy *G*, enthalpy *H* and entropy *S*: *G*−*H*= −*TS*. From this identity it follows that −(*G*−*H*)/*T*=*S*, and then −(*G*°*_T_*−*H*°_0_)/*T*+(*H*°*_T_*−*H*°_0_)/*T*=*S* (the superscripts “°” denote the function at 1 nominal atmosphere of pressure; the subscripts denote the temperature). The reference function *H*°_0_ is the enthalpy at 101 kPa and 0 K.

**Fig. 29:**
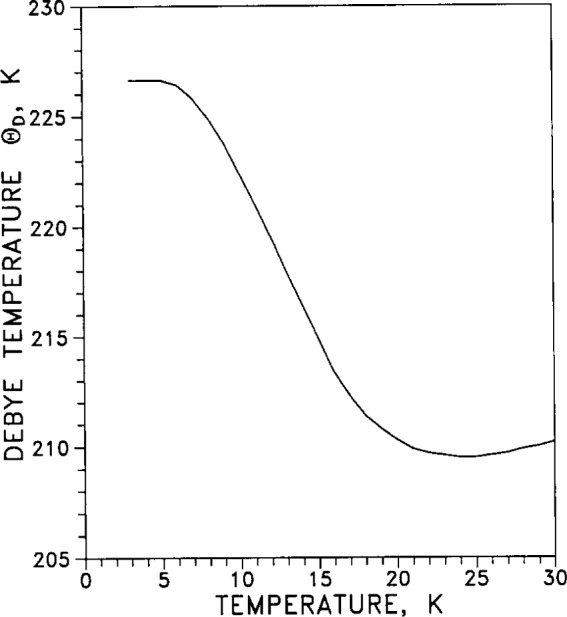
Low-temperature Debye temperature (calorimetric) as a function of temperature for 0 K < *T* < 30 K. [191]

**Fig. 30:**
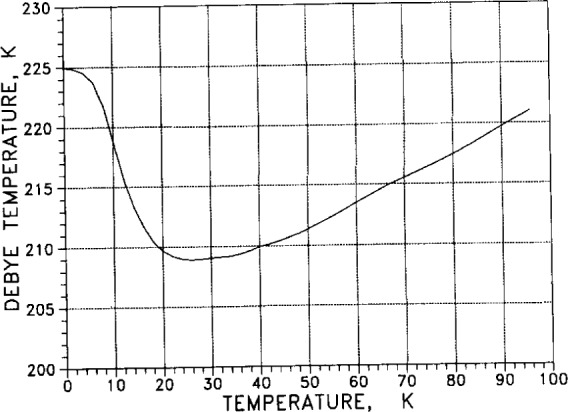
Debye temperature as a function of temperature for 0 K < *T* < 100 K. For this specimen the electronic specific heat *γ* was 0.66 mJ/(mol·K^2^) and the effective mass m*/m was 0.95. [193]

**Fig. 31:**
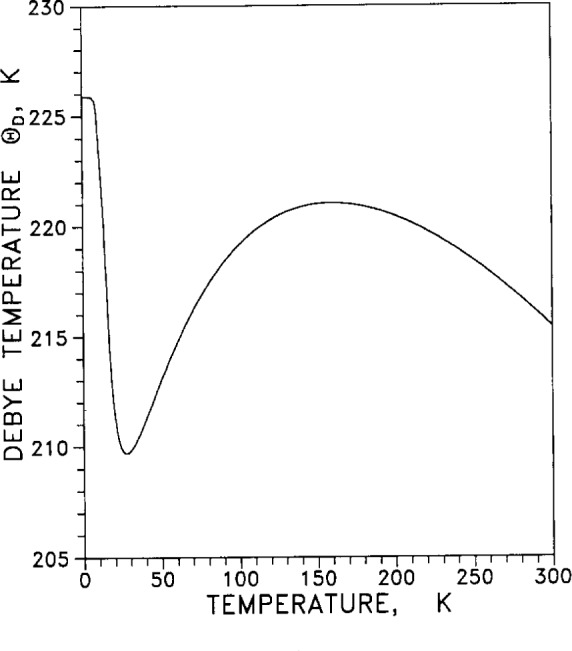
Debye temperature as a function of temperature for 0 K < *T* < 300 K. For this specimen the electronic specific heat *γ* was 0.65 mJ/(mol·K^2^). [183]

From the specific heat (calorimetric) measurements of Table 7, the low-temperature Debye characteristic temperature Θ_cal_= Θ_D_ as a function of temperature was also obtained, and values are listed in Table 7; the values are plotted in Fig. 29 for temperatures 0 K < *T* < 30 K. From a different set of specific heat measurements, Debye characteristic temperatures were obtained and plotted in Fig. 30 for temperatures 0 K< *T* < 100 K. From the specific heat measurements of Table 8, values for the Debye characteristic temperature Θ_D_ were obtained and are plotted in Fig. 31 for temperatures 0 K < *T* < 300 K. These three different plots of Θ_D_ cover somewhat different, but equally useful, ranges of temperature.

### 2.7 Mechanical Properties

#### 2.7.1 Hardness, Tensile Strength

**Fig. 32:**
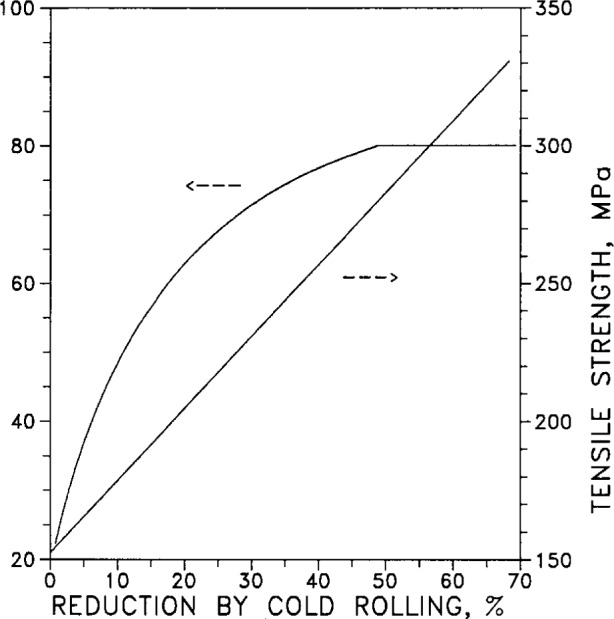
Rockwell F hardness and tensile strength as functions of percent reduction by cold rolling. [124]

Figure 32 displays Rockwell *F* hardness and tensile strength for the same specimens of pure silver, subjected to varying degrees of reduction by cold rolling at room temperature.

#### 2.7.2 Ultimate Tensile Strength

**Fig. 33:**
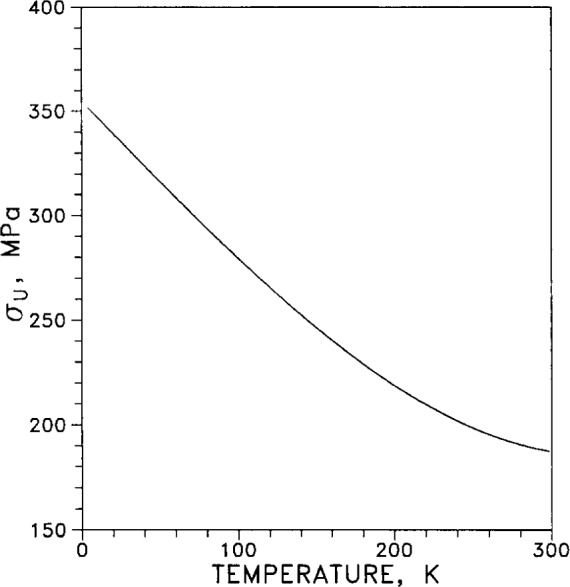
Ultimate tensile strength *σ_u_* as a function of temperature for a “99.995 % pure” specimen, annealed in Ar at 620 °C. [140]

**Fig. 34:**
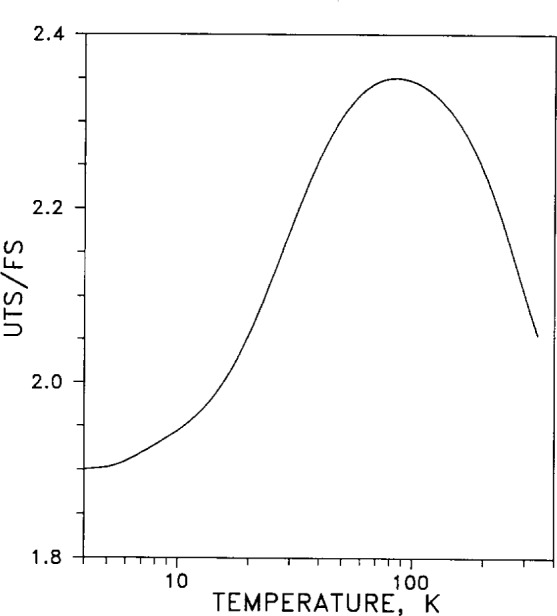
Ultimate tensile strength (*UTS*), normalized by fatigue strength (*FS*: peak stress for fracture in 10^5^ cycles), as a function of temperature. [140]

Ultimate tensile strength σ_U_ of annealed silver is plotted in Fig. 33 for temperatures from 0 K to 300 K. The effects of duration and temperature of annealing were not explored in this work (applies also to Figs. 34 and 44).

Ultimate tensile strength (*UTS*) is plotted in Fig. 34 as a function of temperature. The *UTS* has been normalized by dividing it by the fatigue strength, defined as the peak stress for fracture in 10^5^ cycles. The fatigue strength rises to a peak as the temperature is lowered below room temperature to about liquid-nitrogen temperature (78 K), but then falls off at temperatures below 78 K. The effects of duration and temperature of annealing were not explored in this work (applies also to Figs. 33 and 44).

#### 2.7.3 Tensile Stress vs Strain

**Fig. 35:**
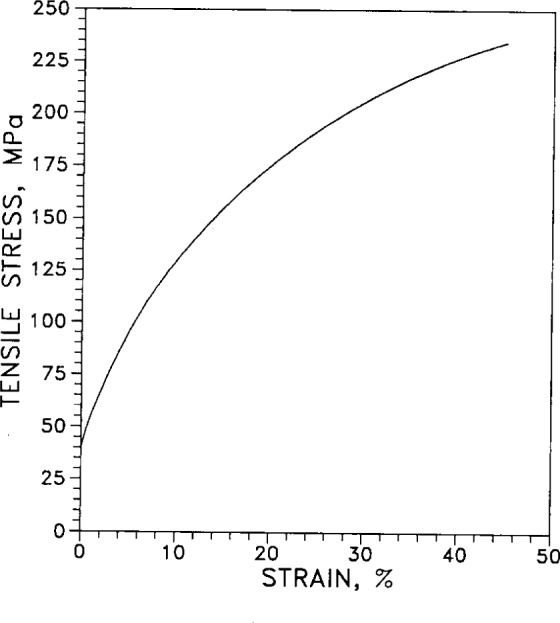
Tensile stress as a function of strain for cold-worked polycrystalline silver, “99.99% pure;” the grain size was 20 μm. [27]

Figure 35 gives tensile stress as a function of percent strain for cold-worked polycrystalline silver. The apparent intercept of the curve with the stress axis at 40 MPa is only an artifact; the original figure as published was quite small and the curve could not be resolved from the axis below this value.

**Fig. 36:**
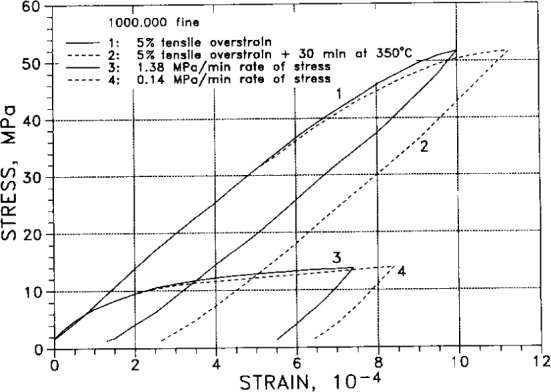
Stress as a function of strain for different values of stress rate and overstrain, for “1000.000 fine” silver. [132]

Figure 36 displays stress as a function of strain for different values of stress rate and overstrain, all for the same same specimen. The source paper gives no information on the rate of stress for curves 1 and 2. Tables V and VI of the original paper give the following information: for curve 1, the limit of proportionality was 17.9 MPa (1.3 ton/in^2^), the 0.01 % proof stress was 45.5 MPa (3.3 ton/in^2^), the elastic modulus was 69.7 GPa (10.1 Mpsi), the maximum applied stress was 51.9 MPa (3.76 ton/in^2^), the strain at maximum stress was 10.0×10^−4^, and the permanent strain was 1.34×10^−4^. For curve 4: the maximum applied stress was 13.8 MPa (1 ton/in^2^), the strain at maximum stress was 8.42×10^−4^, and the permanent strain was 6.4×10^−4^.

#### 2.7.4 Elastic Moduli

Adiabatic elastic coefficients, or moduli, (commonly termed “constants” even though they vary with temperature and pressure) are listed in Table 9. Of the six quantities listed in the table, only three are independent, and these are usually taken to be *C*_11_, *C*_12_ and one adiabatic elastic shear modulus, *C*_44_. Another elastic shear modulus is defined as *C*′=(*C*_11_−*C*_12_)/2, the bulk modulus is *B*=(*C*_11_+2*C*_12_)/3, and the longitudinal elastic modulus is *C*_L_ = (*C*_11_+*C*_12_+*C*_44_)/2. The elastic anisotropy is defined as *C*_44_/*C*′=2*C*_44_/(*C*_11_−*C*_12_).

**Fig. 37:**
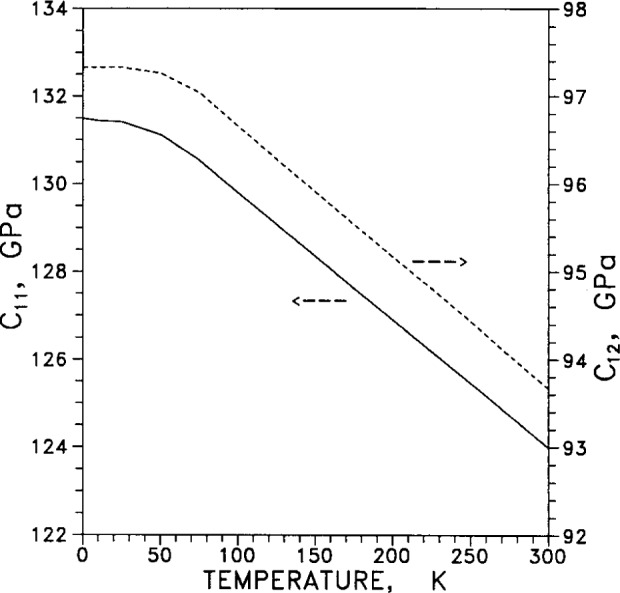
Elastic stiffness moduli, *C*_11_ and *C*_12_, as functions of temperature. [36]

**Fig. 38:**
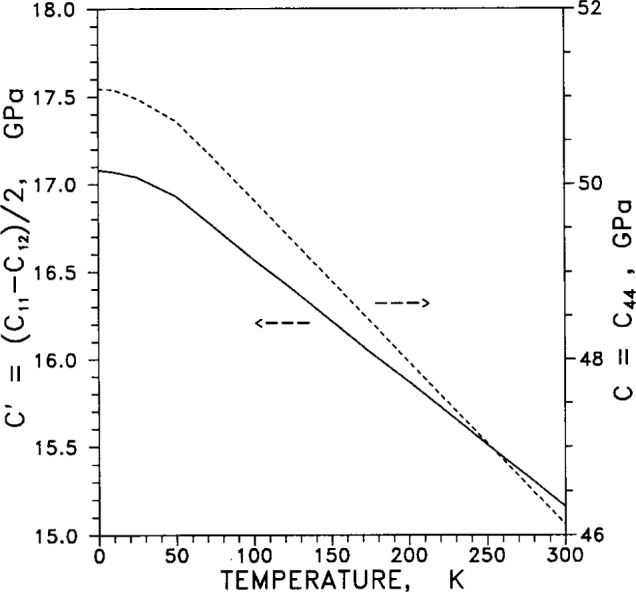
Adiabatic elastic shear moduli, *C* and *C*′, as functions of temperature. [36]

**Fig. 39:**
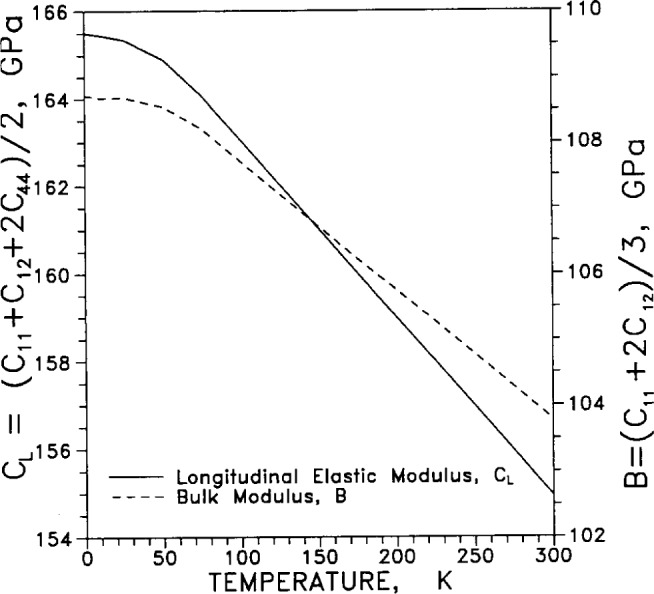
Bulk modulus *B* and longitudinal elastic modulus *C*_L_ as functions of temperature. [36]

- The two elastic stiffness moduli *C*_11_ and *C*_12_ are plotted in Fig. 37 as functions of temperature (the changes in slope at low temperature are artifacts of the finite number of data points). Fig. 38 displays two adiabatic elastic shear moduli, *C*=*C*_44_ and *C*′=(*C*_11_′*C*_12_)/2, plotted together as functions of temperature. The bulk modulus *B* and the longitudinal elastic modulus *C*_L_ are plotted together in Fig. 39 as functions of temperature. All the elastic moduli are extremal at liquid-helium temperatures.

**Fig. 40:**
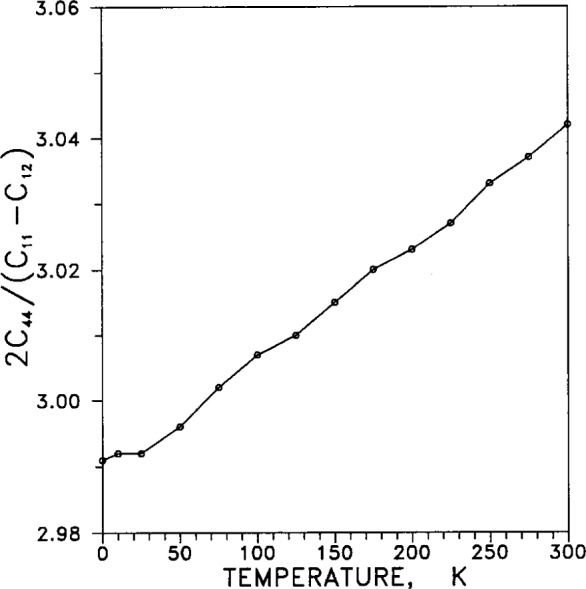
Elastic anisotropy coefficient, 2*C*_44_/(*C*_11_−*C*_12_) as a function of temperature. [36]

Figure 40 shows the elastic anisotropy coefficient 2*C*_44_/(*C*_11_−*C*_12_) as a function of temperature. It is minimal at 0 K.

#### 2.7.5 Young’s Modulus

**Fig. 41:**
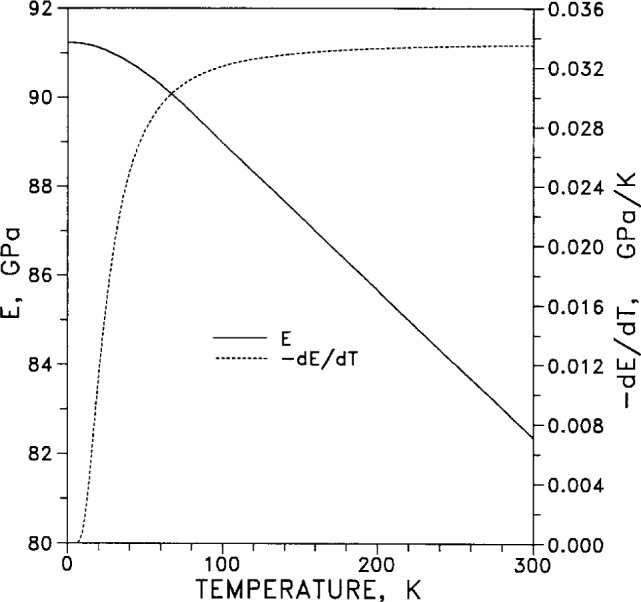
Young’s modulus *E* and its temperature derivative d*E*/d*T* as functions of temperature. [134]

Young’s modulus *E* and its temperature derivative d*E*/d*T* are plotted in Fig. 41 as functions of temperature. The values for *E* and d*E*/d*T* were calculated from elastic-coefficient data.

#### 2.7.6 Creep

**Fig. 42:**
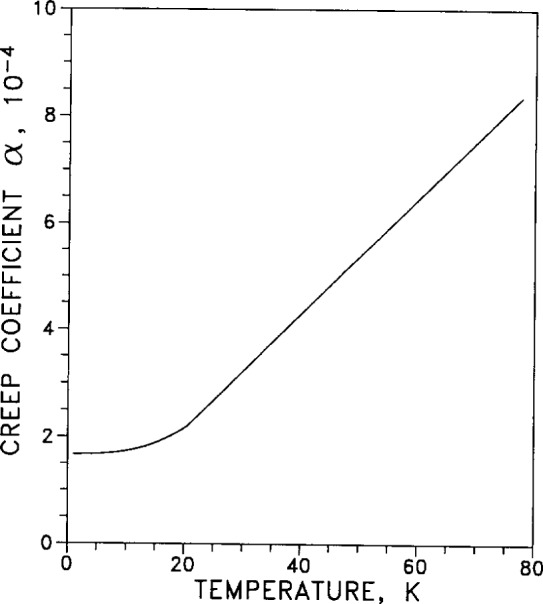
Low-temperature creep as a function of temperature. Strain ϵ = α·ln(γ*t*+l), where *α* is the creep coefficient and *γ* is the time proportionality constant. Specimen was a single crystal in “easy-glide stage,” at a deformation stress of 5.9 MPa. [128]

The creep coefficient *α* is plotted in Fig. 42 as a function of temperature for an applied stress of 5.9 MPa. The creep strain is given by the expression *∊* =*α*·ln(*γt*+1), where *γ* is a “time proportionality constant” and *t* is time. Values for *γ* were not given in the source paper. In Fig. 1 of the original paper (Sec. 3.1, Ref. [128]) creep is plotted as functions of both time and log(time), but corresponding plots are not related by just a single mapping (linear to logarithmic). Careful study raises many unanswered questions, so the reader is cautioned to be careful in using the information from Fig. 1 of Ref. [128].

#### 2.7.7 Flow Stress

**Fig. 43:**
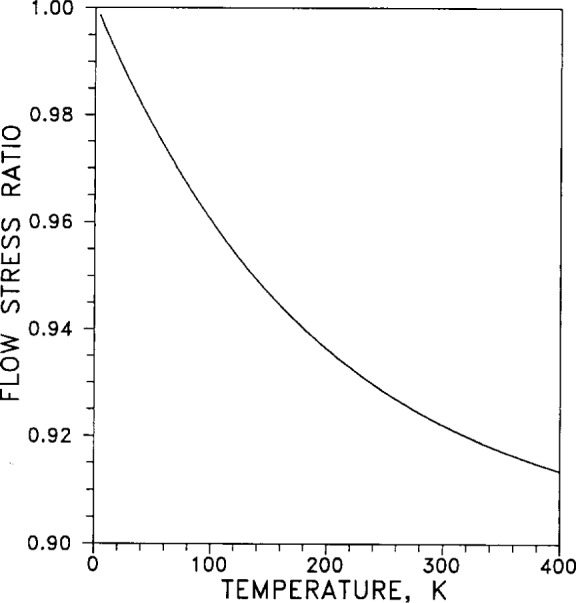
Ratio of flow stress τ to shear modulus *G* (ratio normalized to unity at *T* =*0*): (τ*_T_*/G*_T_*)/(τ_0_/G_0_), as a function of temperature *T.* [134]

The ratio τ*_T_*/*G_T_* of flow stress *τ* to shear modulus *G*, normalized to unity at *T*=0, (*τ_T_*/*G_T_*)/(*τ*_0_/*G*_0_), is plotted in Fig. 43 as a function of temperature. The subscripts *T* denote temperature dependence.

#### 2.7.8 Fatigue

**Fig. 44:**
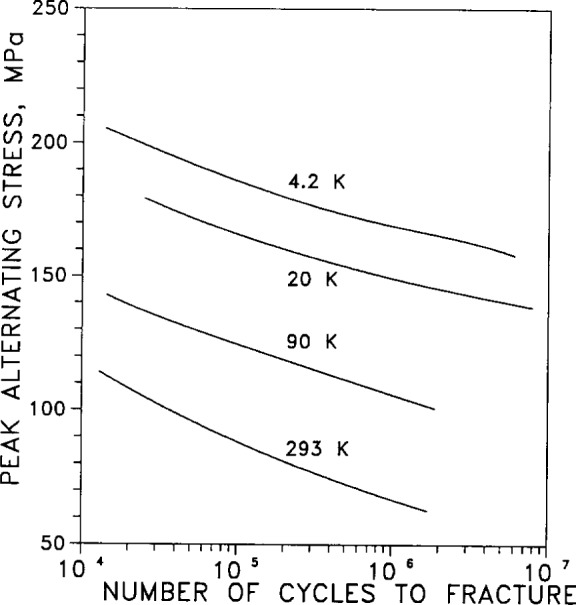
Tension-compression fatigue: peak alternating stress as a function of number of cycles to fracture, for temperatures of 4.2 K, 20 K, 90 K, and 293 K. [140]

Tension-compression fatigue is plotted in Fig. 44 as a function of number of cycles to fracture for test temperatures of 4.2 K, 20 K, 90 K, and 293 K. The effects of temperature and duration of annealing were not explored in this work (applies also to Figs. 33 and 34).

#### 2.7.9 Yield Strength

**Fig. 45:**
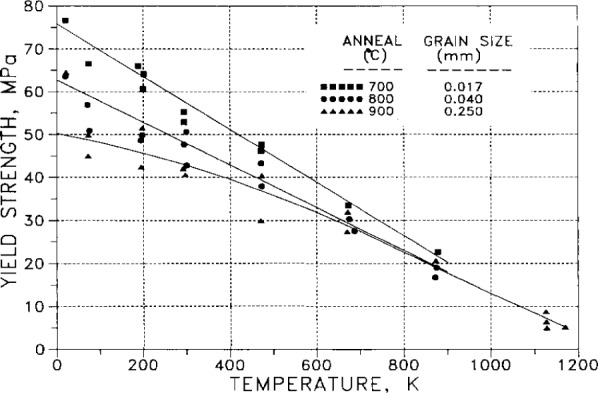
Yield strength as a function of temperature for three different annealing temperatures and concomitant grain sizes. [120]

Yield strength (defined as stress at 0.005 strain) is plotted as a function of temperature for specimens of high-purity (0.9997+) silver in Fig. 45. The specimens, of three different grain sizes (0.017 mm, 0.040 mm, and 0.250 mm), had been annealed at temperatures of 700 °C, 800 °C, and 900 °C, respectively.

**Fig. 46:**
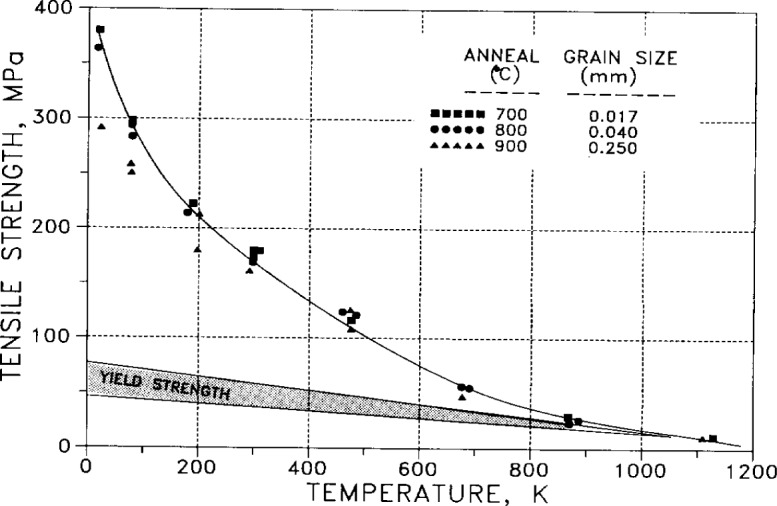
Tensile strength as a function of temperature (for the same annealing temperatures and grain sizes as in Fig. 45). [120]

In Fig. 46, tensile strength is plotted as a function of temperature and compared to the yield strength, for the same three grain sizes (related to annealing conditions) of Fig. 45.

#### 2.7.10 Velocity of Dislocations

**Fig. 47:**
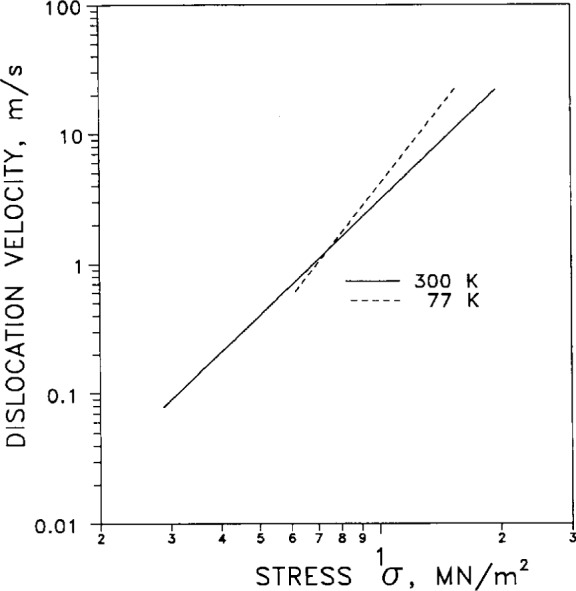
Velocity of dislocations moving in a < 110 > {111 } glide system, at temperatures of 300 K and 77 K, as a function of stress *σ.* [28]

Fig. 47 shows the velocities of dislocations moving in a <110>{111} glide system as a function of stress *σ*, for specimen temperatures of 77 K and 300 K. There is a crossover at a stress of about 7 MN/m^2^; above this stress the dislocation velocity is greater at 77 K, but below this crossover stress the velocity is greater at 300 K.

#### 2.7.11 Displacement Cross Section

**Fig. 48:**
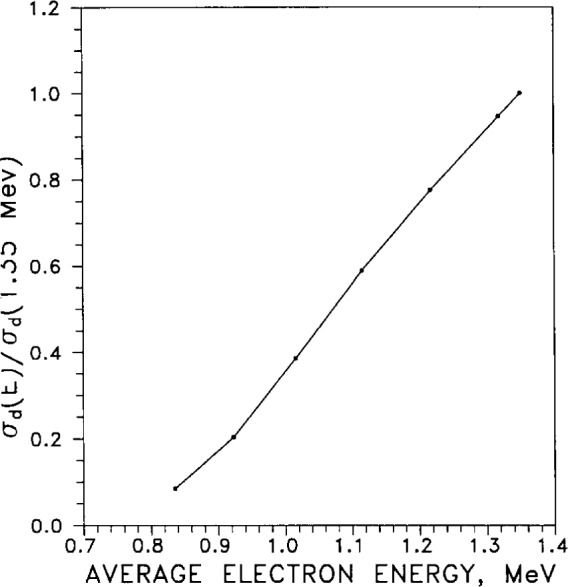
Displacement cross section *σ*_d_ as a function of electron energy required to displace an Ag atom from a lattice site, normalized to the cross-section value at 1.35 MeV. The displacement threshold energy was 28 eV. [25]

The displacement cross section as a function of electron energy required to displace a silver atom from a lattice site (in bulk silver) is graphed in Fig. 48. The cross sections were normalized by dividing by the displacement cross section at 1.35 MeV, the largest electron energy used. The associated threshold energy for displacement was 28 eV.

### 2.8 Miscellaneous

For the convenience of those working with cryogenic fluids, some useful thermodynamic properties are listed in Table 10. For commonly used cryogenic liquids at normal boiling points and for vapors at 101 kPa (1 atm), density, enthalpy, and specific heat are tabulated.

## 3. Bibliography

**Table:** 

Order of Topics		Ref. Nos.
3.1	General Reviews .........................	1–18
3.2	Band Structure ...........................	19–21
	3.2.1 de Haas-van Alphen Effect ...........	22
	3.2.2 Fermi Surface .....................	23–24
3.3	Defects .................................	25–26
	3.3.1 Dislocations .......................	27–30
3.4	Diffusion ...............................	31–32
3.5	Elastic Moduli ...........................	33–37
	3.5.1 Pressure Dependence................	38–39
	3.5.2 Young’s Modulus ...................	40
3.6	Electrical Resistivity ......................	41–77
	3.6.1 Change with Deformation ............	78–83
	3.6.2 Damage by Irradiation...............	84–87
	3.6.3 Hall Coefficient ....................	88–90
	3.6.4 Lorenz Number ....................	91–92
	3.6.5 Magnetoresistance ..................	93–104
	3.6.6 Matthiessen’s Rule..................	105–110
	3.6.7 Pressure Dependence................	111–114
3.7	Magnetic Susceptibility ....................	115–117
3.8	Mechanical Properties .....................	118–138
	3.8.1 Fatigue ...........................	139–140
	3.8.2 Tensile Strength ....................	141
3.9	Optical And High-frequency Properties .......	142–150
	3.9.1 Skin Effect ........................	151–153
3.10	Proximity Effect (Superconductive) ..........	154
3.11	Thermal Conductivity .....................	155–171
	3.11.1 Magnetothermal Resistance..........	172–173
	3.11.2 Thermal Contact Resistance .........	174
	3.11.3 Thermal Diffusivity................	175
3.12	Thermal Expansion .......................	176–182
3.13	Thermodynamic Properties .................	183–186
	3.13.1 Debye Temperature ................	187–188
	3.13.2 Specific Heat .....................	189–194
3.14	Thermoelectric Properties ..................	195–205
3.15	Transport Properties ......................	206–212
	3.15.1 Nernst-Ettinghausen Effect ..........	213
3.16	Gas Solubility ...........................	214
3.17	Coin Silver ..............................	215

### 3.1 General Reviews

1. Butts, Allison, and Coxe, Charles D., Silver: Economics, Metallurgy and Use, Robert E. Krieger, Malabar FL (1967).
  Reviews history, sources and markets; extractive and refining processes; physical, chemical, mechanical properties (including bearings and electrical contacts) and behavior of silver bimetals and alloys; metallography of silver; silver batteries, brazing, catalysts, electroplating, and mirrors; silver in dentistry, medicine and photography; fabrication of articles (flatware, holloware) from silver and alloys; powder metallurgy, and migration of silver.
2. American Institute of Physics Handbook, Gray, Dwight E., Coordinating Editor, McGraw-Hill, NY (1957).
†18 †19 †21 †T5 †T6Sections on mechanics, acoustics, heat, electricity and magnetism, optics, atomic and molecular physics, and nuclear physics summarize physical properties of materials (for the vast majority of cases, by tabulation).
3. American Society for Metals, Metals Handbook Ninth Edition: Volume 2: Properties and Selection: Nonferrous Alloys and Pure Metals, Coxe, C. D., McDonald, A. S., and Sistare, G. H., Jr., compilers: Properties of Silver and Silver Alloys, pp. 671–676; Carapella, S. C., Jr., and Corrigan, D. A., compilers: Pure Metals: Silver, pp. 794–96, ASM, Metals Park, OH (1979).
  Tensile strength and elongation (assume room temperature) are plotted as a function of amount of cold work (0 % to 74 %) and as a function of annealing temperature (100 °C to 700 °C).
4. Bell, J. F., The Experimental Foundations of Solid Mechanics, Encyclopedia of Physics, Vol. VIa/1: Mechanics of Solids I, C. Truesdell, ed., pp. 1–801, Springer, Berlin (1973).
  The historical and experimental foundations of solid mechanics for metals, plastics and elastic materials are reviewed.
5. Boyer, H. E., and Gall, T. L., eds., Metals Handbook, Desk Edition, ASM, Metals Park, OH (1986).
  General physical and structural properties of the metallic elements are tabulated, along with information on processing, testing and inspection.
6. Corruccini, R. J., Properties of Materials at Low Temperatures, Chem. Eng. Prog. (Part 1): **53**(6), 262–7; (Part 2): **53**(7), 342–6 and (Part 3): **53**(8), 397–402 (1957).
  This is a practical review of the elementary theories of specific heat, thermal expansion, and electrical and thermal conductivities, and of the properties of metals, alloys, and insulators at low temperatures.
7. Kittel, C., Tables, Introd. to Solid State Physics, 6th Ed., J. Wiley (1986).
  For elements (arranged in periodic tables): this university text tabulates cohesive energies at 0 K and 298.15 K, melting points, ionization energies, crystal structures; densities, atomic concentrations and nearest-neighbor distances; atomic and ionic radii; isothermal bulk moduli and compressibilities at room temperature; adiabatic elastic stiffness constants of cubic crystals at low temperatures and room temperature; low-temperature limits of the Debye temperatures; linear thermal expansion coefficients; calculated free-electron Fermi-surface parameters for metals (mostly room-temperature values); experimental and free-electron values of electronic specific heat constants *γ* of metals; electrical conductivity and resistivity; experimental Lorenz numbers; comparisons of observed with calculated (free-electron theory) Hall constants; superconducting transition temperatures, nuclear-magnetic-resonance (NMR) data, diffusion constants and activation energies, and electron work functions. Many other tables are given for a limited selection of atoms or compounds.
8. Klemens, P. G., Thermal Conductivity of Solids at Low Temperatures, Encyclopedia of Physics, Vol. XIV: Low Temperature Physics I, Springer, Berlin (1956) pp. 198–279.
  The theories of thermal conductivity of dielectric solids, of metals and alloys (both electronic and lattice components), and of superconductors, are reviewed.
9. MacDonald, D. K. C., Electrical Conductivity of Metals and Alloys at Low Temperatures, Encyclopedia of Physics, Vol. XIV: Low Temperature Physics I, Springer, Berlin (1956) pp. 137–197.
  The experimental techniques for measuring electrical conductivity are reviewed and existing data are compared with theory.
10. Meaden, G. T., Electrical Resistance of Metals, Plenum, New York (1965).
  This book tabulates experimental data for the resistivity for temperatures from 20 K to 295 K and summarizes measurement methods. It discusses the theory of resistivity and the effects of deformation, irradiation, pressure, and magnetic field, as well as size effects, on resistivity.
11. Pollock, Daniel D., Physical Properties of Materials for Engineers: Vol. II, CRC Press, Boca Raton (FL).
  A table is given of change in electrical resistivity per “atomic percent” (mole fraction) of alloying element in Ag, Au and Cu; the data are from J. O. Linde (Ref. 65).
12. Powell, R. L., Thermophysical Behavior of Metals at Cryogenic Temperatures, pp. 134–148, ASTM STP 387, Behavior of Materials at Cryogenic Temperatures, ASTM, PA (1965).
  Thermophysical properties are tabulated and graphed.
13. Reed, Richard P., and Clark, Alan F., Materials at Low Temperatures, American Society for Metals, Metals Park, OH (1983).
  Topical chapters review elastic, electrical, magnetic, mechanical, metallurgical, thermodynamic, thermal, and transport properties of metals, alloys, superconductors, and composites useful for applications at cryogenic temperatures.
14. Robertson, A. R., Precious Metals: Precious Metals and Their Uses, Metals Handbook, Vol. 2, 10th Ed., ASM, Metals Park, OH (1990).
  Some physical, chemical and mechanical properties of Ag, Pt, Pd, Ir, Rh, Os, Ru, and Au are tabulated.
15. Rogers, B. A., Schoonover, I. C., and Jordan L., Silver: Its Properties and Industrial Uses, NBS Circular C412 (1936).
  This work comprehensively reviews physical (atomic, mechanical, thermal, electrical, galvanometric, thermomagnetic, thermoelectric, and optical; magnetic susceptibility), chemical (electrochemical, corrosion, and catalytic), and technological (mechanical) properties of pure silver, and some properties of binary alloys (Ag with Al, Cd, Cu, Pb, Sn, and Zn). Industrial uses of pure Ag as a bactericide, in chemical equipment resistant to corrosion, and in electrical equipment are briefly discussed.
16. Rosenberg, H. M., Low Temperature Solid State Physics: Some Selected Topics, Oxford Univ., London (1963).
  This text reviews theory of and experimental data for specific heat, transport properties, thermal expansion, and magnetic and mechanical properties, for metals, semiconductors and superconductors.
17. Rosenberg, H. M., The Properties of Metals at Low Temperatures, Chap. 5 of Prog. in Metal Physics, Vol. 7, B. Chalmers and R. King, eds., Pergamon, NY (1958).
  The thermal conductivity, electrical resistivity and superconductivity, and mechanical properties of metals in general, at temperatures below 90 K, are reviewed, with some properties of specific metals and alloys given.
18. Smithells Metals Reference Book, Sixth Edition, Eric A Brandes, Editor, Butterworths, London (1983).
  Many thermophysical properties are tabulated.

### 3.2 Band Structure

1. Christensen, N. E., The Band Structure of Silver and Optical Interband Transitions, phys. stat. sol. (b) **54**, 551–563 (1972).
  The band structure of silver was calculated using the relativistic augmented-plane-wave (RAPW) method. A plot of density of states versus energy from 0 Ryd to 1.5 Ryd (1 Ryd = 13.6 eV) is given. The Fermi energy is approximately 0.444 Ryd above the muffin-tin zero, or 0.551 Ryd above the band bottom (Γ_6_^+^). The electronic density of states per atom at the Fermi level, *N*(*E*_F_), is 3.4556 Ryd^−1^. The band structure has been checked against optical experiments and overall agreement is very good.
2. Mathewson, A. G., Aronsson, H., and Bernland, L. G., The conduction electron optical mass in pure silver; J. Phys. F: Metal Phys. 2, L39–L41 (1972).
  A new, lower value for the optical mass *m*_0_ of conduction electrons in pure silver is reported. The value *m*_0_ = 0.85 *m*_e_ was obtained for silver evaporated onto a sapphire substrate in an ultra-high vacuum of 6.7×10^−8^ Pa (5×10^−10^ Torr). After exposure of the silver film to air for 16 hours the optical mass was found to have increased to 0.87. The increase is attributed to the development of a film of silver sulfide on the surface of the silver.
3. Sondheimer, E. H., The Mean Free Path of Electrons in Metals, Adv. Phys. **1**(1), 1–42 (1952).
  The theory of electrons in metals is reviewed. Electrical conduction and magnetic (Hall) effects in thin films and wires are discussed and compared with experimental results for Na wires. The anomalous skin effect is treated and the experimental high-frequency surface resistance of silver at low temperatures is graphed, as well as the theoretical absorption coefficient of silver at 4.2 K.

#### 3.2.1 de-Haas-van Alphen Effect

1. Coleridge, P. T., and Templeton, I. M., High precision de Haas-van Alphen measurements in the noble metals, J. Phys. F: Metal Phys. **2**, 643–656 (1972).
  The de Haas-van Alphen frequencies for the symmetry-direction orbits (<111> belly, <111> neck, <100> belly, <100> rosette, and <110> dogs-bone) and the turning point of the (110) plane-belly turning point were measured for Ag, Au, and Cu. The data are estimated to have an “absolute precision” of 20 ppm (parts in 10^6^) and a “relative precision” of nearly 1 ppm. Comparisons with other measurements from the literature are tabulated.

#### 3.2.2 Fermi Surface

1. Cracknell, A. P., The Fermi Surfaces of Metals: A Description of the Fermi Surfaces of the Metallic Elements, Barnes & Noble, NY (1971).
  The neck radius of the Fermi surface of silver is 0.14 (with the radius of the spherical free-electron Fermi surface as the unit), and is smaller than the radii of the Fermi surfaces of both Cu and Au.
2. Visscher, P. B., and Falicov, L. M., Review Article: Fermi Surface Properties of Metals, Phys. Stat. Sol. (b) **54**, 9–51 (1972).
  The independent-particle picture for electrons, simple equilibrium phenomena, dynamics of electrons in electric and magnetic fields, equilibrium phenomena in magnetic fields, simple transport phenomena, and transport phenomena in magnetic fields are reviewed in separate sections. A value of *γ* = 0.646 mJ/(K^2^·mol) for silver is given. Table 4 is a comprehensive list of references for band- structure calculations, the de Haas-van Alphen effect, the anomalous skin effect, galvanomagnetic effects, cyclotron resonance, size effects, and ultrasonics. There are 23 references for the above properties of silver.

### 3.3 Defects

Bass, Jack, Deviations from Matthiessen’s Rule, Adv. Phys. **21**, 431–604 (1972), see Ref. 106, Sec. **3.6.6 Matthiessen’s Rule.**

1. Damask, A. C., and Dienes, G. J., Point Defects in Metals, Gordon and Breach, NY (1963).
†48This work reviews the general theory of: (1) thermodynamics, energies, mobility, and production of defects; (2) theory of annealing; (3) analysis of annealing curves; (4) influence of point defects on physical properties; (5) experimental investigations of the effects of quenching, irradiation, annealing and diffusion. The energy of formation *E*_F_ of (monovacancy) defects is given as 1.09 eV. The change in electrical resistivity per electron flux (number of incident particles per cm^2^) is plotted as a function of energy of incident electrons, and then replotted as the displacement cross section for moving a silver atom from a lattice site by incident electrons, again as a function of energy of incident electrons. The threshold energy *E*_D_ for displacing a silver atom from its lattice site is given as 28 eV, and the resistivity change per unit of defect concentration in “atomic percent” (mole fraction) △*ρ*_F_ is 1.4 μΩ·cm/(^at^/_o_). The damage rate under fast-neutron irradiation at 20 °C at a flux of 7×10^11^ cm^2^·s^−1^ is 3.35×10^−11^ Ω·cm/h. The neutron exposure required to produce 1^at^/_°_ of vacancy-interstitial pairs is 1.05×10^20^
*nvt* of fast neutrons (*n* is number density of neutrons, *v* is velocity, *t* is time; *nvt* is neutron fluence, number per second per unit area).
  Broom, T., Lattice Defects and the Electrical Resistivity of Metals, Adv. Phys. **3**(9), 26–83 (1954), see Ref. 82, Sec. **3.6.1 Change with Deformation**.
  Jongenburger, P., The Extra-Resistivity Due to Vacancies in Copper, Silver and Gold, Appl. Sci. Res. **B3**, 237–248 (1953?), see Ref. 55, Sec. **3.6 Electrical Resistivity.**
2. Ramsteiner, F., Schuele, W., and Seeger, A., Untersuchung atomarer Fehlstellen in verformtem und abgeschrecktem Silber (An Investigation of Atomic Vacancies in Rolled and Quenched Silver), Phys. Stat. Sol. **2**, 1005–1020 (1962) (in German).

The annealing of quenched and cold-rolled silver (purities of 99.9 % and 99.999 %) was studied in the range of temperature from −40 °C to +250 °C. Distinct recovery processes were found. For quenched silver, a recovery stage attributed to migration of vacancies was found between 50 °C and 130 °C, with an activation energy of 0.88 eV. For cold-rolled silver, a recovery stage attributed to interstitial migration was found between −40 °C and −10 °C, with an activation energy of 0.60 eV. For the cold-rolled specimen, a recovery stage found above 70 °C was thought to be due to rearrangement and annihilation of dislocations.

#### 3.3.1 Dislocations

1. Bailey, J. E., and Hirsch, P. B., The Dislocation Distribution, Flow Stress, and Stored Energy in Cold-Worked Polycrystalline Silver, Phil. Mag. **5**, 485–497 (1960).
†35For cold-worked polycrystalline silver of “99.99 % purity,” plots are given for stress versus extension (strain). Dislocation densities in silver foil are tabulated for three different extensions. Total stored energy, and energies released during recovery and during recrystallization are tabulated for four different extensions.
  Barnard, R. D., Some Remarks on Deviations from Matthiessen’s Rule in Dilute Alloy Systems, Phys. Stat. Sol. (b) **104**, 613–620 (1981), see Ref. 105, Sec. **3.6.6 Matthiessen’s Rule.**
  Bergmann, A., Kaveh, M., and Wiser, N., Electrical resistivity of the noble metals at low temperatures: II. Effect of electron-dislocation scattering, J. Phys. F: Metal Phys. **12**, 3009–3030 (1982), see Ref. 47, Sec. **3.6 Electrical Resistivity.**
  Hashimoto, S., Miura, S., and Yagi, T., Decrease in Dislocation Density During Reverse Deformation in Silver Single Crystals by Etch-Pit Method, Scripta Metall. **10**(9), 823–27 (1976), see Ref. 139, Sec. **3.8.1 Fatigue.**
  McLean, D., Strain Hardening in Pure Metals, Chap. 5 of Mechanical Properties of Metals, J. Wiley, NY (1962), see Ref. 131, Sec. 3.8. **Mechanical Properties.**
2. Nadgornyi, E., Dislocation Dynamics and Mechanical Properties of Crystals, Prog. Matls. Sci., Vol. 31, J. W. Christian, P. Haasen, and T. B. Massalski, eds., p. 319, Pergamon, Oxford (1988).
†47This work reviews dislocation dynamics theoretically and experimentally. The velocity of dislocations moving along a <110>{111} glide system at 300 K and 77 K is plotted (Fig. 6.21), for silver and dilute silver-based alloys (Ag with In, Sb, Sn). For the source of this particular plot see the paper by H. Suga and T. Imura (Ref. 29).
3. Suga, Hisaaki, and Imura, Toru, Dynamic Behav-ior of Dislocations in Silver and Silver Base Dilute Alloy Single Crystals, Japn. Inst. Metals **17**, 605–614 (1976).
  The density, distribution, velocity, multiplication and mobility of dislocations is studied by changing solute content and test temperature. The stress exponent *m* for the velocity of dislocations in pure silver is 2.8 at room temperature. Four plots of data for dislocation parameters are given.
4. Takamura, S., and Kobiyama, M., Dislocation Pinning in Al and Ag Alloys after Low-Temperature Deformation, Phys. Stat. Sol. (a) **95**, 165–172 (1986).
†6Specimens of Ag and Al (99.999 % pure), and of dilute alloys of each metal, were torsionally deformed at 77 K. The electrical resistivities *ρ*_0_ after isochronal annealing were measured at 4.2 K. In silver annealing stages were observed at 110 K and 230 K to 250 K. The lower-temperature stage is interpreted as being due to migration of interstitial clusters or vacancy-type defects near dislocations, and the higher-temperature stage, to long-range migration of vacancies and dissociation of vacancy clusters. The elastic modulus changes at 110 K and 240 K.

### 3.4 Diffusion

1. Burke, J. E., and Turnbull, D., Recrystallization and Grain Growth, Prog. Metal Phys. Vol. 3, B. Chalmers, ed., p. 247, Pergamon, London (1952).
  For silver, the free energy of activation for lattice self-diffusion, (Δ*F*_A_)_L_, is 163.2 kJ/mol; for grain-boundary diffusion, (Δ*F*_A_)_B_ is 77.8 kJ/mol; for grain-boundary self-diffusion, (Δ*F*_A_)_G_ is 90.0 kJ/mol.
2. le Claire, A. D., Diffusion of Metals in Metals, pp. 306–379, Prog. Metal Phys. Vol. 1, B. Chalmers, ed., Butterworths, London, (1949).
  A calculated value for the activation energy of movement of single vacancies is quoted as 62.8 kJ/mol. For noble metals and transition metals there is a rough proportionality between the activation energy *Q*_a_ of a diffusing atom and the melting temperature *T*_m_ of the solvent metal. The proportionality is more closely followed for self-diffusion, and for silver *Q*_S_/*T*_m_=155 J/(K·mol), where *Q_S_* = 192 kJ/mol.

### 3.5 Elastic Moduli

1. Boas, W., and Mackenzie, J. K., Anisotropy in Metals, p. 103, Prog. Metal Phys., Vol. 2, B. Chalmers, ed., Pergamon, London (1950).
  Tabulates elastic compliance coefficients *S_ij_* and elastic stiffness coefficients *C_ij_* for crystals having trigonal, tetragonal, hexagonal and cubic symmetry.
2. Chang, Y. A., and Himmel, L., The Temperature Dependence of the Elastic Constants of Cu, Ag, and Au above Room Temperature, J. Appl. Phys. **37**, 3567–72 (1966).
  Adiabatic elastic constants were measured for silver from 300 K to 800 K, tabulated and graphed.
3. Hiki, Y., and Granato, A. V., Anharmonicity in Noble Metals, Higher Order Elastic Constants, Phys. Rev. **144**(2), 411–419 (1966).
  Second-order and third-order elastic constants of Cu, Ag, and Au were measured at room temperature. Complete sets of six third-order elastic constants were obtained. Data are tabulated and plotted.
4. Neighbours, J. R., and Alers, G. A., Elastic Constants of Silver and Gold, Phys. Rev. **111**(3), 707–712 (1958).
†37 †38 †39 †40 †T9The three elastic coefficients *C*=*C*_44_, *C′*=(*C*_11_–*C*_12_)/2, and *C*_L_=(*C*_11_+*C*_12_+2*C*_44_)/2, for Ag and Au were measured over the range 4.2 K < *T* < 300 K. Data are plotted and tabulated. Debye characteristic temperatures obtained from the elastic constants are in good agreement with results from calorimetry.
5. Prasad, B., and Srivastava, R. S., Lattice dynamics of noble metals, J. Phys. F: Metal Phys. **2**, 247 (1972).
  Theoretical matrix elements for the electron-phonon interaction were used to analyze the lattice dynamics of Ag, Au, and Cu. Vibration spectra (dispersion curves), elastic constants and Debye temperatures were calculated, and found to agree with experimental values

#### 3.5.1 Pressure Dependence

1. Daniels, W. B., and Smith, C. S., Pressure Derivatives of the Elastic Constants of Copper, Silver and Gold to 10000 Bars; Phys. Rev. **111**(3), 713–721 (1958).
  The pressure derivatives of the elastic constants of Ag, Au, and Cu were obtained from measurements over the range 0 < *p* < 1 GPa (10 kbar). The derivatives for silver were evaluated using *C* = *C*_44_ = 46.13 GPa, *C*′ = (C_11_−*C*_12_)/2 = 15.28 GPa, the adiabatic bulk modulus *B_S_*=(*C*_11_+2*C*_12_)*_s_*/3 = 103.6 GPa, and the isothermal bulk modulus *B_T_* = 101.5 GPa. For silver the pressure derivatives of the elastic constants are (pressure units of 100 GPa = 10^12^ dyn/cm^2^): d*B_S_*/d*p* = 6.18, d*C*/d*p* =2.31, and d*C*′/d*p* = 0.639. The elastic constants at 0 K are (units of 10^−19^ J/atom): *B* = 18.3, *C* = 8.52, and C′ = 2.84.
2. Guinan, M. W., and Steinberg, D. J., Pressure and Temperature Derivatives of the Isotropic Polycrystalline Shear Modulus for 65 Elements, J. Phys. Chem. Solids **35**, 1501–1512 (1974).
  Experimental values are tabulated for the temperature derivative of the isotropic polycrystalline shear modulus for 47 elements, and pressure derivative for 39 elements.

#### 3.5.2 Young’s Modulus

(See also Sec. **3.8 Mechanical Properties**)

1. Kuhlmann-Wilsdorf, Doris and Wilsdorf, Heinz, The Surface Structures of Deformed Aluminum, Copper, Silver, and Alpha-Brass, and Their Theoretical Interpretation, Acta Metallurgica **1**, 394 (1953).
  Gives average stress-strain curves of super-purity Al, Cu and Ag, and for commercial alpha brass.

Druyvesteyn, M. J., Experiments on the Effect of Low Temperatures on Some Plastic Properties of Metals, App. Sci. Research **1**, 66 (1947?); see Ref. 122, Sec. **3.8 Mechanical Properties**.

McKeown, J. and Hudson, O. F., Stress-strain Characteristics of Cu, Ag and Au; J. Inst. Metals **60**, 109–132 (1937), see Ref. 130, Sec. 3.8 **Mechanical Properties**.

Takamura, S., and Kobiyama, M., Dislocation Pinning in Al and Ag Alloys after Low-Temperature Deformation, Phys. Stat. Sol. (a) **95**, 165–172 (1986), see Ref. 30, Sec. **3.3.1 Dislocations**.

### 3.6 Electrical Resistivity

(See also Sec. **3.15 Transport Properties**)

1. Barber, A. J. and Caplin, A. D., The low temperature electrical resistivity of high purity Ag and Ag based alloys, J. Phys. F: Metal Phys. **5**, 679–696 (1975).
  Electrical resistivities of commercially available high-purity silver (and of alloys of Ag with Au, Pd and Pt) were measured from 2 K to 20 K. Matthiessen’s rule did not hold at all temperatures. Possible dependence of resistivity on temperature as *T*^5^ was investigated; most specimens showed very nearly a *T*^4^ dependence on temperature below 10 K.
2. Barnard, B. R., and Caplin, A. D., ‘Simple’ be-havior of the low temperature electrical resistivity of silver?, Communications Physics **2**, 223–227 (1977). (Same title as that of Ref. 43 by same authors.)
  Electrical resistivity for the temperature range 1.2 K < T < 9 K is plotted for large single crystals of silver. The resistivity varies with temperature as *T*^4^ between 1.2 K and 4 K. From 4 K to 9 K the resistivity varies more slowly than *T*^4^.
3. Barnard, B. R., and Caplin, A. D., ‘Simple’ behavior of the low temperature electrical resistivity of silver?, J. Physique Colloque C6, Suppl. to No. 8, Tome **39**, p. C6-1050–1051 (1978), (Same title as that of Ref. 142.)
  Electrical resistivity for the temperature range 1.2 K < *T* < 9 K is plotted for large single-crystal ingots of silver.
4. Bass, Jack, Electrical Resistivity of Metals and Alloys at Cryogenic Temperatures: A Review, Advances in Cryogenic Engineering (Materials), Vol. 30, Plenum, NY (1984) p. 441.
  Residual resistivity for Ag-Au alloys is plotted as a function of Au concentration, and for pure polycrystalline silver as a function of temperature for 40 K < *T* < 1200 K.
5. Bergmann, A., Kaveh, M., and Wiser, N., Explanation of the anomalous *T*^4^ behaviour of the low-temperature electrical resistivity of silver; J. Phys. F: Metal Phys. **10**, L71–L76 (1982).
  The observed *T*^4^ behavior in the low-temperature (2 K to 6 K) resistivity of silver is shown to result from the scattering of electrons by both phonons and other electrons. The magnitude of the e-e scattering has been successfully calculated.
6. Bergmann, A., Kaveh, M., and Wiser, N., Electrical resistivity of the noble metals at low temperatures: I. Dilute alloys, J. Phys. F: Metal Phys. **12**, 2985–3008 (1982).
  The unusual behavior of the resistivity of the noble metals is discussed in theoretical terms and compared with measurements of the resistivities of Ag, Au, and Cu for 2 K < *T* < 6 K.
7. Bergmann, A., Kaveh, M., and Wiser, N., Electrical resistivity of the noble metals at low temperatures: II. Effect of electron-dislocation scattering, J. Phys. F: Metal Phys. **12**, 3009–3030 (1982).
  The work of the preceding paper (I. Dilute alloys) is extended to the analysis of the resistivities of strained specimens of Ag and Cu.
8. Borchi, E., De Gennaro, S., and Tasselli, P. L., Low-temperature electrical resistivity of noble metals, Phys. Rev. **B12**(12), 5478–5487 (1975).
  The low-temperature electrical resistivity of Ag and Cu were measured over the temperature range 3 K < *T* < 16 K. These data together with other data from the literature are compared with two theories. The resistivity at low temperatures does not follow a well-defined power law; assuming *ρ*≈*T*^n^ then *n* varies continuously from 6 to 4 as *T* varies from 1 K to 15 K. Electron-phonon (normal; Umklapp longitudinal and Umklapp transverse) contributions to the resistivity are tabulated for temperatures from 1 K to 15 K. Resistivities of Ag (*RRR* =10 k) and Cu are plotted for this temperature range.
9. CRC Handbook of Electrical Resistivities of Binary Metallic Alloys, Klaus Schröder, ed., CRC Press, Boca Raton, FL (1983).
  A condensed review of the theory of electronic conduction is followed by a survey of the dependence on temperature and composition of the electrical resistivity of binary alloys of 44 metals.
10. Ehrlich, A. C., and Schriempf, J. T., The Temperature Dependent Thermal and Electrical Resistivity of High Purity Silver From 2 K to 20 K, Solid State Com-mun. **14**, 469–473 (1974).
  The temperature dependences of electrical and thermal resistivities at low temperatures have been measured for a pure specimen (*RRR*=10 k). Below approximately 10 K the electrical and thermal resistivities are respectively proportional to *T*^5^ and *T*^2^: *ρ* (*T*) = *ρ*(*T* = 0) + (7.64×10^16^) · *T*^5^ Ω·cm/K^5^; and *W*_th_ = *W*_0_ + (2.96×10^−5^)*T^2^ cm/(*W*·K).
  Ewbank, M., Imes, J. L., Pratt, W. P., Jr., Schroeder, P. A., and Tracy, J., Measurements of Transport Properties of Silver between 20 mK and 4.2 K, Phys. Lett. **59A**(4), 316–318 (1976), see Ref. 207, Sec. **3.15 Transport Properties**.
11. Guillon, F., Measurements of the low-temperature electrical resistivity of sintered silver powders by acoustic propagation in high magnetic fields, Can. J. Phys. **66**, 963–8 (1988).
  For an orientation of 90° between a specimen disk of sintered silver powder and an applied vertical (6.2 T) magnetic field, a resistivity at 4.2 K of 1.83 μΩ·cm was obtained by an acoustic-propagation method; for 27°, 2.4 μΩ·cm; and for 0°, 1.88 μΩ·cm. These results are compared to a value of 4.0 μΩ·cm obtained by R. J. Robertson, F. Guillon, and J. P. Harrison, Can. J. Phys. **61**, 164 (1983).
12. Guenault, A. M., How Phoney is Phonon Drag?; J. Phys. F: Metal Phys. **1**, L1–L3 (1971).
  A few values for ratios of electronic thermal resistivity are tabulated for silver specimens having *RRR* (residual resistivity ratio) values of 1, 120, and 2000.
13. Gupta, O. P., Temperature Dependence of the Electrical Resistivity of Noble Metals, Acta Phys. Polon. **A49**(3), 331–339 (1976).
  Using the free-electron approximation and the anisotropic continuum dispersive model of Sharma and Joshi, the temperature dependence of the electrical resistivities of Ag, Au, and Cu were calculated. Theoretical values for resistivities are in reasonable agreement with experimental data. Values of electrical resistivities from the literature for all three metals are plotted for 15 K < *T* < 300 K. Elastic constants from the work of Hiki and Granato (Ref. 35) are tabulated.
14. Hall, L. A., Survey of Electrical Resistivity Measurements on 16 Pure Metals in the Temperature Range 0 K to 273 K, NBS Tech. Note 365, Natl. Bur. Stand. (U.S.) (1968).
  Resistivities as functions of temperature (0 K to 273 K) and of pressure 0 to “100 000 kg/cm^2^” (9.8 GPa) [P. W. Bridgman, Proc. Am. Acad. Arts Sci. **81**, 165–251 (1952)] are obtained from the literature, tabulated, and plotted for Ag, Au, Cu, Al, Be, Co, Fe, In, Mg, Mo, Nb, Ni, Pb, Pt, Sn, and Ta.
  Jha, D., and Jericho, M. H., Low-Temperature Transport Properties of Dilute Silver-Manganese Alloys, Phys. Rev. **B3**(1), 147–156 (1971), see Ref. 208, Sec. **3.15 Transport Properties.**
15. Jongenburger, P., The Extra-Resistivity Due to Vacancies in Copper, Silver and Gold; Appl. Sci. Res. **B3**, 237–248 (1953?).
  The contribution to electrical resistivity due to vacancies (extra-resistivity: ER) is calculated for Ag, Au, and Cu. The contributions to ER due to vacancies accompanying the presence of elements next to these three noble metals in the periodic table are tabulated.
  Kannuluik, W. G., The Thermal and Electrical Conductivities of Several Metals Between −183 and 100 C, Proc. Roy. Soc. (London) **A141**, 159–168 (1931); see Ref. 162, Sec. **3.11 Thermal Conductivity.**
16. Khoshenevisan, M., Pratt, W. P., Jr., Schroeder, P. A., and Steenwyk, S. D., Low-temperature resistivity and thermoelectric ratio of copper and gold, Phys. Rev. **B19**(8), 3873–3878 (1979).
  Comparing this work with that of Barnard and Caplin (Refs. 42, 43), and of Lawrence (Ref. 64), the authors verify that the resistivity *ρ* of pure silver is given by: *ρ* = *ρ*_0_ + *B*·*T^N^*, where *N*=4, and *B*=1.4×10^17^ Ω·cm/K^4^. The crossover temperature, where scattering by electrons and phonons should make equal contributions to the resistivity, is 2.4 K. They conclude that there is strong evidence for a *T*^4^ dependence in the low-temperature electrical resistivity of all the noble metals. (See Ref. 57.)
17. MacDonald, A. H., and Laubitz, M. J., Comment on ‘Low-temperature resistivity and thermoelectric ratio of copper and gold’ (See preceding paper by Khoshenevisan et al., Phys. Rev. **B21/6**, 2638–40 (1980).
  Previous high-temperature measurements of the Lorenz function, combined with recent theory, confirm quantitatively the electron-electron scattering term seen by Khoshenevisan et al. (Ref. 56).
18. Khoshenevisan, M., Pratt, W. P., Jr., Schroeder, P. A., Steenwyk, S., and Uher, C., Low-temperature resistivity of silver, J. Phys. F: Metal Phys. **9**(1), L1-L5 (1979).
  For pure polycrystalline silver the dependence of electrical resistivity on temperature follows *ρ*−*ρ*_0_ ∝ *T*^4^ for *T* > 2 K, but at the lowest temperatures the resistivity obeys *ρ*−*ρ*_0_ ∝ *T*^2·19^. The electrical resistivity is plotted for temperatures between 0.04 K and 7 K.
19. Knook, B., and Van den Berg, G. J., The Electrical Resistance of Pure Au and Ag at Low Temperatures, Physica **26**, 505–512 (1960).
  Electrical resistances of wires and strips of Ag and Au at 1.2 K < *T* < 4.2 K were measured, tabulated and plotted as reduced resistance values.
20. Kos, J. F., Relation between the Low-Temperature Thermal and Electrical Resistivities of Ag, Phys. Rev. Lett. **31**(21), 1314–1317 (1973).
  Accurate measurements of the electrical resistivity of silver from 3.5 K to 30 K are examined in the light of theoretical models of Klemens and Kamon, Mathur and Klemens based on phonon-assisted defect scattering. The low-temperature thermal resistivity is discussed in terms of the electrical-resistivity measurements and the Lorenz number. Two plots of thermal resistivity *versus* temperature are given for four sets of data.
21. Kos, J. F., Anomalies in the low-temperature thermal and electrical resistivities of silver, J. Phys.: Con-dens. Matter **2**, 4859–4868 (1990).
  Electrical resistivity and thermal conductivity were measured over the range from 2 K to 23 K and discussed in terms of theory. Results are tabulated and graphed.
22. Kovacs, I., and Nagy, E., Plastic Properties of Polycrystalline f.c.c. Metals, Phys. Stat. Sol. **8**, 795 (1965).
  With simultaneous torsion and extension applied to polycrystalline wires at 78 K, changes in specific resistivity as a function of strain are found to lie on a universal curve when average, rather than torsional, strain is taken as the independent variable. Results for different annealing temperatures are plotted and tabulated.
  Kovacs, I., The Plastic Behaviour of Polycrystalline fcc Metals at Large Strains, Hardening of Metals, P. Feltham, ed., Freund, Tel-Aviv, Israel (1980) pp. 113–164, see Ref. 127, Sec. **3.8 Mechanical Properties**.
23. Krsnik, R., and Babić, E., Influence of the anisotropy of electron-phonon scattering on the low temperature resistivity of normal metals, J. Phys. **39**(8), Colloque C6-1052–1053 (1978).
  The phonon resistivities of Ag, Al, Au, Cu, Sn, and Zn are analyzed in terms of a *T*^5^ variation with temperature and anisotropic electron-phonon scattering.
  Kus, Fred W., Some transport properties of dilute copper and silver alloys, J. Phys. F: Metal Phys. **8**, 1483–89 (1978), see Ref. 209, Sec. **3.15 Transport Properties.**
24. Lawrence, W. E., Electron-electron scattering in the low-temperature resistivity of the noble metals, Phys. Rev. **B13**(12) 5316–19 (1976).
  Electron-electron and electron-phonon contributions to the temperature-dependent resistivity of impure silver are graphed and tabulated.
25. Linde, J. O., Elektrische Eigenschaften verdünnter Mischkristallegierungen: II. Widerstand von Silber-legierungen. (Electrical Properties of Dilute Alloys: II. Electrical Resistance of Silver Alloys), Ann. Phys. (Leipzig), 5. Folge, Band **14**, 353–366 (1932).
†T3The relative change in resistivity per “atomic percent” (mole fraction) of alloying element is tabulated for 16 dilute alloys of metallic elements in silver.
26. Manintveld, J. A., Recovery of the Resistivity of Metals after Cold-Working, Nature **169**, 623 (1952).
†5The changes in relative resistivity versus temperature for Ag, Au, and Cu wires plastically deformed at liquid-air temperatures are plotted, and all are found to show two recovery regions. The two steps in recovery are interpreted in terms of activation energies (*Q*_1_ = 0.18 eV, and *Q*_2_ = 0.65 eV) for a jump of a vacant lattice site.
  Matsumura, T., and Laubitz, M. J., Thermal conductivity and electrical resistivity of pure silver between 80 and 350 K, Can. J. Phys. **48**(12), 1499–1503 (1970), see Ref. 165, Sec. **3.11 Thermal Conductivity.**
27. Matula, R. A., Electrical Resistivity of Copper, Gold, Palladium, and Silver, J. Phys. Chem. Ref. Data **8**(4), 1147–1298 (1979).
†1Recommended values for the electrical resistivities of bulk pure Ag, Au, Cu, and Pd are given as a function of temperature from 1 K to the melting point of the metal. Published values of resistivities from about 1881 to 1976 are used to establish the recommended values. A brief theoretical background and a discussion of the analysis of the data are given, including use of a correction for the effect of thermal expansion with temperature.
28. MacDonald, D. K. C., Electrical Conductivity of Metals and Alloys at Low Temperatures, Encyclopedia of Physics, Vol. XIV: Low Temperature Physics I, Springer, Berlin (1956) pp. 137–197.
  The experimental techniques for measuring electrical conductivity are reviewed and existing data are compared with theory.
29. Meaden, G. T., Electrical Resistance of Metals, Plenum, NY (1965).
  The theory and experimental results of the electrical resistance of metals and alloys, and deformed and irradiated metals, as it varies with temperature, chemical impurities, physical state (vacancies, dislocations, or interstitials), applied pressure, magnetic field and dimensions of the specimen (size effect) are reviewed.
30. Nagata, S., Ogino, M., and Taniguchi, S., Electrical Resistivity of Thin Metal Films Vapor-Quenched at 77 K, Phys. Stat. Sol. (a) **102**, 711 (1987).
  Thin metal films, approximately 30 nm to 110 nm thick, of Ag, Au, Cu, Ni, Pd, and Pt were evaporated onto glass substrates at 77 K. The temperature dependences of the electrical resistivities of the films were measured and plotted for different processes of annealing at temperatures from 110 K to 330 K. Temperature coefficients of the resistivity are tabulated for those temperature ranges for which the resistivity changes are reversible.
31. Pawlek, F., and Rogalla, D.,The Electrical Resistivity of Silver, Copper, Aluminum, and Zinc as a Func tion of Purity in the Range 4–298 K; Cryogenics **6**(1), 14–20 (1966).
  Figures and tables of ideal and measured resistivi-ties versus temperature for specimens of Ag, Al, Cu, and Zn of different purities are given for the range 4 K to 298 K. A table of resistivity (magne-toresistance) of purest commercial samples in a transverse magnetic induction of 1.8 *T* is given for measurements at 4.2 K and 20 K.
32. Rowlands, J. A., and Woods, S. B., Anisotropic electron scattering in the resistivity of strained aluminum, palladium and silver, J. Phys. F: Metal Phys. **8**(9), 1929–1939 (1978).
  The electrical resistivities of annealed and strained specimens of Ag, Al, and Pd were measured at temperatures below 40 K. The temperature-dependent resistivity component *ρ_T_* is plotted as a function of *ρ*_0_, on a log-log scale, for temperatures of 10 K, 13 K, 20 K, 30 K and 49 K.
  Rumbo, E. R., Transport Properties of Very Pure Copper and Silver below 8.5 K, J. Phys. F: Metal Phys. **6**(1), 85–98 (1976), see Ref. 211, Sec. **3.15 Transport Properties**.
33. Sathish, S., and Awasthi, O. N., Electron-electron Scattering and Low-Temperature Electrical Resistivity in Copper and Silver, Phys. Lett. **100A**, 215–17 (1984).
  Resistivity data from the literature for Ag and Cu are analyzed in terms of electron-electron Umklapp scattering processes.
34. Schroeder, P. A., Blumenstock, B., Heinen, V., Pratt, W. P., Jr. and Steenwyk, S., Resistivity Measurements on High Purity Cu, Ag and Al Between 40 mK and 1.5 K., Physica **107B**, 137–8 (1981).
  The logarithmic derivative of resistivity is plotted as a function of temperature from 0.2 K to 1.5 K.
35. Steenwyk, S. D., Rowlands, J. A., and Schroeder, P. A., Effect of impurities and dislocations on the temperature-dependent resistivity of the noble metals, J. Phys. F: Metal Phys. **11**, 1623–1633 (1981).
  Previous data for the electrical resistivities of pure Ag, Au, and Cu have been reanalyzed to pin down the exponent(s) in the power-law dependence of the resistivities. Powers of 2, 4, or both, in the resistivity, were considered.
36. White, G. K., and Woods, S. B., Electrical and Thermal Resistivity of the Transition Elements at Low Temperatures, Phil. Trans. Roy. Soc. London **A251**, No. 995, 273–302 (1959).
  Thermal and electrical resistivities were measured for 20 transition elements over the range from 2 K to 300 K. The ideal resistivities *W*_i_ and *ρ*_i_ are tabulated and plotted. The three silver specimens were annealed, and of very high (“99.999 %”) purity; the *RRR* values were 261, ~1000, and ~2000.
37. Yamashita, J., and Asano, S., Electrical resistivity of Cu- and Ag-based dilute alloys at low temperatures, J. Phys. F: Metal Phys. **16**, 2063–2077 (1986).
  Theoretical results for the influence of the electron-phonon interaction on electrical resistivity are compared with data from the literature for pure Ag and Au and their dilute alloys at low temperatures.

#### 3.6.1 Change With Deformation

1. Aarts, W. H., and Jarvis, R. K., The Change in Resistivity, on Plastic Deformation, of Silver-Copper and Silver-Gold Alloys, Acta Metall. **2**, 87 (1954).
  Relative change of resistivity (*RR*) with strain for wires of pure Ag, pure Cu, Ag-Cu alloys, Ag-Au alloys, and pure Au are graphed and tabulated. For pure Ag, *RR* increases as wires are strained at the liquid-air point to approximately 18 % extension; upon warming to room temperature, *RR* of the wires decreases by one third. Further strain at liquid-air temperature increases *RR* with a slope (*RR* vs strain) steeper than that before the wires were warmed.
2. Barnard, B. R., Caplin, A. D. Dalimin, M. N. B., The effect of cold work on the low-temperature electrical resistivity of Ag, Phil. Mag. **B44**(6), 711–729 (1981).
  The incremental change in electrical resistivity of silver from 1 K to 9 K, at various stages of cold work, is plotted. The value of the Lorenz number obtained is similar to the Sommerfeld value (2.45×10^−8^ (V/K)^2^).
3. Barnard, B. R., Caplin, A. D., and Dalimin, M. N. B., The electrical resistivity of Ag and Ag-based alloys below 9 K, J. Phys. F: Metal Phys. **12**, 719–744 (1982).
  Differential electrical resistivities of nominally pure silver and silver alloys from 1.2 K to 9 K, at various stages of cold work, are plotted. The Lorenz number is similar to the Sommerfeld value (2.45×10^−8^ (V/K)^2^). Contributions to the low-temperature resistivity of silver, from impurities, dislocations, and surfaces, and from electron-phonon, electron-electron and Koshino-Taylor scattering, are estimated and tabulated.
  Barnard, R. D., The true low-field Hall coefficient of high-purity silver and silver containing low-density defects at low temperatures, J. Phys. F: Metal Phys. **7**(4), 673–691 (1977), see Ref. 89] Sec. **3.6.3 Hall Coefficient.**
4. Berghout, C. W., Increase of the resistivity of some face centered metals by cold-working, Physica **18**(11), 978–979 (1952).
†3The areas of annealed wires of Ag, Au, Al, and Cu were reduced by cold working (drawing through dies at room temperature). The fractional increase in resistivities of Ag and Cu wires at room and liquid-air temperatures are plotted as a function of areal reduction to see whether change in resistivity due to cold working is independent of temperature (Matthiessen’s rule applies). For Cu the change in resistivity is independent of temperature, but for silver deviations from Matthiessen’s rule were found.
5. Broom, T., Lattice Defects and the Electrical Resistivity of Metals, Adv. Phys. **3/9**, 26–83 (1954).
  The theory and experiments dealing with the effect of lattice defects on electrical resistivity in pure metals and alloys are reviewed. Because deformed metals generally obey Matthiessen’s Rule, the absolute change in resistivity Δ*ρ* is generally independent of temperature. From an empirical point of view, however, it is convenient to use the relative change in resistivity Δ*ρ*/*ρ*_0_ where *ρ*_0_ is the reference value of resistivity. The reference value is usually taken to be that measured at room temperature. For 10 % extension of silver, the relative change in resistivity at room temperature is 0.5 % (for a reference value *ρ*_0_ = 1.59 μΩ·cm at 20 °C). After drawing of silver wire to a 98 % reduction in cross section, the relative increase in resistivity is 5 %. Values of Δ*ρ* after irradiation of silver at −140 °C by 12 MeV deuterons are plotted as a function of deuteron flux.
6. Molenaar, J., and Aarts, W. H., Change of Resistivity by Cold Working at Liquid-Air Temperature, Nature **166**, 690 (1950).
  Relative changes of resistivity with relative extension (strain) for Ag and Cu wires at 90 K are plotted; the silver wire was first drawn to 12 % extension, soft-annealed, and then further stretched to 17 %. Although the tension-deformation curve showed no discontinuity, the relative resistivity was reduced at the point where the deformation was halted for warming the specimen. The magnitude of the discontinuity in relative resistivity depends sensitively on the annealing temperature.

#### 3.6.2 Damage by Irradiation

1. Averback, R. S., Benedek, R., and Merkle, K. L., Ion-irradiation studies of the damage function of copper and silver, Phys. Rev. **B18**(8), 4156–4171 (1978).
  Ions (H, D, He, Li, B, C, N, O, Ne, Cl, Ar, Fe, Cu, Kr, Ag, and Bi) with kinetic energies of the order of tens to hundreds of keV were used to irradiate thin foils of Ag and Cu (thick enough to stop the incident ions). The resulting damage increased the electrical resistivity of the foils. Results are tabulated and graphed.
  Broom, T., Lattice Defects and the Electrical Resistivity of Metals, Adv. Phys. **3**(9), 26–83 (1954), see Ref. 82.
2. Coltman, R. R., Klabunde, C. E., and Redman, J. K., Survey of Thermal-Neutron Damage in Pure Metals, Phys. Rev. **156**(3), 715–734 (1967).
  Damage in 14 high-purity metallic elements was caused by capture (98.2 % thermal in silver) of neutrons at 3.6 K, producing a change in electrical resistivity. Recovery was programmed with 60 heating pulses 5 min in duration. The isochronal recovery from damage by thermal-neutron capture is plotted as a function of temperature. The isochronal recovery for silver shows a largest peak at 30 K, a second-highest peak at 300 K, and two smaller peaks at 170 K and 140 K attributed to the effects of impurities.
3. Lucasson, P. G., and Walker, R. M., Production and Recovery of Electron-Induced Radiation Damage in a Number of Metals, Phys. Rev. **127**(2), 485–500 (1962).
†7The residual electrical resistances of pure specimens of Ag, Al, Au, Cu, Fe, Mo, Ni, Ti, and W, at temperatures of 20 K, were changed by high-energy (0.5 MeV to 1.4 MeV) electron bombardment. The resistivity change per unit electron flux is plotted as a function of average electron energy for Ag, Al, Cu, Fe, and Ni.
4. Lucasson, P. G., and Walker, R. M., Variation of Radiation Damage Parameters in Metals, Phys. Rev. **127**(4), 1130–1136 (1962).
  Normalized recovery of electrical resistivity Δ*ρ*/Δ*ρ*_0_ after electron bombardment is plotted as a function of reduced temerature for seven metals: Ag, Al, Au, Cu, Fe, Ni, and Mo. A table compares results from neutron damage with the results of electron bombardment, for the seven metals studied here.

#### 3.6.3 Hall Coefficient

1. Alderson, J. E. A., Farrell, T., and Hurd, C. M., Hall Coefficients of Cu, Ag, and Au in the Range 4.2–300 °K, Phys. Rev. **174**(3), 729–736 (1968).
†13The Hall coefficients for polycrystalline Ag, Au and Cu, two single crystals of Cu, and dilute alloys of Ag (with Au, Cd and Zn) were determined for 4.2 K < *T* < 300 K and in a magnetic field of 1.5 T. The temperature dependences of the Hall coefficients for each metal are plotted over the full temperature range of measurement.
2. Barnard, R. D., The true low-field Hall coefficient of high-purity silver and silver containing low-density defects at low temperatures, J. Phys. F: Metal Phys. **7**(4), 673–691 (1977).
†14 †15 †16 †17Relative Hall coefficient *R*_H_(*T*)/*R*_H_(77 K) is plotted as a function of temperature and of magnetic flux density. The purity of specimens was nominally “99.9999 %,” and residual resistivity ratios were 3250, 3550, and 510, for samples 1, 2 and 3, depending on oxidation of Fe impurities and annealing. The presence of defects decreased the *RRR* values to 270, 625 and 720 for samples 1, 2A, and 2B. Plots of relative Hall coefficients are also given for Ag containing small impurity contents of Au. The variation of the relative characteristic peak Hall coefficient with magnetic induction *B* is plotted for three different values of residual resistivity ratio (*RRR*).
3. Springford, M., The Anisotropy of Conduction Electron Scattering in Noble Metals, Adv. Phys. **20**, 506–9 (1971).
  At 300 K the measured Hall coefficient *R*_H_ is −8.81×10^−11^ m^3^/(A·s), while the calculated free-electron value *R*_F_ is −10.65×10^−11^ m^3^/(A·s). The dimensionless parameter n*_(observed)_ = 1/*RNe*=1.21, where *R* is the low-field Hall coefficient and *N* is the number of atoms per unit volume; n*(calc) = 1.58.

#### 3.6.4 Lorenz Number

Barnard, B. R., Caplin A. D., and Dalimin, M. N. B., see Refs. 77 and 78; Sec. **3.6. Electrical Resistivity.**

1. Fenton, E. W., Rogers, J. S., and Woods, S. B., Lorenz Numbers of Pure Aluminum, Silver, and Gold at Low Temperatures, Can. J. Phys. **41**, 2026–2033 (1963).
  The thermal and electrical conductivities of pure Al, Ag and Au were measured from 2 K to 31 K; the variation of Lorenz number with temperature was calculated from the data.
  Kannuluik, W. G., The Thermal and Electrical Conductivities of Several Metals Between −183 and 100 C; Proc. Roy. Soc. (London) **A141**, 159–168 (1931), see Ref. 162, Sec. **3.11 Thermal Conductivity.**
  Laubitz, M. J., Transport properties of pure metals at high temperatures. II. Silver and Gold, Can. J. Phys. **47**, 2633–2644 (1969), see Ref. 210, Sec. **3.15 Transport Properties.**
2. Laubitz, M. J., Electron-Electron Scattering in the High-Temperature Thermal Resistivity of the Noble Metals, Phys. Rev. **B2**(6), 2252–4 (1970).
  The electronic Lorenz function is plotted as a function of reduced temperature *T*/Θ_R_ for 1.3<*T*/Θ_R_<5, where Θ_R_ is the Debye temperature obtained from electrical-resistivity data. Reported deviations of the Lorenz function for the noble metals at high temperatures can be explained in terms of electron-electron scattering.

Lees, Charles H., The Effects of Temperature and Pressure on the Thermal Conductivities of Solids—Part II. The Effects of Low Temperatures on the Thermal and Electrical Conductivities of Certain Approximately Pure Metals and Alloys, Phil. Trans. Roy. Soc. (London) **A208**, 381–443 (1908), see Ref. 164, Sec. 3.11 **Thermal Conductivity.**

Rumbo, E. R., Transport Properties of Very Pure Copper and Silver below 8.5 K, J. Phys. F: Metal Phys. **6**(1), 85–98 (1976), see Ref. 211, Sec. **3.15 Transport Properties**.

#### 3.6.5 Magnetoresistance

1. Aschermann, G., and Justi, E., Elektrische Leit-fa¨higkeit, magnetische Widerstandsvermehrung, Hall-effekt und Supraleitung von Rhenium [Electrical Conductivity, Increase in Magnetoresistance, Hall Effect and Superconductivity in Rhenium], Phys. Zeitschrift **43**, 207–212 (1960) (German).
  Resistance ratios for rhenium are tabulated and plotted. A Kohler plot (Δ*ρ_H_, T*/*ρ*_0_,*T versus H*/*r_T_*) compares the magnetoresistance of Re as a function of reduced field to the magnetoresistances of 25 metals including silver.
2. Atkinson, A., The low-field magnetoresistance of single crystals of dilute copper- and silver-based alloys, J. Phys. F: Metal Phys. **1**, 863–876 (1971).
  The low-field magnetoresistances of single crystals of dilute Ag- and Cu-based alloys were measured at 1.5 K and 4.2 K. At low fields the fractional change in resistivity, Δ*ρ*/*ρ*, is given by *K*·(*B*/*ρ*)^2^, where *K* is independent of magnetic induction *B* and resistivity *ρ*_0_. For longitudinal applied fields, *K*_l_ = *b* + *c* + *ld*, where *l* depends only upon crystal orientation, *b* = 1.00, *c* = −0.97, and *d* = 1.67 (all three coefficients in units of (Ω·cm/T)^2^×10^−16^). For transverse fields, *K*_t_ = *b* + *t*(θ)*d*, with the coefficients *b* and *d* taking the same values as those for the longitudinal fields; *t*(θ) is a function of both crystal orientation and direction of *B*. The reduced Hall coefficient *ρ*, defined as the ratio of the actual Hall coefficient to that expected from the free-electron model, was calculated as 0.81, compared with an experimental value of 0.83.
3. Cavalloni, C., and Joss, W., Weak Localization and Oscillatory Magnetoresistance in Cylindrical Ag Films, Sol. State Commun. **59**(7), 437–440 (1986).
  Thin cylindrical films of 99.9999 % pure silver were formed by evaporating silver onto rotating glass fibers at room temperature. The magnetore-sistance in the weakly localized regime was investigated in longitudinal magnetic fields (up to 0.3 *T*) and at four different temperatures from 1.58 K to 4.2 K. At fields of less than 8 mT, oscillations in magnetoresistance were observed with a period of *h*/2*e* (the magnetic flux quantum), and confirms the predictions of a theory of Altshuler, Aronov and Spivak [Pis’ma Zh. Eksp. Teor. Fiz. **33**, 101 (1981)]. This effect is due to quantum interference between pairs of multiply scattered electron waves. Plots (0 < *H* < 10 mT and 0 < *H* < 0.3 T) of magnetoresistance (*R*(*H*)-*R*(0))/*R*^2^(0) versus magnetic field *H*, are given for temperatures of 1.58 K, 2.13 K, 2.95 K and 4.2 K. (Note that the authors use a non-standard definition of magnetoresistance, and give units of Tesla for magnetic field *H*.)
4. Dallaire, L., and Destry, J., Application of the Pippard-Klemens-Jackson theory of longitudinal mag-netoresistance to a model of scattering on the Fermi surface of copper, silver and gold, Phys. Rev. **B28**(6), 2947–2956 (1983).
  The ratio of “saturated” electrical resistivity to zero-field value, *ρ*_∞_/*ρ*_0_, as a function of temperature (0 K < *T* > 40 K) was calculated in the approximation of the small-angle scattering model. Calculated values for copper are shown in a plot to agree well with measurements by others. Using the present model, the resistivity ratios versus temperature were calculated for silver and gold, in the range 0 K < *T* > 40 K, for (100), (110), and (111) orientations, and plotted. There is a broad peak in the longitudinal magnetoresistances of silver at about 4 K for all three orientations.
5. Giordano, N., and Pennington, M. A., Two-dimensional weak localization in combined perpendicular and parallel magnetic fields, Phys. Rev. **B47**(15), 9693–9705 (1993).
  The magnetoresistances of thin films of Ag, Au, and Fe-doped Au were measured at 1.35 K and 4.2 K in magnetic fields both parallel and perpendicular to the plane of the film. Magnetoresistance is plotted as a function of applied fields (parallel, perpendicular, and combined) from 0 T to 2 T.
  Guillon, F., Measurements of the low-temperature electrical resistivity of sintered silver powders by acoustic propagation in high magnetic fields, Can. J. Phys. **66**, 963–8 (1988), see Ref. 51, Sec. **3.6 Electrical Resistivity.**
6. Iwasa, Y., McNiff, E. J., Bellis, R. H., and Sato, K., Magnetoresistivity of silver over temperature range 4.2–159 K, Cryogenics **33**(8), 836 (1993).
†2Resistivity *ρ* is plotted as a function of temperature and of magnetic induction. A Kohler plot shows magnetoresistance, Δ*ρ*(*T*, *B*)/*ρ*_0_(*T*) as a function of *B*·*ρ* (273 K)/*ρ* (*T*), for silver of “99.99 % purity.” Data are given for the temperature range 4.2 K < *T* > 159 K, and for magnetic induction 0 T < *B* < 14.8 T.
7. Jenkins, R. G., Jones, H., Belenli, I., Yang, M., Goringe, M. J. and Grovenor, C. R. M., Orientation and thermal cycling effects on the critical currents of high *T*_C_ superconducting composites, Cryogenics **33**(1), 81–85 (1993).
  Transport critical currents were measured as functions of magnetic fields applied parallel and perpendicular to the surfaces of superconducting composite tapes of high critical temperature, *T*_C_. Normalized resistances at 4.2 K are plotted as a function of applied field up to 9 T; this magnetore-sistance is not presented as being definitive, but as a qualitatively useful result. The anisotropy factor, 1–*I*_C_(B*∥c*)/*I*_C_(*B*⊥*c*), and critical current densities are plotted as functions of parallel or perpendicular fields up to 2 T.
8. Lüthi, B., Widerstandsa¨nderung von Metallen in hohen Magnetfeldern (Change in Resistance of Metals in High Magnetic Fields), Helv. Phys. Acta **33**, 161–182 (1960) (German).
†2The transverse and longitudinal magnetoresis-tances of polycrystalline metals (Ag, Al, Au, Cu, Fe, In, Li, Ni, Pb, Pt, Sn, and Zn) were measured in pulsed magnetic fields up to 22 T, at 4.2 K and 80 K. Kohler plots (Δ*ρ*(*T*, *B*)/*ρ*_0_(*T*) as a function of *B*·*ρ* [273 K)/*ρ* (*T*)] of relative change in resistance in both transverse and longitudinal fields are given.
9. McConville, Paul, and Birge, Norman O., Weak localization, universal conductance fluctuations, and 1/f noise in Ag, Phys. Rev. **B47**(24), 16667–16670 (1993).
  The magnetoresistance and the magnetic-field dependence of the 1/*f* noise in thin films of silver (purity unspecified) were measured over the range of temperatures from 1 K to 25 K. Data were compared to theories of weak localization and of universal conductance fluctuations. Magnetoresis-tance is plotted for temperatures of 1.0 K, 4.4 K, 10 K and 25 K for magnetic fields of 10^−4^ to 0.5 T.
10. Neubert, W., Magnetische Widerstandsa¨nderung von Kupfer- und Silber-Einkristallen (Change in Magnetoresistance of Single Crystals of Copper and Silver), Zeitsch. Naturforsch. **A24**(6), 922–929 (1969) (German).
  Magnetoresistance of single crystals of Ag and Cu was measured in weak magnetic fields at 4.2 K. The relative change in resistance Δ*R*/*R* is plotted as a function of the square of magnetic induction (*B*^2^).
11. Pawlek, F., and Rogalla, D., The Electrical Resistivity of Silver, Copper, Aluminum and Zinc as a Function of Purity in the Range 4–298 K, Cryogenics **6**(1), 14–20 (1966).
  Figures and tables of ideal and measured resistivities versus temperature for specimens of Ag, Al, Cu, and Zn of different purities are given for the range 4 K to 298 K. A table of resistivity of purest commercial samples in a transverse magnetic induction of 1.8 T (magnetoresistance) is given for measurements at 4.2 K and 20 K.
12. van Witzenburg, W., and Laubitz, M. J., Magnetoresistances and the Phonon Conductivity of Metals, Can. J. Phys. **46**, 1887–1894 (1968).
  Electrical and thermal magnetoresistances were determined at a maximum field of 4.46 T, over the range 80 K < *T* > 130 K. Results were interpreted in terms of “normal” and “magnetic” Wiedemann-Franz ratios for Ag, Au, and Cu. These “normal” and “magnetic” ratios were plotted. Parameters for the phonon conductivity are tabulated. Results were applied to data for conductivity of tungsten.

#### 3.6.6 Matthiessen’s Rule

1. Barnard, R. D., Some Remarks on Deviations from Matthiessen’s Rule in Dilute Alloy Systems, Phys. Stat. Sol. (b) **104**, 613–620 (1981).
  The role of dislocations as scattering centers in the silver alloy system is interpreted with regard to the failure of silver to follow the universal curve of *ρ_T_* versus *ρ*_0_.
2. Bass, Jack, Deviations from Matthiessen’s Rule, Adv. Phys. **21**, 431–604 (1972).
  Deviations from Matthiessen’s rule are comprehensively reviewed and sources of deviations are discussed: in substitutional alloys, after quenching, radiation damage, and plastic deformation, and in thin specimens.
  Berghout, C. W., Increase of the resistivity of some face centered metals by cold-working, Physica **18**(11), 978–979 (1952), see Ref. 81, Sec. **3.6.1 Change with Deformation.**
3. Cimberle, M. R., Bobel, G., and Rizzuto, C., Deviations from Matthiessen’s rule at low temperatures: An experimental comparison between various metallic alloy systems, Adv. Phys. **23**, 639–674 (1974).
  Electrical resistivity at low temperatures is reviewed for both pure specimens and alloys of Ag, Al, Au, Cu, Mg, Pt, Sn and, Zn, in connection with deviations from Matthiessen’s rule. The temperature-dependent part (*ρ* − *ρ*_0_) of resistivity is plotted as a function of *ρ*_0_, the residual resistivity, for variation of *ρ*_0_ by four to five orders of magnitude.
4. Dugdale, J. S., and Basinski, Z. S., Matthiessen’s Rule and Anisotropic Relaxation Times, Phys. Rev. **157**(3), 552–560 (1967).
  Relative departures Δ/*ρ*_0_(Δ= *ρ*_tot_−[*ρ*_ph_+*ρ*]) from Matthiessen’s rule (MR) as a function of temperature were studied for strained Ag and Cu and for some dilute alloys of Ag and of Cu. At temperatures where the residual resistivity dominates, deviations from MR become as large as or greater than the ideal resistivity.
5. Goodarz, M., and Barnard, R. D., Deviations from Matthiessen’s Rule in Silver Containing Low-density Defects, Phys. Stat. Sol. (b) **83**, 555 (1977).
  Deviations from Matthiessen’s rule (DMR) are studied in the light of new measurements. The total DMR Δ has three parts: one (Δ_1_) due to impurity and phonon scattering, a second (Δ_2_) due to inelastic coherent scattering, and a third (Δ_3_) due to addition of charged impurities. Three plots of Δ/*ρ*_0_ versus temperature from 4 K to 300 K are given for strained silver, where the values of residual resistivity *ρ*_0_ were 0.009 μΩ·cm, 0.04 μΩ·cm, and 0.0259 μΩ·cm.
6. Kos, J. F., Determination of the Ideal Resistivity and of the Deviation from Matthiessen’s Rule in Silver, Can. J. Phys. **51**, 1602–1618 (1973).
  The temperature dependence of the electrical resistivity of strained and annealed specimens of “very pure” silver (values of *RRR* from 144 to 1600) was measured over the range 1.4 K < *T* > 295 K. The deviation from Matthiessen’s rule was determined. A function for the ideal resistivity between 12 K and 23 K attributed to normal and umklapp scattering is given: *ρ*_i_ = 1.35×10^−15^ · *T*^4·88^ · [1−0.008(Θ/*T*)^5^) · exp(Θ/*T*)]. Log (*ρ*_i_) is plotted as a function of log (*T*).

#### 3.6.7 Pressure Dependence

1. Bridgman, P. W., The Resistance of 72 Elements, Alloys and Compounds to 100,000 kg/cm^2^, Proc. Am. Acad. Arts Sci. **81**(4), 165–251 (1952).
†4 †T4Data for differential relative changes in resistance with pressure (d(d*R*/*R*_0_)/d*p*) are given for Ag from 0 to “100 000kg/cm^2^” (9.8 GPa).
2. Goree, W. S., and Scott, T. A., Pressure Dependence of Electrical Conductivity of Metals at Low Temperatures; J. Phys. Chem. Solids **27**, 835–848 (1966).
  The electrical resistances of high-purity (“99.5” and “99.999 %”) wires of Ag, Au, In, and Sn were measured at pressures up to 608 MPa (6 kbar), at temperatures between 4.2 K and 297 K. Results are graphed. The contributions from lattice vibrations and impurities to the total resistivity coefficient are obtained and analyzed in terms of Bloch-Grüneisen theory. At 4.2 K the resistance was measured up to a pressure of 810 MPa (8 kbar). Plots are given for resistance isobars as a function of temperature, resistance *versus* temperature, fractional change in resistivity per change in pressure *versus* temperature, fractional change in lattice resistivity per change in pressure *versus* temperature, and dln*ρ*_L_/dln*V versus* (1 + ∂ln*ρ*_L_/∂ln*T*).
  Hall, L. A., Survey of Electrical Resistivity Measurements on 16 Pure Metals in the Temperature Range 0 to 273 K, NBS Tech. Note 365, Natl. Bur. Stand. (U.S.) (1968), see Ref. 54, Sec. **3.6 Electrical Resistivity**.
3. Hatton, J., Effect of Pressure on the Electrical Resistance of Metals at Liquid Helium Temperatures, Phys. Rev. **100**(2), 681–684 (1955).
  The effect of pressures up to “5000 kg/cm^2^” (490 MPa) on electrical resistances of polycrystalline “fine silver” (purity unknown), as well as Au, Cu, Pt, As, Sb, and Bi were measured at LHe temper atures. All the metals investigated showed hysteresis in their resistance-pressure dependences. The resistance of silver rose nearly linearly with applied pressure (461 MPa max.) but due to hysteresis the resistance decreased approximately parabolically to a zero-pressure value *P*_01_ slightly more than 0.5 % higher than the initial zero-pressure value P_00_. A second cycle to maximum pressure and back followed the same pattern of hysteresis.
4. Lawson, A.W., The Effect of Hydrostatic Pressure on the Electrical Resistivity of Metals, Prog. Metal Phys., Vol. 6, B. Chalmers and R. King, eds., Pergamon, London (1956) pp. 1–44.
†T4Reviews data of P. W. Bridgman [Proc. Am. Acad. Arts Sci. **70**, 225 (1935); **79**, 125 (1951), **81**, 165 (1952) (Ref. 111), **82**, 71 (1953)] and tabulates resistance and volume of metals versus applied pressure up to “10^5^ kg/cm^2^” (9.8 GPa), for metals from six groups of the periodic table, and for rare-earth and transition metals.

### 3.7 Magnetic Susceptibility

1. CRC Handbook of Chemistry and Physics, R. C. Weast, and M. J. Astle, eds., Magnetic Susceptibility of the Elements and Inorganic Compounds, CRC Press, Boca Raton (1982) p. E-118.
  The magnetic susceptibility of Ag(s) at 296 K is given as −0.181×10^−6^ emu/g.
2. Henry, W. G., and Rogers, J. L., The Magnetic Susceptibilities of Copper, Silver and Gold and Errors in the Gouy Method, Phil. Mag. **1**, 223–236 (1956).
  The absolute magnetic mass susceptibility of silver is given as −0.1813×10^−6^ emu/g, at room temperature.
3. Pugh, E. W., and Ryan, F. M., Magnetic Susceptibility of Copper-Nickel and Silver-Palladium Alloys at Low Temperatures, Phys. Rev. **111**(4), 1038–1042 (1958).
  Gives the magnetic susceptibility of silver as −0.182×10^−6^ + 0.07×10^−10^·*T* in unspecified units (by comparison to results of Henry and Rogers, one can assume that the units are mass susceptibility, emu/g).

### 3.8 Mechanical Properties

1. Ashby, M. F., Fracture Mechanisms in Simple Tension, Prog. Matls. Sci., Vol. 4, B. Christian, Haasen, and Massalski, eds., Pergamon, Oxford (1981) p. 112.
  A fracture-mechanism map for round bars of commercially pure silver tested in tension (nominal tensile stress/Young’s modulus vs homologous temperature, *T*/*T*_m_; *T*_m_ = 1234 K) is given.
2. Basinski, Z. S., Thermally Activated Glide in Face-Centred Cubic Metals and its Application to the Theory of Strain Hardening, Phil. Mag. **4**, 393–432 (1959).
  For polycrystalline Al, and single crystals of Ag, Cu, Al: a table and plot of influence of strain-rate and temperature on flow stress, plus plots of force versus strain for dislocations, are given.
3. Carreker, R. P., Jr., Tensile Deformation of Silver as a Function of Temperature, Strain Rate, and Grain Size, Trans. AIME, J. Metals **9**, 112–115 (1957).
†45 †46For silver of three grain sizes (0.014 mm, 0.040 mm and 0.250 mm) and of “0.9997 + purity”: yield strength, tensile strength, and percent elongation are plotted as functions of temperature from 0 K to approximately 1173 K; true stress is plotted as a function of true plastic strain for eight different temperatures: 20 K, 78 K, 195, K 205 K, 299 K, 473 K, 673 K and 873 K, after annealing temperatures of 973 K, 1073 K and 1173 K; flow stress at selected strains is plotted as a function of temperature for silver annealed at 973 K; and strain-rate sensitivity is plotted over the range 20 K to 1173 K. Correlations are also given with grain size.
4. Dotsenko, V. I., Kononenko, V. I., Parkhomenko, T. A., and Pustovalov, V. V., Peculiarities of the Rate Dependence of the Low Temperature Yield Points of Metals with an f.c.c. Lattice, Fiz. Metal. Metalloved **39**(5),1103–1106 (1975).
  The dependence of yield point of Ag, Cu, and Ni at 1.5 K, 77 K and 300 K, and the temperature dependence of response to deformation rate from 4 K to 300 K, are plotted for Ag, Cu, and Ni, and activation volume for Ag single crystals, from 4 K to 78 K.
5. Druyvesteyn, M. J., Experiments on the Effect of Low Temperatures on Some Plastic Properties of Metals, App. Sci. Research **1**, 66 (1947?).
  Yield point and modulus of elasticity for silver strips, at 20 °C and −183 °C, are tabulated, as are Vickers hardness (20 °C) and Brinell hardness (20 °C and −183 °C).
6. Edwards, C. A., Phillips, D. L., and Liu, Y. H., The Yield Point in Steel; J. Iron and Steel Inst. **147**, 145–167, comments: 168–172 (1943).
  Contrary to earlier belief that the yield point in mild steel should be attributed to its body-centered cubic (bcc) lattice structure, this work shows that non-ferrous metals or alloys, such as Ag, Ni, Mn-Ni, Be-Cu, or Duralumin, with face-centered cubic (fcc) structure, can show yield points. The yield strain of “standard silver” (of undefined composition) was found to be 1.5 % for a specimen quenched from 750 °C, strained 6 %, then tempered for 1 h at 175 °C. Specimens quenched and tempered, but not strained, showed no yield point. Stress is plotted as a function of elongation to 28 %.
7. Everhart, J. L., Lindlief, W. E., Kanegis, J., Weissler, P. G., and Siegel, F., Mechanical Properties of Metals and Alloys, NBS Circular C447, Natl. Bur. Stand. (U.S.) (1943).
†32For silver, values of mechanical properties are given from a paper by McKeown and Hudson (Ref. 130), and from NBS Circular 101 (Ref. 133) and NBS Circular 412 (Ref. 15). Rockwell hardness (B and F) and tensile strength versus percent reduction (0 % to 68 %) by rolling are plotted for “fine” and Sterling silver. Plots tensile strength versus temperature for “fine” and Sterling silver and silver alloys from 78 °F to 1200 °F.
8. Hauser, Joachim J., Yield Point and Easy Glide in Silver Single Crystals, Trans. Metallurgical Soc. AIME **221**, 305–309 (1961).
  Stress *versus* strain for silver single crystals under conditions of easy glide is plotted, as well as results of a Bauschinger test on silver single crystals. The test temperature, unstated, is assumed to be room temperature.
9. Hutchison, M. M., and Honeycombe, R. W. K., Some Yield Phenomena in Polycrystalline Silver-Base Alloys, Metal Science J. **1**, 129–131 (1967).
  Initial yield *versus* elongation for pure silver and alloys of silver, at −196 °C, are briefly examined. The alloys were made with mole ratios of 6 parts M in 100 parts Ag+M, where M = Al, As, Au, Ga, Ge, Sb, or Sn. Variation of load *versus* elongation is plotted over the temperature range from −200 °C to 100 °C.
10. Kovacs, I., The Plastic Behaviour of Polycrystalline fcc Metals at Large Strains, Hardening of Metals, P. Feltham, ed., pp. 113–164, Freund, Tel-Aviv, Israel (1980).
  Change of electrical resistivity with tensile strain, for different annealing temperatures; torsional stress-strain curves for three different temperatures, and torsional shear stress *versus* square root of strain are plotted.
11. Koval, V. A., Osetski, A. I., Soldatov, V. P., and Startsev, V. I., Temperature Dependence of Creep in F.C.C. and H. C. P. Metals at Low Temperature, Advances in Cryogenic Engineering, Vol. 24, Plenum, NY (1978) pp. 249–255.
†42Curves are given for strain *versus* time (creep) at low temperatures for Ag and Al, Cu, Cd, and Zn. Low-temperature strain *∊* is related to a temperature-dependent coefficient *α* : *∊* = *α* ln (γIt+1). The temperature dependence of *α* is shown for temperatures from 1.2 K to 80 K. Creep strain *versus* log time is plotted for constant stress and different (undefined) temperatures. In the authors’ Fig. 1, creep is plotted as functions of both time and log(-time), but corresponding plots are not related by just a single mapping (linear to logarithmic). Careful study raises many unanswered questions, so the reader is cautioned to be careful in using the information from Fig. 1.
12. McClintock, R. M., and Gibbons, H. P., Mechanical Properties of Structural Materials at Low Temperatures: A Compilation from the Literature; NBS Monograph 13, Natl. Bur. Stand. (U.S.) (1960).

**Table:** For silver, annealed at 1073 K:

Test temperature	Tensile strength		Percent elongation: [gage length 20 mm (0.79 in)]
(K)	(MPa)	(psi)	
90	281	40 700	38.0
300	213	30 900	23.0
13. McKeown, J., and Hudson, O. F., Stress-strain Characteristics of Cu, Ag, and Au, J. Inst. Metals **60**, 109–132 (1937).
†36Pure Au and pure Ag, when fully annealed, show no proportionality of stress to strain in any part of the stress-strain diagram. The Young’s modulus *E* for silver was about 71 GPa (10.3 Mpsi) for an annealed (as received) specimen (room temperature assumed). The limit of proportionality was 17.9 MPa (1.3 ton/in^2^), and the 0.01 % proof stress was 45.5 MPa (3.3 ton/in^2^). Annealing a specimen for 1 h at 700 °C after 5 % overstrain in tension gave *E* =78 GPa (11.3 Mpsi). Tables of data for proportional limit, proof stress, maximum stress, and Young’s modulus for silver are given.
14. McLean, D., Strain Hardening in Pure Metals, Chap. 5 of Mechanical Properties of Metals, J. Wiley, NY (1962).
  Numerous plots of mechanical properties are given, including nominal stress *versus* percent elongation, true tensile stress *versus* natural elongation, normalized tensile stress (stress divided by shear modulus and melting temperature) *versus* natural elongation, strain-rate sensitivity of flow stress *versus* temperature, from 0 K to 370 K; dislocation density *versus* percent elongation, and flow stress *versus* dislocation spacing.
15. Mitchell, T. E., Progress in Applied Materials Research, Vol. 6, Heywood, London (1964) p. 119.
†43This review article discusses plastic properties of fcc metals and alloys, dislocations, and the theories of flow stress and work-hardening. The behavior of silver under applied stress is studied. Normalized flow stress ratio is plotted as a function of temperature.
16. Physical Properties of Materials: I. Strengths and Related Properties of Metals and Certain Other Engineering Materials; Circular of the Bureau of Standards, No. 101, p. 46, Natl. Bur. Stand. (U.S.) (1921).
  A few values of tensile strength are given. The temperature, unstated, is probably room temperature.
17. Ledbetter, H., Private communication, Materials Reliability Div., NIST-Boulder, 80303–3328.
†41Values for Young’s modulus *E* and for d*E*/d*T* were calculated from elastic-coefficient data.
18. Rice, M. H., McQueen, R. G., and Walsh, J. M., Compression of Solids by Strong Shock Waves, Solid State Physics: Advances in Research and Applications, Vol. 6, Seitz and Turnbull, eds., Academic, NY (1958) pp. 1–65.
  Basic relations from fluid dynamics and experimental data from shock-wave experiments are reviewed. Several equations of state are discussed. Relations between shock-wave velocity, pressure and relative volume are tabulated. For silver, pressure is plotted as a function of relative volume *V*/*V*_0_ for 10 GPa < *p* < 52 GPa (100 < *p* < 520 kbar). An analytical fit to a Hugoniot curve *p* = *Aμ* + *Bμ*^2^ + *Cμ*^3^ (*p* in kbar, *μ* = [*ρ*/*ρ*_0_]−1), gave *A* = 1088, *B* = 2687 and *C* = 2520. An analytical fit to the volume dependence of the Grüneisen ratio *γ* = [*V*(∂*P*/∂*T*)_v_]/*C*_v_ = *γ*_0_ + *Aμ* + *Bμ*^−^ + *Cμ*^3^ gave for silver: *γ*_0_ = 2.47, *A* = −5.670, *B* = 19.334 and *C* = 32.891.
19. Shaw, C. W., Shepard, L. A., and Wulff, J., Plastic Deformation of Thin Brazed Joints in Shear, Report to Office of Scientific Research, Air Research and Development Command (ARDC), USAF, under Contract No. AF 49(638)-775, Project No. 9782, Task No. 37718, November 1962.
  Stress-strain curves for thin silver joints brazed in tubular steel specimens, at large strains, and shear stress under plastic strain, at room temperature, are plotted. Both the yield strength and the work hardening rate were increased by the presence of the rigid interfaces.
20. Tenbrink, J., Wilhelm, M., Heine, K., and Krauth, H., Development of Technical High-*T*_C_ Superconductor Wires and Tapes, IEEE Trans. Appl. Super-cond. **3**(1), 1123–1126 (1993).
  Yield strength and critical current densities are plotted for several high-*T*_C_ wires sheathed with dispersion-hardened silver alloy. The silver sheath material improves the mechanical properties and resistance to thermal shock. Vickers hardnesses of pure cold-worked and annealed silver are compared to those of two AgNiMg alloys.
21. Wigley, D. A., Deformation Processes in Pure Metals, Chap. 1 in Mechanical Properties of Materials at Low Temperatures, Plenum, NY (1971).
  For polycrystals of Ag (and Cu), strain *∊* depends linearly on dislocation density *ρ* : *ρ* ≈ 2×10^11^*∊*. Flow stress ratio (ratio of flow stress to shear stress, normalized to unity at 0 K) is plotted for Ag, Cu, Ni and Al: from 0 K to 350 K (Ni), 410 K (Ag) and 600 K (Cu and Al). Tensile strength (stress) = 373 MPa and elastic modulus (*E*) = 83.8 MPa for Ag at 0 K.

#### 3.8.1 Fatigue

1. Hashimoto, S., Miura, S., and Yagi, T., Decrease in Dislocation Density During Reverse Deformation in Silver Single Crystals by Etch-Pit Method, Scripta Metall. **10**(9), 823–27 (1976).
  Reports the changes in the distribution and density of dislocations in single crystals of silver (purities: series 1, 99.99 %; series 2, 99.999 %) subjected to stress reversal at small strains. The dislocation density was reduced from 10^6^/cm^2^ to the order of 10^4^ to 10^5^/cm^2^ by thermal cyclic annealing in vacuum for about ten days. Pictures of etch-pit configurations are shown. One plot of shear stress (in compression, then in tension, then again in compression) versus cumulative shear strain is given (Spec. 1-1) for values of strain up to about 1.3 %.
2. McCammon, R. D., and Rosenberg, H. M., The fatigue and ultimate tensile strengths of metals between 4.2 and 293 K; Proc. Roy. Soc. (London) **A242**, 203–211 (1957).
†33 †34 †44The fatigue of Ag, Al, Cu, and Au has been measured at 4 K, 20 K, 90 K and 293 K as a function of number of cycles to fracture. The fatigue strength improves considerably as the temperature is reduced. The ultimate tensile strength is given as a function of temperature, and is also shown normalized by the fatigue strength.

#### 3.8.2 Tensile Strength

1. Carreker, R. P. Jr., Tensile Deformation of Silver as a Function of Temperature, Strain Rate, and Grain Size; Trans. AIME, J. Metals **7**, 112–115 (1957).
  Yield strength, tensile strength, percent elongation, true stress, true strain, flow stress and strain-rate sensitivity are reported for annealed silver of three grain sizes and “0.9997 purity,” over the range 20 to 1173 K. Correlations are also given with grain size.

McCammon, R. D., and Rosenberg, H. M., The fatigue and ultimate tensile strengths of metals between 4.2 K and 293 K, Proc. Roy. Soc. (London) **A242**, 203–211 (1957). See Ref. 140, Sec. **3.8.1 Fatigue**.

### 3.9 Optical and High-Frequency Properties

1. F. Abeles, Ed., Colloquium on Optical Properties and Electronic Structure of Metals and Alloys, North-Holland/J. Wiley, NY (1966).
  For properties of silver, there are plots of infrared (3 μm to 30 μm) reflectance of silver deposited under ultra-high vacuum” conditions (Bennett and Bennett, pp. 175–188); ultraviolet reflectivity for photons of energy 1 eV to 45 eV, and real and imaginary dielectric constants, *ϵ*_1_ and *ϵ*_2_ (Mme. Robin, pp. 202–209); of density of states versus energy (Spicer, pp. 296–314); and of PRFE rotation, χ, and ellipticity, *Q*, for pure silver (Stern, pp. 599–612).
2. Brandenberg, W. M., The Reflectivity of Solids at Grazing Angles, Measurement of Thermal Radiation Properties of Solids, NASA SP-31, Joseph C. Richmond, ed., NASA, Wash., DC (1963) pp. 75–82.
†20An integrating-sphere reflectometer is described for measurement of reflectivity of imperfectly diffuse specimens. Correct operation was checked by measurements on black glass and a platinum mirror. Reflectivities of Ag, Al, Au, and stainless steel were measured and graphed for angles from 0° to 90°.
3. Cline, David, Infrared Wavelength Dependence of the Total Absorptivity of Electroplated Silver; J. Appl. Phys. **33**(7), 2310–2311 (1962).
  The total hemispherical absorptivity of electroplated silver at 75.8 K was measured as a function of blackbody-radiation (BBR) temperatures from 268 K to 367 K. Over this range the total hemispherical absorptivity decreases with increasing BBR temperature. The normal absorptivity increases with increasing mean wavelength.
4. Corruccini, R. J., Thermal Radiation Properties of Solids at Low Temperatures, Measurement of Thermal Radiation Properties of Solids, Joseph C. Rich-mond, ed., NASA SP-31, NASA, Wash., DC (1963) pp. 33–37.
  In an introduction to a symposium session, the thermal radiative properties of solids at 0 K < *T* < 200 K, together with the experimental methods used to obtain such properties, are briefly reviewed. A short but wide-ranging bibliography is included.
5. Dickson, P. F., and Jones, M. C., Infrared Reflectances of Metals at Cryogenic Temperatures—A Compilation from the Literature, NBS Tech. Note 348, Natl. Bur. Stand. (U.S.) (1966).
  Spectral and total reflectances for 11 metals and alloys, including silver, are given.
6. Fulk, M. M., and Reynolds, M. M., Emissivities of Metallic Surfaces at 76 K, J. Appl. Phys. **28**(12), 1464–1467 (1957).
  The total hemispherical emissivities (THE) of 51 specimens of commercially available metals or metals of technical grade were measured by radiative heat transport between surfaces at 300 K and 76 K. The THE of silver plated on copper was 0.017 K at 76 K and 0.013 K at 20 K. An unbuffed silver-plated surface (“careful preparation”; nickel and copper strike on stainless steel) had the lowest THE (0.007). For best results electroplated surfaces should be of high purity, dense, and fairly thick. Buffing a surface does not help; the absorptivities of some surfaces increase if they are mechanically polished.
7. Parker Givens, M., Optical Properties of Metals, Solid State Physics: Advances in Research and Applications, Vol. 6, Seitz and Turnbull, eds., Academic, NY (1958) pp. 313–352.
  Classical electromagnetic theory is reviewed, along with the optical properties of noble, alkali and divalent metals, Al, and liquid metals. For silver, dispersion relations: conductivity (*nk*ν), (1−*ϵ*) = (*n–k*^2^) and the optical “constants” *n* and *k* are plotted as functions of wavelength for 0.2 μm < λ < 1.5 μm.
8. Sokolov, A. V., Optical Properties of Metals, transl. by S. Chomet, American Elsevier, NY, (1961).
  This advanced textbook reviews the macroscopic theory, the classical electron theory, and the quantum theory of the optical properties of metals, including the anomalous skin effect. The theoretical value for the beginning of the absorption band in silver is 4.7×10^−5^ cm; the experimental value is 3.1×10^−5^. At optical frequencies (*λ* = 600 nm) the effective conductivity σ is 4×10^13^ s^−1^. The permittivity *ϵ* at low frequencies is −3×10^5^. The effective electron mass is 0.97 *m*_e_. The absorptive powers between 1.5 μm and 3.3 μm and at 4.2 K are: surface, 0.0036; volume, 0.0009; total, 0.0045; experimental (total), 0.0044. Dingle’s dispersion relations (Table 11, p. 245) are (*k*^2^−*n*^2^)/*λ*^2^ = 64.2×10^8^ cm^−2^, *nk*/*λ*^3^ = 1.05×10^12^ cm^−3^; effective conductivity σ = 61×10^16^ esu, density *N* of free electrons is 7.2×10^22^ cm^−3^, and the relaxation time τ is 3.36×10^−14^ s.
9. Touloukian, Y. S., and DeWitt, D. P., eds., Thermophysical Properties of Matter: The TPRC Data Series, Vol. 7, Thermal Radiant Properties, Plenum, NY (1970).
  Data are tabulated for emittances (hemispherical total; normal total; normal spectral), reflectances (normal spectral; angular spectral, absorptances (hemispherical integrated; normal spectral; angular spectral; normal solar), and transmittance (normal spectral). For most of the quantities indicated above the data are also plotted. Because of the paucity of data for silver, no recommended values are given.

#### 3.9.1 Skin Effect

1. Chambers, R. G., Anomalous Skin Effect in Metals, Nature **165**, 239–240 (1950).
  Gives a value of 9.0×10^−10^ (Ω·cm^2^)^−1^ for the ratio of conductivity to mean-free-path (σ_el_/*λ*), and 0.66 for the effective number of conduction electrons per atom; plots reflection coefficient vs *α*^1/6^, where *α* = 3*λ*^2^(2δ^2^), *λ* is the mean free path, and *δ* is the classical skin depth.
2. Dingle, R. B., The Anomalous Skin Effect and the Reflectivity of Metals, Physica **19**, 348–364 (1953).
  The theory of the optical properties of metals in the infrared agrees much better with experimental data when the anomalous skin effect is considered, and diffuse, rather than specular, reflection of electrons at the metallic surface is assumed. Data for absorptivity at normal incidence at 17 °C and −188 °C are given for silver foil 14 μm thick.
3. Sondheimer, E. H., The Mean Free Path of Electrons in Metals, Adv. Phys. **1**, 1–42 (1952).
  Absorption of EM radiation by silver at LHe temperatures is treated.

### 3.10 Proximity Effect (Superconductive)

1. Deutscher, G., Hsieh, S. Y., Lindenfeld, P., and Wolf, S., Superconductivity in Silver Induced by the Proximity Effect, Phys. Rev. **B8**(11), 5055–5064 (1973).
  A layer of superconductivity has been induced in multiple-layer thin-film specimens of silver on a superconducting lead-bismuth alloy by a proximity effect. The alloy was made with a mole ratio of 5 parts Bi to 100 parts Pb+Bi. A layer of Pb-Bi alloy, 100 nm thick, was deposited on specimens of Ag ranging in thickness from 53 nm to 200 nm. For triple-layer specimens, 2 layers of 200 nm Pb-Bi alloy were sandwiched with specimens of Ag ranging from 80 nm to 500 nm thick. The energy gap at 0 K is *e*_0_ = 1.76 *k*_B_·*T*_c_; *T*_c_ is not explicitly defined but seems to be the superconducting transition temperature for the substrate.

### 3.11 Thermal Conductivity

(See also **3.6.4 Lorenz Number**, and **3.15 Transport Properties**)

1. Borchi, E., De Gennaro, S., Pelosi, G., Rettori, A., and Tasselli, P. L., Electronic contribution to the thermal conductivity of noble metals in the low-temperature region (*T*<15), Phys. Rev. **B19**(12), 6260–6266 (1979).
  By a variational method, for two different pseudo-potentials (Moriarty; Nand et al.), the electronic contributions to the low-temperature thermal conductivities of Ag and Cu were calculated. The temperature dependences of thermal resistivity are plotted for Ag and Cu for temperatures from 1.5 K to 15 K.
2. Cezairliyan, A., and Touloukian, Y. S., Correlation and Prediction of Thermal Conductivity of Metals Through the Application of the Principle of Corresponding States, Advances in Thermophysical Properties at Extreme Temperatures and Pressures, Serge Gratch, ed., ASME, NY (1965) pp. 301–313.
  Values for thermal conductivities of 22 metals were shown to be correlated by universal relations based on reduced values k* and T* of thermal conductivity and temperature. For cryogenic temperatures the reduced values are defined by k* = *k*/*k*_m_ and T* = *T*/*T*_m_, where *k*_m_ is the maximum value of conductivity and *T*_m_ is the corresponding value of temperature. For moderate and high temperatures, the reduced values are defined by T* = *T*/*T*_Θ_ and k* = *k*/*k*Θ, where *T*_Θ_ is the Debye characteristic temperature and *k*_Θ_ is the thermal conductivity at the Debye temperature. Recommended values for thermal conductivities of Ag, Au, and Cu are plotted as functions of temperature and of an impurity parameter *β*. This parameter is defined by *β* = *ρ*_0_/*L*, where *ρ*_0_ is the residual electrical resistivity and *L* is the Lorenz number, 2.45×10^−8^ V^2^/K^2^.
3. Childs, G. E., Ericks, L. J., and Powell, R. L., Thermal Conductivity of Solids At Room Temperature and Below: A Review and Compilation of the Literature, Natl. Bur. Stand. (U.S.) (1973) pp. 553–554.
  Thermal conductivities of Ag (99.999 % pure; *RRR*=115), Au and Cu are plotted for temperatures from 0.2 K to 1 K.
  Crisp, R. S., and Rungis, J., Thermoelectric Power and Thermal Conductivity in the Silver-Gold Alloy System from 3–300 K, Phil. Mag. **22**(176), 217 (1970), see Ref. 196, Sec. **3.1 Thermoelectric Properties**.
4. Ehrlich, A. C., and Schriempf, J. T., The Temperature Dependent Thermal and Electrical Resistivity of High Purity Silver From 2 to 20 K, Solid State Com-mun. **14**, 469–473 (1974), see Ref. 50, Sec. **3.6 Electrical Resistivity**.
  Fletcher, R., The Nernst-Ettinghausen coefficient of silver over a wide temperature range, J. Phys. F: Metal Phys. **1**, 821–827 (1971), see Ref. 213, Sec. **3.15.1 Nernst-Ettinghausen Effect**.
5. Gerritsen, A. N., and Linde, J. O., Thermal Conductivity of Some Dilute Silver Alloys; Physica **22**, 821–831 (1956).
  Thermal conductivities were measured for pure silver and dilute alloys of silver with Mn, In, and Pb. The thermal resistivity was found to increase faster than linearly with small concentrations of impurity. A plot is given for thermal conductivity from 1 K to 90 K for one specimen of pure silver at three stages of annealing and for one specimen of very pure silver from G. K. White (Ref. 171).
6. Gray, James H., On a Method of Determining the Thermal Conductivity of Metals, with Applications to Copper, Silver, Gold, and Platinum, Phil. Trans. Roy. Soc. (London) **A186**, 165–186 (1895).
  The thermal conductivity was determined by a calorimetric method. The data are given as mean conductivities for temperature differences over the range from 10 °C to 97 °C or over the range from 15 °C to 98 °C.
7. Ho, C. Y., Powell, R. W., and Liley, P. E., Thermal Conductivity of the Elements: A Comprehensive Review, J. Phys. Chem. Ref. Data **3**, Suppl. 1, 606–615 (1974).
†8Recommended values for the thermal conductivity of silver are tabulated and plotted (both linear and logarithmic scales) for temperatures from 0 K to 1000 K; provisional (extrapolated or estimated) values from 1073.2 K to 7273 K are also given.
  Jha, D., and Jericho, M. H., Low-Temperature Transport Properties of Dilute Silver-Manganese Alloys, Phys. Rev. **B3**(1), 147–156 (1971), see Ref. 208, Sec. **3.15 Transport Properties**
8. Kannuluik, W. G., The Thermal and Electrical Conductivities of Several Metals Between −183 and 100 °C, Proc. Roy. Soc. (London) **A141**, 159–168 (1931).
  Resistivity, thermal conductivity, and Lorenz numbers of Ag, W, Mo, and Fe at temperatures of −183 °C, −78.5 °C, 0 °C, and 100 °C are measured and tabulated.
9. Klemens, P. G., Thermal Conductivity of Solids at Low Temperatures, Encyclopedia of Physics, Vol. XIV: Low Temperature Physics I, Springer, Berlin (1956) pp. 198–279.
  This work reviews the theory of thermal conductivity of dielectric solids, of metals and alloys (both electronic and lattice components), and of superconductors.
  Kos, J. F., Relation between the Low-Temperature Thermal and Electrical Resistivities of Ag, Phys. Rev. Lett. **31**(21), 1314–1317 (1973), see Ref. 60, Sec. **3.6 Electrical Resistivity**.
  Kos, J. F., Anomalies in the low-temperature thermal and electrical resistivities of silver, J. Phys.: Condens. Matter **2**, 4859–4868 (1990), see Ref. 61, Sec. **3.6 Electrical Resistivity**.
  Kus, Fred W., Some transport properties of dilute copper and silver alloys, J. Phys. F: Metal Phys. **8**, 1483–89 (1978), see Ref. 209, Sec. **3.15 Transport Properties**.
  Laubitz, M. J., Transport properties of pure metals at high temperatures. II. Silver and Gold, Can. J. Phys. **47**, 2633–2644 (1969), see Ref. 210, Sec. **3.15 Transport Properties**.
10. Lees, Charles H., The Effects of Temperature and Pressure on the Thermal Conductivities of Solids—Part II. The Effects of Low Temperatures on the Thermal and Electrical Conductivities of Certain Approximately Pure Metals and Alloys, Phil. Trans. Roy. Soc. (London) **A208**, 381–443 (1908).
  The thermal and electrical conductivities and Lorenz ratio are tabulated as functions of temperature.
11. Matsumura, T., and Laubitz, M. J., Thermal conductivity and electrical resistivity of pure silver between 80 and 350 K, Can. J. Phys. **48**(12), 1499–1503 (1970).
  Precise measurements of the thermal conductivity and electrical resistivity of high-purity (*RRR*=1050) silver are reported for the range of temperatures from 80 K to 350 K. Values reported here agree closely with measurements by White and Woods (Ref. 76). A dip in the ideal Wiede-mann-Franz ratio for silver has not been found for Au or Cu; no explanation for the dip is offered by the authors.
12. Mendelssohn, K., and Rosenberg, H. M., The Thermal Conductivity of Metals at Low Temperatures I: The elements of Groups 1, 2 and 3, Proc. Phys. Soc. **65A**, 385–88 (1952),—II: The Transition Elements, ibid., pp. 388–394.
  The thermal conductivity of silver (99.99 % pure) was measured in the range 2 K to 40 K. An equation is given for the thermal resistance *R* = *αT*^2^+*β*/*T*, with the electron-phonon scattering term *α* = 9.0×10^−5^ and the impurity-scattering term *β* = 1.6 (conductivity units of W/(cm·K)). A conductivity maximum of approximately 9 W/(cm·K) is observed at 20 K. The maximum is rather broad, possibly indicating the presence of a large impurity scattering term.
13. Ross, R. G., Andersson, P., Sundqvist, B., and Backstrom, G., Thermal conductivity of solids and liquids under pressure, Rep. Prog. Phys. **47**, 1347–1402 (1984).
  The pressure and density dependences of the thermal conductivities of Ag, Al, Au, Cu, Fe, Ni, Pb, and Sn are tabulated.
  Rumbo, E. R., Transport Properties of Very Pure Copper and Silver Below 8.5 K, J. Phys. F: Metal Phys. **6**(1), 85–98 (1976), See Ref. 211, Sec. **3.15 Transport Properties**.
14. Sharma, J. K. N., Heat Conductivities Below 1 °K; Cryogenics **7**(6), 141–156 (1967).
  In an investigation of anomalies in expected temperature dependences, the thermal conductivities of ten very pure metals (Ag, Al, Au, Cd, Ni, Pd, Pt, Re, Ta and W) and of sapphire were measured. The thermal conductivity of silver having *RRR* = 115 is plotted for the temperature range 0.4 K < *T* < 1.0 K, and compared to results predicted from electrical resistivity measurements. The conductivity has two linear regions separated by a slight peak at 0.65 K.
15. Touloukian, Y., Powell, R. W., Ho, C. Y., and Klemens, P., eds., Thermophysical Properties of Matter: The TPRC Data Series, Vol. 2, Thermal Conductivity: Metallic Elements and Alloys, Plenum, NY (1970) pp. 340–347.
  Sixty-two sets of data for silver from the literature are tabulated and plotted (log conductivity versus log temperature) for 2 K < *T* < 920 K.
16. Van Baarle, C., Roest, G. J., Roest-Young, Mrs. M. K., and Gorter, F. W., Thermal Conductivity and Thermopower Silver-base Alloys at Low Temperatures: I. Pure Silver; Physica **32**(7), 1700–1708 (1966), —III. Alloys with Gold, Antimony and Palladium, Physica **35**, 223–240 (1967).
  Article I, pure silver, covers transport properties of 99.9999 % pure silver. Deviations from Matthiessen’s Rule are discussed. The ideal thermal resistivity of silver is plotted for 4 K < *T* < 29 K. The thermoelectric power of 6 specimens of pure silver is plotted for temperatures from 2 K to 10 K.
17. White, G. K., Thermal Conductivity of Silver at Low Temperatures, Proc. Phys. Soc. (London) **A66**, 844–845 (1953).
  The thermal conductivity and “ideal” thermal resistance of silver are plotted for temperatures from 1 K to 140 K. The ideal resistance *R*_i_ is given by *R*_i_ = 2.86×10^−5^ *T*^2·3^ (unannealed) and *R*_i_ = 1.06×10^−5^·*T*^2.5^ (annealed).

White, G. K., and Woods, S. B., Electrical and Thermal Resistivity of the Transition Elements at Low Temperatures, Phil. Trans. Roy. Soc. London **A251**(995), 273–302 (1959), See Ref. 76, Sec. **3.6 Electrical Resistivity**.

#### 3.11.1 Magnetothermal Resistance

1. Mendelssohn, K., and Rosenberg, H. M., The Thermal Conductivity of Metals in High Magnetic Fields at Low Temperatures, Proc. Roy. Soc. (London) **A218**, 190–205 (1953).
†9The thermal resistances of 27 pure metals were measured in magnetic fields up to 0.4 T. Ag (polycrystalline; purities of 99.99 % and 99.999 %), Cd, Ga, In, Pb, Sn, Tl and Zn showed appreciable magnetothermal resistance (MTR). The effect was always greater for transverse applied fields. The MTR increased quadratically with temperature at very small fields, and then rose linearly in larger fields. The ratio of *W*_H_ (thermal resistance in magnetic field, *H*) versus *W*_0_ (thermal resistance in zero field) is plotted versus applied magnetic field.
2. van Witzenburg, W., and Laubitz, M. J., Magne-toresistances and the Phonon Conductivity of Metals, Can. J. Phys. **46**, 1887–1894 (1968).
  Electrical and thermal magnetoresistances were determined at a maximum field of 4.46 T, over the range 80 K < *T* < 130 K. Results were interpreted in terms of “normal” and “magnetic” Wiedemann-Franz ratios for Ag, Au, and Cu. These “normal” and “magnetic” ratios were plotted. Parameters for the phonon conductivity are tabulated. Results were applied to data for conductivity of tungsten.

#### 3.11.2 Thermal Contact Resistance

1. Mamiya, T., Yano, H., Uchiyama, T., Inoue, S., and Miura, Y., Thermal contact of joints between different kinds of metals at low temperatures, Rev. Sci. In-strum. **59**(8), 1428 (1988).
  Data for contact resistance of an electron-beam weld, as measured by electrical resistance, of silver to silver are given for measurements at 4.2 K; information on electrical resistance of a threaded contact between Ag and Pt is also given. Actual thermal contact resistance, in terms of temperature difference, is not given.

#### 3.11.3 Thermal Diffusivity

1. Touloukian, Y. S., Powell, R. W., Ho, C. Y., and Nicolaou, M. C., eds., Thermophysical Properties of Matter: The TPRC Data Series, Vol. 10, Thermal Diffusivity, Plenum, NY (1973) p. 164.
†10Recommended values of diffusivity *α* (for both solid and liquid) are tabulated and the corresponding curve plotted for 4 K < *T* < 7000 K.

### 3.12 Thermal Expansion

1. Dorsey, H. G., Coefficient of Linear Expansion at Low Temperatures, Phys. Rev. **25**, 88–102 (1907).
  Thermal expansion coefficients of twelve materials, including Ag, measured from 103 K to 283 K, are tabulated and graphed.
2. Fraser, D. B., and Hollis Hallett, A. C., The Coefficient of Thermal Expansion of Various Cubic Metals below 100 K, Can J. Phys. **43**, 193–219 (1965).
  The temperature dependences of dilatation *δ* and coefficient of linear thermal expansion, *α*, are tabulated over the range 17 K to 100 K for Ag, Al, Au, Cu, Fe, and Ni; some data are plotted (but not for Ag). The Grüneisen constant *γ* is plotted as a function of reduced temperature *T*/Θ_D_.
3. Kos, J. F. and Lamarche, J. L. G., Thermal Expansion of the Noble Metals below 15 K, Can J. Phys. **47**, 2509–2518 (1969).
  The thermal expansions of Ag, Au, and Cu were measured over the range 4.2 K < *T* < 15 K; data are tabulated and graphed.
4. Leksina, I. E., and Novikova, S. I., Thermal Expansion of Copper, Silver, and Gold Within a Wide Range of Temperatures, Sov. Phys.—Solid State **5**(4), 798–801 (1963).
  The thermal expansion coefficients of Cu, Ag, and Au were measured from 20 K to 1200 K, the Grüneisen constant, *γ*, was calculated from the data. The difference between the experimental and theoretical values of *γ* is graphed. The values of *γ* near the Debye temperature are: Cu, 2.0; Ag, 2.4 and Au, 3.0. The energies of formation of vacancies are: Cu, 12.41; Ag, 11.76; and Au, 54.22 kJ/mol (12.96 kcal/mol).
5. McLean, K. O., Swenson, C. A., and Case, C. R., Thermal Expansion of Copper, Silver and Gold Below 30 K, J. Low Temp. Phys. **7**, 77–98 (1972).
  Linear thermal expansion coefficients were determined for 4 K< *T*< 30 K; Grüneisen parameters *γ* are given.
6. Touloukian, Y. S., Kirby, R. K., Taylor R. E., and Desai, P. D., eds., Thermophysical Properties of Matter: The TPRC Data Series, Vol. 12, Thermal Expansion: Metallic Elements and Alloys, Plenum, NY (1975) pp. 298–299.
†11 †22Literature values for thermal linear expansion of pure metallic elements and metallic alloys are critically evaluated. Recommended values are plotted and tabulated.
7. White, G. K., and Collins, J. G., Thermal Expansion of Copper, Silver and Gold at Low Temperatures, J. Low Temp. Phys. **7**, 43–75 (1972).
  Thermal expansion coefficients, lattice contributions, and lattice Grüneisen parameters are tabulated and plotted from 2 K to about 30 K.

### 3.13 Thermodynamic Properties

1. Furukawa, G. T., Saba, W. G., and Reilly, M. L., Critical Analysis of the Heat-Capacity Data of the Literature and Evaluation of Thermodynamic Properties of Copper, Silver, and Gold from 0 to 300 K, National Standard Reference Data Series—18 Natl. Bur. Stand. (U.S.) 1968).
†25 †26 †27 †28 †31 †T8Published literature is comprehensively and critically reviewed, and recommended values of specific heats are tabulated and graphed for Ag, Au, and Cu. Also, values for entropy *S*, enthalpy *H*°*_T_*−*H*°_0_, enthalpy function (*H*°*_T_*−*H*°_0_)/*T*, Gibbs energy (*G*°*_T_*−*H*°_0_), and Gibbs energy function −(*G*°*_T_*−*H*°_0_)/*T* are tabulated (*H*°*_T_* is the “reference” enthalpy at temperature *T* and standard pressure). Debye temperature is plotted over the range of temperature from 0 K to 300 K.
2. Hultgren, R., Orr, R. L., Anderson, P. D., and Kelley, K. K., Selected Values of Thermodynamic Properties of Metals and Alloys, J. Wiley, NY (1963).
  A value of electronic specific heat coefficient *γ* = 0.615 mJ/(mol·K) (1.47×10^−4^ cal/(mol·K)) is given. Data for specific heat are tabulated for temperatures over the range 1 K to 298 K; data for specific heat, enthalpy, entropy, and free energy function −(*F_T_*−*H_ST_*)/*T* are tabulated for temperatures from 298 K to 2500 K.
3. Mott, N. F., The Cohesive Forces in Metals and Alloys, Rept. Prog. Phys. **25**, 218 (1964).
  Theory and experimental data are reviewed; it is concluded that the energy of an atom in a pure metal or alloy depends, to a first approximation, on the volume available to the atom, rather than on coordination number or nature of surroundings (in an alloy). For the cohesive energy of silver, an experimental value, 285 kJ/mol (68 kcal/mol), from the literature is compared to a theoretical value, 230 kJ/mol (55 kcal/mol).
4. Wicks, C. E., and Block, F. E., Thermodynamic Properties of 65 Elements—Their Oxides, Halides, Carbides and Nitrides, Bulletin 605, Bureau of Mines, U.S. Dept. Interior, Wash., DC (1963).
  Entropy, enthalpy, and free energy (*F* =*H*−*TS*) are tabulated as a function of temperature for silver for 298 K < T < 2000 K; similar data are given for crystalline Ag_2_0, Ag_2_0_2_, AgF, AgF_2_, and AgCl.

#### 3.13.1 Debye Temperature

1. Herbstein, F. H., Methods of Measuring Debye Temperatures and Comparison of Results for some Cubic Crystals, Adv. Phys. **10**, 313–355 (1961).
  Values of Debye temperature Θ_D_ from different methods of measurement (entropy, specific heat, elastic constants, x-ray diffraction) are given. Values of Θ_D_ obtained from entropy, specific heat, elastic constants, and diffraction measurements are tabulated for Ag, Al, Au, Cu, Ni, Pb, Pt, and Th for temperatures of 0 K and 300 K. The variation with temperature from 0 K to 1200 K of Θ_D_ for Ag is plotted.
  Martin, Douglas L., Specific Heats of Copper, Silver, and Gold below 30 °K, Phys. Rev. **141**(2), 576–582 (1966), see Ref. 191 below, Sec. **3.13.2 Specific Heat**.
  Neighbours, J. R., and Alers, G. A., Elastic Constants of Silver and Gold; Phys. Rev. **111**(3), 707–712 (1958), see Ref. 36, Sec. **3.5 Elastic Moduli.**
2. Prasad, B., and Srivastava, R. S., Lattice Dynamics of Noble Metals, J. Phys. F: Metal Phys. **2**, 247 (1972).
  Debye temperature Θ_D_ of silver as a function of temperature was calculated and compared with values measured by Giauque and Meads, J. Am. Chem. Soc. **63**, 1897 (1941). Seven points are graphed from 20 K to 122 K.

#### 3.13.2 Specific Heat

1. Corak, W. S., Garfunkel, M. P., Satterthwaite, C. B., and Wexler, A., Atomic Heats of Copper, Silver, and Gold from 1 K to 5 K, Phys. Rev. **98**(6), 1699–1707 (1955).
  Plots of atomic (specific) heats of Cu, Ag (“99.98 % purity”) and Au are given for the range 1 K < *T* < 5 K. From these data the electronic specific heat, *γ*, and (low-temperature, calorimetric) Debye temperature, Θ_D_, are determined and compared with other work. They give *γ* = 0.610 mJ/(mol·K) and Θ_D_ = 225.3 K. The authors found evidence for errors in the 1948 helium vapor-pressure-temperature scale, already suggested by earlier work; their values of *γ* and Θ_D_ have been corrected by removal of the effect of the temperature-scale errors.
  Furukawa, G. T., Saba, W. G., and Reilly, M. L., Critical Analysis of the Heat-Capacity Data of the Literature and Evaluation of Thermodynamic Properties of Copper, Silver, and Gold from 0 to 300 K, National Standard Reference Data Series—18 Natl. Bur. Stand. (U.S.) Wash., DC (1968). See Ref. 183, Sec. **3.13 Thermodynamic Properties**.
2. Keesom, P. H., and Pearlman, N., Low Temperature Heat Capacity of Solids, Encyclopedia of Physics, Vol. XIV: Low Temperature Physics I, Springer, Berlin (1956) pp. 282–344.
  This work reviews the theory of, and experimental techniques for, measuring specific heats and analyzes data in terms of effects of superconductivity, excitation modes, cooperative phenomena and size effects.
3. Martin, Douglas L., Specific Heats of Copper, Silver, and Gold below 30 °K, Phys. Rev. **141**(2), 576–582 (1966).
†23 †24 †29 †T7The specific heats of pure Ag, Cu, and Au were measured over the range 3 K < T < 30 K. Specific heat data for all three metals are tabulated and plotted. For silver the estimated values of the De-bye temperature at zero temperature Θ_0_ and electronic specific-heat coefficient *γ* are: Θ_0_ = 226.6 K and *γ* = 640.9 μJ/(K^2^·mol).
4. Martin, Douglas L., Specific Heats below 3 °K of Pure Copper, Silver, and Gold, and of Extremely Dilute Gold-Transition-Metal Alloys, Phys. Rev. **170**(3), 650–655 (1968), erratum: Phys. Rev. **174**(3), 1082 (1968).
  Use of an improved apparatus allowed more accurate values for the limiting low-temperature Debye temperatures Θ_0_ and electronic specific-heat coefficients *γ* to be obtained for Ag, Au, and Cu. The coefficient of the *T*^5^ term in the expansion representing the lattice component of specific heat is negligible for Ag (and for Cu). For *T* < 3.0 K the specific heat *C* is well represented by *C* = *γT* + 1943.8/Θ_0_^2^)·*T*^3^ (units of μJ/(K·mol)). The estimated values for silver are: Θ_0_ = 227.3 K and *γ* = 640.9 μJ/(K^2^·g-mol). The effective mass m* of an electron in silver is 1.01 *m*_e_ (*m*_e_ is the free-electron mass).
5. Parkinson, D. H., The Specific Heats of Metals at Low Temperatures, Rept. Prog. Phys. **21**, 226–270 (1958).
†30The theories of the lattice and electronic specific heats are reviewed, and experimental results are given for alkali metals, noble metals, divalent and transition metals, rare-earth metals, and superconductors. The Debye temperatures of silver, obtained from experiment and three theories, are compared and plotted as a function of thermodynamic temperature from 1 K to 96 K. The effective mass m*, defined by m*/*m* = *γ* (expt)/*γ* (free electron), is 0.95. From this relation the electronic specific heat is *C*_e_ = (m*/*m*)·*γT*, where *γ* = 0.66 mJ/(mol·K^2^) for silver of “99.999 % purity.”
6. Touloukian, Y., and Buyco, E. H., eds., Thermophysical Properties of Matter, The TPRC Data Series, Vol. 4, Specific Heat: Metallic Elements and Alloys, Plenum, NY (1970) pp. 208–212.
  Data from the literature are critically examined. Original data are tabulated and plotted. A recommended value is plotted.

### 3.14 Thermoelectric Properties

1. Barnard, R. D., Thermoelectricity in Metals and Alloys, Taylor & Francis, London (1972).
  This general review of thermoelectricity gives a plot of absolute thermopower of silver *versus* temperature, from MacDonald (Ref. 201). MacDonald in turn got his data from W. B. Pearson (Ref. 202).
2. Crisp, R. S., and Rungis, J., Thermoelectric Power and Thermal Conductivity in the Silver-Gold Alloy System from 3–300 K, Phil. Mag. **22**(176), 217 (1970).
  This work focuses mostly on alloys but plots the lattice characteristic thermopower for Ag and Au, from 80 K to 300 K. No data are given for the thermal conductivity of pure Ag.
3. Crisp, R. S., and Henry, W. G., Thermopower in α phase alloys of Cu, Zn, Ga, Ge, and As in Ag at low temperatures, J. Phys. F: Metal Phys. **8**(8), 1767–1781 (1978).
  Thermopower of silver is plotted for 35 K < *T* < 58 K and for 175 K < *T* < 205 K.
4. Cusack, N., and Kendall, P., The Absolute Scale of Thermoelectric Power at High Temperature, Proc. Phys. Soc. **72**, 898–901 (1958).
  The absolute thermopowers of Ag, Au, Cu, Mo, Pt, Pd, and W are tabulated over the range 100 K < *T* < 2400 K (100 K to 1200 K for Ag).
  Ewbank, M., Imes, J. L., Pratt, W. P., Jr., Schroeder, P. A., and Tracy, J. M., Measurements of Transport Properties of Silver between 20 mK and 4.2 K, Phys. Lett. **59A**(4), 316–318 (1976), see Ref. 207, Sec. **3.15 Transport Properties**.
  Fletcher, R., The Nernst-Ettinghausen coefficient of silver over a wide temperature range, J. Phys. F: Metal Phys. **1**, 821–827 (1971), see Ref. 213, Sec. **3.15.1 Nernst-Ettinghausen Effect**.
5. Guenault, A. M., Themoelectric Power of Silver Alloys at very Low Temperatures, Phil. Mag. **15**, 17–25 (1967).
  The absolute thermopower, for silver with 2 ppm (parts in 10^6^) oxygen, was measured over the range 0.3 K to 5 K.
6. Guenault, A. M., and Hawksworth, D. B., Themoelectric power of the pure noble metals at low temperatures, J. Phys. Metal Phys. **7**(8), L219–222 (1977).
  Thermopower, for 6 different specimens of pure silver (as high as “99.9999 % purity”, 2 ppm oxygen), is plotted for 0.3 K < *T* < 12 K.
  Khoshenevisan, M., Pratt, W. P., Jr., Schroeder, P. A., and Steenwyk, S. D., Low-temperature resistivity and thermoelectric ratio of copper and gold, Phys. Rev. **B19**(8), 3873–3878 (1979), see Ref. 56, Sec. **3.6 Electrical Resistivity.**
7. MacDonald, D. K. C., Thermoelectricity: An Introduction to the Principles, J. Wiley, NY (1962).
  This is a general treatment of thermoelectricity.
8. Pearson, W. B., Survey of thermoelectric studies of the Group I metals at low temperatures carried out at NRL, Ottawa, Soviet Phys.—Solid State **3**(5), 1024–1033 (1961).
†12Absolute thermoelectric power *S*(*T*) is plotted over 4 K < *T* < 250 K. Gives coefficients of a linear approximation to *S* for temperatures above the Debye temperature: *S*(*T*) = 4.7·T nV/K.
9. Rumbo, E. R., Measurement of Thermoelectric Power at Low Temperatures, Phil. Mag. **19**, 689 (1969).
  The thermopower of polycrystalline (99.9999 % pure) silver is graphed for 1.2 K < *T* < 4.2 K.
10. Rumbo, E. R., Thermopower Measurements on Copper and Silver at Low Temperatures with Particular Attention to Scattering by Residual Magnetic Impurities, Phil. Mag. **22**, 953–964 (1970).
  Thermoelectric power of a pure (*RRR* = 2320) silver specimen is graphed for 2 K < *T* < 4.2 K.
  Rumbo, E. R., Transport Properties of Very Pure Copper and Silver below 8.5 K, J. Phys. F: Metal Phys. **6**(1), 85–98 (1976), see Ref. 211, Sec. **3.15 Transport Properties**.
11. Van Baarle, C., Roest, G. J., Roest-Young, M. K., and Gorter, F. W., Thermal Conductivity and Thermo-power of Silver and Silver-base Alloys at Low Temperatures: I. Pure Silver; Physica **32**(7), 1700–1708 (1966), —III. Alloys with Gold, Antimony and Palladium, Phys-ica **35**, 223–240 (1967).
  Article I covers transport properties of 99.9999 % pure silver. Deviations from Matthiessen’s Rule are discussed. The ideal thermal resistivity of silver is plotted for 4 K to 29 K. The thermoelectric power of 6 specimens of pure silver is plotted for temperatures from 2 K to 10 K.

### 3.15 Transport Properties

(See also: **3.6 Electrical Resistivity**, and **3.11 Thermal Conductivity**)

1. Geylikman, B. T., and Orlov, V. G., Low-temperature transport properties of pure noble metals, Sov. Phys. Solid State **18**(3), 380–383 (1976).
  Transport properties of pure noble metals are reviewed in terms of normal processes on the necks of their Fermi surfaces.
2. Ewbank, M., Imes, J. L., Pratt, W. P., Jr., Schroeder, P. A., and Tracy, J., Measurements of Transport Properties of Silver between 20 mK and 4.2 K, Phys. Lett. **59A**(4), 316–318 (1976).
  Thermoelectric ratio, thermopower, and electrical resistivity of high-purity as-cast and annealed silver specimens are plotted.
3. Jha, D., and Jericho, M. H., Low-Temperature Transport Properties of Dilute Silver-Manganese Alloys, Phys. Rev. **B3**(1), 147–156 (1971).
  The electrical resistivity and thermal conductivity were measured between 0.3 K and 20 K for dilute alloys of Mn in (“99.9999 % pure”) Ag. The most nearly pure alloy had a nominal mole concentration of 0.005 parts Mn to 100 parts Ag+Mn.
4. Kus, Fred W., Some transport properties of dilute copper and silver alloys, J. Phys. F: Metal Phys. **8**, 1483–89 (1978).
  Tabulates data for electrical and thermal resistivi-ties of Ag and Cu and their dilute alloys, for 10 K < *T* < 100 K. Results are discussed in terms of deviations from Matthiessen’s Rule in both the electrical and thermal resistivities.
5. Laubitz, M. J., Transport properties of pure metals at high temperatures. II. Silver and Gold, Can. J. Phys. **47**, 2633–2644 (1969).
  Measurements of transport properties (electrical resistivities and thermal conductivities) reported here cover the range from 300 K to 1100 K for Ag, and 300 K to 1200 K for Au. The computed Wiedemann-Franz ratio does not reach the theoretical value of Sommerfeld, even at the highest temperatures investigated.
6. Rumbo, E. R., Transport Properties of Very Pure Copper and Silver below 8.5 K, J. Phys. F: Metal Phys. **6**(1), 85–98 (1976).
  Thermal and electrical conductivities, and thermo-power, of very pure Ag and Cu were measured between 1 K and 8.5 K. The Ag specimen had a *RRR* of 3270 after eliminating effects of impurities by oxygen annealing. The ideal electrical resistivi-ties of both Ag and Cu were found to vary approximately as *T*^3.5^ between 8.5 K and 4 K, and as *T*^2.5^ to *T*^2.8^ below 4 K. A maximum in the thermopower of Ag was found at around 6 K. A value for the Lorenz number *L*_0_ of Ag is given: 2.46× 10^−8^(V/K)^2^.
7. Ziman, J. M., The Ordinary Transport Properties of the Noble Metals, Adv. Phys. **10**, 1–56 (1961).
  Properties of the noble metals Ag, Au, and Cu are reviewed; values are tabulated for the following quantities (numerical values given here for silver): lattice constant (at 20 °C), a = 0.4078 nm; atomic radius, *r* = 0.159 nm; Fermi energy, *E*_F_ = 5.51 eV; electronic specific heat parameter, *γ*_calc_ = 0.644 mJ/(mol·K^2^) and *γ*_obs_ = 0.610 mJ/(mol·K^2^); Debye temperature, Θ_D_ = 210 K to 225 K; the Hall constant for free electrons, *R*_F_ = 1/(*Nec*) = −10.65×10^−5^ cm^3^/C; observed Hall constant, *R*_obs_ = −8.0 to −8.5 (×10^− 5^cm^3^/C); neck radius for the Fermi surface, *r* = 0.13p; effective mass (belly) *m*_H_ = 0.7m_e_. Values for magnetoresistance, electrical and thermal conductivities, thermoelectric powers (silver: 100 K to 1200 K), and lattice thermopower are given.

#### 3.15.1 Nernst-Ettinghausen Effect

1. Fletcher, R., The Nernst-Ettinghausen coefficient of silver over a wide temperature range, J. Phys. F: Metal Phys. **1**, 821–827 (1971).
  The Nernst-Ettinghausen (NE) effect is the appearance of a transverse electric field *E*_y_ in a metallic conductor subjected to mutually perpendicular magnetic induction *B_z_* and temperature gradient ∂*T*/∂x: *E_y_* = *Q*·*B_z_*·∂*T*/∂*x*. The isothermal NE coefficient *Q^i^*, measured over the range 4 K < *T* < 200 K, shows a strong peak at about 16 K. Thermal conductivity in zero magnetic field and in *B*=0.885 *T*, and absolute thermopower of silver, are plotted for temperatures from 4 K to 78 K. Sondheimer’s two-band model is found to predict a phonon-drag contribution to the thermopower of the wrong sign.

### 3.16 Gas Solubility

1. Cupp, C. R., Gases in Metals, Prog. in Metal Phys., Vol. 4, B. Chalmers, ed., Interscience/Pergamon, NY (1953) p. 112.
  Values are given for the solubility in silver (per cm^3^ of silver) of oxygen at a pressure of 107 kPa (80 cm Hg), at 4 different temperatures:

**Table:** 

200 °C	0.142 cm^3^
400 °C	0.087 cm^3^
700 °C	0.193 cm^3^
800 °C	0.354 cm^3^
  The solubility of oxygen in silver is said to be proportional to the square root of pressure, and is a minimum at 400 °C. There is no simple relation that models the dependence on temperature. It is suggested that above 400 °C the oxygen is present as atoms.

### 3.17 Coin Silver

1. Van Degrift, Craig, Coin Silver as a Construction Material in Low-Temperature Experiments, Physica **107B**, 605–6 (1981).
  Some low-temperature properties (density, electrical resistivity, thermal conductivity and specific heat) of coin metal (90.3 % Ag, 9.7 % Cu, by mass) are tabulated. Direct measurements of magnetothermal conductivity for *T* < 20 K are included.

## Figures and Tables

**Table 1:** Physical and chemical properties of silver (Ag: argentum)

Atomic number, *Z*	47	Melting point	961.93 °C
Atomic mass, *A*	107.8682 u	Density (20 °C)	10.492 g/cm^3^
Isotopes (*A*=107)	106.9051 u (51.84 %)	(0 K)	10.63 g/cm^3^
(*A*=109)	108.9048 u (48.16%) 0.2883	Debye	226.5 K (0 K)
Atomic diameter	nm	temperature	215 K (20 °C)
Electronic structure	[Kr] 4*d*^10^ 5*s*^1^	Thermoelec. power	1.35 μV/K (20 °C)
Valence states	1, 2	Elec. resistivity	0.0147 μΩ·m (0 °C)
Crystal structure	face-centered cubic, Fm3m	Elastic constatnts (0 K)	*C*_11_ = 131.5 GPa *C*_12_ = 97.33 GPa
Lattice spacing, a_0_	0.4078 nm (20 °C)		*C*_44_ = 51.1 GPa
Electrochemical potential	0.798 V (Ag^+^+e^−^ ⇋ Ag)	Young’s modulus	82.5 GPa (293 K) 91.3 GPa (0 K)
Ionization potentials	7.574 V (I)	Poisson ratio	0.364 (293 K)
	21.960 V (II)	Hall coefficient:	
	36.10 V (III)	(290 K)	−9.0×10^−11^ m^3^/(A·s)
Cohesive energy	2.96 eV/atom,	(20 K)	−10.2×10^11^ m^3^/(A·s)
(25 °C)	285.8 kJ/mol	Fermi energy	5.52 eV = 8.84×10^−19^ J
Magnetic (mass) susceptibility	−2.300×10^−9^ m^3^/kg (4.2 K)	Fermi surface	Spherical, with necks at (111)
	−2.27×10^−9^ m^3^/kg (295 K)		

Constants: *u* = ^12^C/12 = 1.66×10^−27^ kg

1 eV = 1.602×10^−19^ J

*N*_A_ = 6.023×10^23^ molecules per mole

**Table 2a:** Conversion factors—I

K	= °C+273.15	= (°F−32)/1.8+273.15
1 J	= 0.239 cal	= 9.480×10^−4^ Btu
1 W/(m·K)	= 2.39×10^−3^ cal/(s·cm·°C)	= 0.578 Btu/h·ft·°F)
1 J/(kg·K)	= 2.39×10^−4^ cal/(g·°C)	= 2.39×10^−4^ Btu/(lb_m_·°F)
1 m^2^/s	= 10^4^ cm^2^/s	= 10.76 ft^2^/s
1 Pa	= 10 dyne/cm^2^	= 1.45×10^−4^ lb_f_/in^2^
1 T	= 10^4^G	
μ_0_	= 4π×10^−7^ H/m	
1 m^3^/kg	= 79.58 emu/g	(Magnetic susceptibility)
1 (SI)	= 7.958×10^−2^ emu/cm^3^	(Magnetic susceptibility)

**Table 2b:** Conversion factors—II

Multiply	By	To obtain
kips	453.59	kg
kg	2.20463	lb_m_
kips/in^2^	0.7031	kg/mm^2^
kg/mm^2^	1.4223	kips/in^2^
kg/mm^2^	9.080665	MPa
lb/in^3^	27.68	g/cm^3^
lb/ft^3^	0.01602	g/cm^3^
g/cm^3^	62.4219725	lb/ft^3^

**Table 3:** Residual resistivities, per atomic percent Ag for solute element, nΩ·m/(^at^/_0_)

Al	As	Au	Bi	Cd	Cu	Ga	Ge	Hg	In
19.5	84.6	3.8	73	3.82	0.68	22.8	55.2	7.9	17.8

Mg	Mn	Ni	Pb	Pd	Pt	Sb	Sn	Tl	Zn

5	16	11	46.4	4.36	15.9	72.6	43.2	22.7	6.2

Frank J. Blatt, Physics of Electronic Conduction in Solids, p. 199 (McGraw-Hill, NY, 1968): Al, Mg, Mn, Ni.

J. O. Linde, Ann. Phys. (Leipzig), 5. Folge, Band 14, 353–366 (1932): As-In, Pb-Zn.

**Table 4:** Relative resistance and relative volume of silver as functions of pressure

Pressure GPa	*R*p*/R*_0_^a^	*V*_P_/*V*_0_^b^
0.00	1.000	1.00000
0.98	0.966	0.99062
1.96	0.936	0.98180
2.93	0.910	0.97381
3.91	0.885	
4.89	0.864	
5.87	0.846	
6.85	0.831	
7.82	0.819	
8.80	0.809	
9.78	0.802	

a P. W. Bridgman, Proc. Amer. Acad. Arts, Sci. **81**, 165 (1952).

b A. W. Lawson, Progress in Metal Physics, Vol. 6, Pergamon, NY (1956).

**Table 5:** Infrared absorptance of silver

Freshly evaporated film^a^		Bulk metal^b^		Chemically deposited: polished surface^c^	

wave-length μm	absorptance	wave-length μm	absorptance	wave-length μm	Absorptance
0.220	0.720	0.45	0.12	0.251	0.659
0.240	0.705	0.50	0.10	0.288	0.788
0.250	0.696	0.55	0.085	0.305	0.909
0.260	0.708	0.60	0.073	0.326	0.854
0.280	0.748	0.65	0.065	0.357	0.255
0.300	0.824	0.70	0.059	0.385	0.186
0.315	0.945	0.75	0.053	0.420	0.134
0.320	0.911	0.80	0.049	0.450	0.095
0.340	0.271	0.90	0.040	0.500	0.087
0.360	0.118	0.95	0.037	0.550	0.073
0.380	0.072	1.00	0.035	0.600	0.074
0.400	0.052	1.05	0.033	0.650	0.053
0.450	0.034	1.10	0.031	0.700	0.046
0.500	0.023	1.20	0.028	0.800	0.032
0.550	0.021	1.40	0.026	1.00	0.030
0.600	0.019	1.50	0.024	1.50	0.026
0.650	0.017	1.75	0.022		
0.700	0.015	2.00	0.021		
0.750	0.014	2.50	0.02		
0.800	0.014	3.00	0.02		
0.850	0.013	3.50	0.02		
0.900	0.013	4.00	0.02		
0.950	0.012				
1.0	0.011				
1.5	0.011				
2.0	0.011				
3.0	0.011				
4.0	0.011				
5.0	0.011				

a George Hass, Engineer Research and Development Laboratories, American Institute of Physics Handbook, McGraw-Hill, NY (1957) pp. 6–108.

b Ibid. pp. 6–110.

c Ibid. pp. 6–109.

**Table 6:** Real index of refraction and absorption constant of silver, as functions of wavelength

Wave-length μm	*n*	*k*	*R* %
400	0.075	1.93	
450	0.055	2.42	
500	0.071	3.020	97.3
550	0.069	3.429	97.9
600	0.072	3.348	98.2
650	0.080	4.257	98.4
700	0.093	4.645	98.4
750	0.103	5.005	98.4
800	0.110	5.409	98.6
850	0.121	5.757	98.6
900	0.128	6.089	98.7
950	0.130	6.476	98.8
1000	0.129	6.829	98.9

Optical Constants:

*N = n–ik* = complex index

*n* = (real) index of refraction

*k* = absorption constant (imaginary index)

*R* = reflectance (computed)

American Institute of Physics Handbook, McGraw-Hill, NY (1957) pp. 6–104.

**Table 7:** Low-temperature specific heat *C_P_*, and calorimetric Debye temperature, Θ_CAL_, for silver (purity: 99.999 %)

Temperature K	Specific heat J/(kg·K)	Specific heat J/(K·g-atom)	Specific heat cal/(K·g-atom)	Debey Θ_CALL_ (calorimetric) K
3	0.0598	0.006 45	0.001 541	226.6
4	0.1230	0.013 27	0.003 172	226.6
5	0.2234	0.024 10	0.005 760	226.6
6	0.3711	0.040 04	0.009 569	226.4
7	0.5786	0.062 43	0.014 92	225.8
8	0.8587	0.092 63	0.022 14	224.9
9	1.2256	0.1322	0.031 60	223.8
10	1.6980	0.1832	0.043 78	222.4
11	2.2879	0.2468	0.058 99	221.0
12	3.0158	0.3253	0.077 76	219.5
13	3.9055	0.4213	0.1007	217.9
14	4.9604	0.5351	0.1279	216.4
15	6.2093	0.6699	0.1601	214.9
16	7.6714	0.8276	0.1978	213.4
17	9.3081	1.0042	0.2400	212.3
18	11.1387	1.2016	0.2872	211.4
19	13.1361	1.4171	0.3387	210.8
20	15.3196	1.6527	0.3950	210.3
21	17.6816	1.9075	0.4559	209.9
22	20.1792	2.1769	0.5203	209.7
23	22.8165	2.4614	0.5883	209.6
24	25.6090	2.7627	0.6603	209.5
25	28.5100	3.0757	0.7351	209.5
26	31.4925	3.3974	0.8120	209.6
27	34.5758	3.7300	0.8915	209.7
28	37.7289	4.0702	0.9728	209.9
29	40.9557	4.4183	1.056	210.0
30	44.2136	4.7698	1.140	210.2

Douglas, L. Martin, Phys. Rev. **141**(2), 576–582 (1966).

**Table 8:** Thermodynamic functions for silver (for definitions of symbols, see text)

Temperature	Specific heat *C_p_*	Enthalpy *H*°*_T_*−*H*°_0_	Enthalpy function (*H*°*T*−H°_0_)/*T*	Entropy *S*	Gibbs energy −(*G*°_T_− *H*°_0_)/*T*	Gibbs energy function−(*G*°*_T_*− *H*°_0_)/*T*
K	J/(kg·K)	J/kg	J/kg	J/(kg·K)	J/kg	J/(kg·K)
1.0	0.00758	0.00340	0.00340	0.00654	0.00314	0.00314
2.0	0.02457	0.01830	0.00915	0.0162	0.01413	0.00706
3.0	0.06026	0.05868	0.0196	0.0322	0.03760	0.01253
4.0	0.1242	0.14796	0.0370	0.0574	0.08147	0.02037
5.0	0.2253	0.31937	0.0639	0.0955	0.1567	0.03133
6.0	0.3736	0.61435	0.1024	0.1483	0.2770	0.04617
7.0	0.5803	1.0837	0.1548	0.2206	0.4601	0.06573
8.0	0.8594	1.8022	0.2253	0.3161	0.7261	0.09076
9.0	1.224	2.8367	0.3152	0.4376	1.1013	0.1244
10.0	1.696	4.2829	0.4283	0.5896	1.6131	0.1613
11.0	2.290	6.2633	0.5694	0.7778	2.2944	0.2086
12.0	3.013	8.9107	0.7426	1.010	3.1816	0.2651
13.0	3.903	12.346	0.9497	1.279	4.3241	0.3326
14.0	4.960	16.768	1.198	1.613	5.7625	0.4116
15.0	6.211	22.332	1.489	1.993	7.5591	0.5039
16.0	7.657	29.250	1.828	2.438	9.7747	0.6109
17.0	9.289	37.704	2.218	2.948	12.455	0.7326
18.0	11.115	47.891	2.661	3.532	15.691	0.8717
19.0	13.108	59.993	3.158	4.190	19.551	1.0290
20.0	15.268	74.173	3.709	4.913	24.092	1.2046
25.0	28.423	181.86	7.274	9.669	59.757	2.3903
30.0	44.257	362.85	12.095	16.223	123.76	4.1253
35.0	61.296	626.48	17.899	24.316	224.53	6.4151
40.0	78.048	975.25	24.381	33.605	368.85	9.2213
45.0	93.724	1405.02	31.223	43.710	561.84	12.485
50.0	108.09	1910.17	38.203	54.343	806.90	16.138
55.0	120.89	2483.59	45.156	65.264	1105.9	20.108
60.0	132.29	3117.09	51.951	76.277	1459.6	24.327
65.0	142.30	3804.25	58.527	87.272	1868.6	28.748
70.0	151.11	4537.96	64.828	98.174	2332.3	33.318
75.0	158.89	5315.55	70.847	108.83	2850.0	37.999
80.0	165.66	6125.15	76.564	119.31	3420.3	42.753
85.0	171.78	6969.10	81.989	129.51	4042.4	47.557
90.0	177.16	7841.94	87.133	139.52	4715.3	52.393
95.0	181.98	8739.96	92.000	149.25	5437.4	57.236
100.0	186.34	9659.78	96.598	158.71	6207.5	62.075
105.0	190.23	10603.2	100.98	167.89	7024.0	66.895
110.0	193.66	11563.9	105.13	176.79	7885.7	71.688
115.0	196.81	12540.5	109.05	185.50	8791.0	76.444
120.0	199.68	13530.7	112.76	193.94	9739.7	81.164
125.0	202.28	14531.4	116.25	202.10	10730	85.840
130.0	204.60	15558.5	119.68	210.07	11761	90.470
135.0	206.82	16582.5	122.83	217.85	12828	95.022
140.0	208.77	17624.9	125.89	225.46	13939	99.564
145.0	210.62	18671.1	128.77	232.78	15082	104.01
150.0	212.29	19729.3	131.53	239.92	16270	108.46
155.0	213.87	20792.2	134.14	246.96	17487	112.82
160.0	215.26	21863.4	136.65	253.73	18734	117.09
165.0	216.65	22944.3	139.06	260.41	20023	121.35
170.0	217.85	24030.4	141.36	266.90	21339	125.52
175.0	219.06	25123.3	143.56	273.20	22696	129.69
180.0	220.17	26214.9	145.64	279.41	24079	133.77
185.0	221.19	27320.4	147.68	285.44	25485	137.76
190.0	222.12	28428.7	149.62	291.37	26924	141.71
195.0	223.05	29543.8	151.51	297.12	28399	145.64
200.0	223.97	30666.5	153.33	302.77	29895	149.48
205.0	224.71	31775.3	155.00	308.33	31428	153.30
210.0	225.55	32900.7	156.67	313.80	32984	157.07
215.0	226.29	34042.8	158.34	319.09	34561	160.75
220.0	227.03	35175.1	159.89	324.28	36172	164.42
225.0	227.68	36314.5	161.40	329.38	37804	168.02
230.0	228.33	37449.9	162.83	334.38	39467	171.60
235.0	228.89	38590.8	164.22	339.30	41153	175.12
240.0	229.54	39745.6	165.61	344.12	42860	178.59
245.0	230.09	40891.6	166.90	348.85	44594	182.02
250.0	230.65	42041.3	168.17	353.57	46343	185.37
255.0	231.11	43189.5	169.37	358.12	48121	188.71
260.0	231.67	44349.7	170.58	362.57	49927	192.03
265.0	232.13	45512.1	171.74	367.02	51752	195.29
270.0	232.59	46671.2	172.86	371.37	53600	198.52
273.2	232.87	47413.2	173.58	374.06	54772	200.52
275.0	233.06	47836.3	173.95	375.64	55464	201.69
280.0	233.52	49007.1	175.03	379.81	57355	204.84
285.0	233.99	50172.9	176.05	383.98	59262	207.94
290.0	234.36	51338.1	177.03	388.06	61194	211.01
295.0	234.73	52518.6	178.03	392.05	63138	214.03
298.2	235.01	53261.8	178.64	394.55	64381	215.94
300.0	235.19	53695.2	178.98	396.03	65114	217.05

G. T. Furukawa, W. G. Saba, and M. L. Reilly, Natl. Stand. Ref. Data Series 18, Natl. Bur. Stand. (U.S) (1968).

**Table 9:** Adiabatic elastic coefficient for silver^a^ Shear constants: *C* = *C*_44_ *C*′ = (*C*_11_ − *C*_12_)/2 Longitudinal modulus: *C*_L_ = (*C*_11_ + C_12_ + 2*C*_44_)/2 Bulk modulus: *B* = (*C*_11_ + 2*C*_12_)/3 Young’s modulus: *E*

Temperauture	*C*_11_	*C*_12_	*C*=*C*_44_	C′	*B*	*C*_L_	*E*^b^
K	GPa	GPa	GPa	GPa	GPa	GPa	GPa
0	131.49	97.33	51.09	17.08	108.72	165.50	91.23
10	131.44	97.30	51.08	17.07	108.68	165.45	91.21
25	131.41	97.33	50.98	17.04	108.69	165.35	91.05
50	131.12	97.26	50.72	16.93	108.55	164.91	90.55
75	130.54	97.04	50.28	16.75	108.21	164.07	89.82
100	129.80	96.66	49.82	16.57	107.71	163.05	88.979
125	129.08	96.28	49.36	16.40	107.21	162.04	88.154
150	128.35	95.91	48.90	16.22	106.72	161.03	87.327
175	127.62	95.54	48.44	16.04	106.23	160.02	86.500
200	126.91	95.17	47.97	15.87	105.75	159.01	85.681
225	126.19	94.81	47.50	15.69	105.27	158.00	84.843
250	125.46	94.44	47.04	15.51	104.78	156.99	84.015
275	124.73	94.05	46.58	15.34	104.28	155.97	83.202
300	123.99	93.67	46.12	15.16	103.78	154.95	82.372

a J. R. Neighbours and G. A. Alers, Phys. Rev. **111**, 707 (1958).

b H. Ledbetter, private communication (Ref.134).

**Table 10:** Properties of cryogenic fluids^a^

		Liquid at normal boiling point				Vapor at 1.013×10^5^ Pa (1 atm)			

Fluid	Atomic mass u	Temp.	Density	Enthalpy	Spec. ht. *C_P_*	Temp.	Density	Enthalpy	Spec. ht. *C_P_*
		K	mol/L	J/mol	J/(mol·K)	K	mol/L	J/mol	J/(mol·K)
Helium	4.0026	4.222	31.20	−21.0	21.10	4.222	4.226	62	36.73
						20.27	0.601	419	21.01
						27.09	0.449	562	20.91
						77.37	0.157	1609	20.80
						90.20	0.135	1876	20.79
						273.15	0.045	5679	20.79
Hydrogen (para)	2.0159	20.27	35.12	−516	19.49	20.27	0.664	382	24.68
						27.09	0.471	539	22.15
						77.37	0.158	1632	23.36
						90.20	0.135	1944	25.38
						273.15	0.045	7657	30.36
Neon^b^	20.180	27.09	59.71	−1203	38.8	27.09	0.476	525	24.1
						77.37	0.158	1602	20.9
						90.20	0.135	1869	20.9
						273.15	0.045	5676	20.8
Nitrogen	28.134	77.37	28.86	−3401	57.79	77.37	0.165	2164	31.45
						90.20	0.139	2559	30.43
						273.15	0.045	7934	29.15
Oxygen	31.999	90.20	35.66	−4263	54.22	90.20	0.140	2535	31.35
						273.15	0.045	7937	29.33

a NIST Standard Database 12: NIST Thermophysical Properties of Pure Fluids, (Version 3.0), D. G. Friend (NIST, Gaithersburg, MD, 1992), temperatures converted to ITS-90.

b Based on a preliminary NIST thermodynamic surface.
